# Direct synthesis of anomeric tetrazolyl iminosugars from sugar-derived lactams

**DOI:** 10.3762/bjoc.17.12

**Published:** 2021-01-13

**Authors:** Michał Mateusz Więcław, Bartłomiej Furman

**Affiliations:** 1 Institute of Organic Chemistry, Polish Academy of Sciences, Kasprzaka 44/52, 01-224, Warsaw, Poland

**Keywords:** amide functionalization, iminosugars, Schwartz’s reagent, tetrazole

## Abstract

Herein we present the direct asymmetric synthesis of tetrazole-functionalized 1-deoxynojirimycin derivatives from simple sugars via a Schwartz’s reagent-mediated reductive amide functionalization followed by a variant of the Ugi–azide multicomponent reaction. The anomeric configurations of two products were unambiguously confirmed by X-ray analysis. This work also describes examples of interesting further transformations of the title products. Finally, some surprising observations regarding the mechanism of their formation were made.

## Introduction

The transformation of an amide into another chemical moiety in a controlled manner is not a trivial task. Although the Vilsmeier–Haack reaction [1] or amide reduction with LiAlH_4_ are textbook examples that easily come to mind, there are not many other methods available. Simple alkyl and aryl amides, unlike other carbonyl compounds, typically do not undergo direct addition by a nucleophile, including active organometallic compounds. For this reason, it has been chemists’ long-lasting ambition to develop a reliable, mild, and selective methodology for amide functionalization [2]. Even though a tremendous amount of work has been already done towards this matter, it is still a highly active field of research. Several review articles have been written about this topic, enclosing most of the advances made to date [3–5].

A fascinating subset of these transformations encompass the reduction of amides to imines, with direct subsequent functionalization. One of the methodologies for such a modification was developed by Charette et al. In their procedure the combination of triflic anhydride and pyridine [6] (or its 2-fluoro derivative [7]) was used as an activating agent to transform amides into reactive iminium complexes. Another stoichiometric approach was presented by Georg et al. by utilization of zirconocene chloride hydride, known as Schwartz’s reagent [8]. This reduces the amide moiety, giving a complex that can be readily transformed into an imine or iminium cation [9].

It may perhaps be observed without straying too far afield from our primary focus that reduction of amides is actually not a leading use case of Schwartz’s reagent. It is employed principally for hydrozirconation of double and triple carbon–carbon bonds [10], and its application in chemistry of amides is quite recent. It can be also utilized in reduction of other unsaturated moieties, e.g., Pace et al. have shown reduction of isocyanates to formamides [11] and reduction of isothiocyanates to thioformamides [12] by its means.

There have also been some catalytic protocols developed for the reduction of amides to imines. The most notable examples incorporate iridium complexes and silanes [13–14]. Cheng and Brookhart showed that the chlorobis(cyclooctene)iridium dimer ([Ir(coe)_2_Cl]_2_) can act as the catalyst in combination with Et_2_SiH_2_ [15]. Surprisingly, they were able to obtain imines as well as amines using this methodology. Based on the works of Nagashima [16], an iridium-based protocol for tertiary amides was introduced by Dixon [17–19] and Huang [20–21]. Adolfsson expanded this by the use of molybdenum-based catalysts [22]. The reductive approach allows the issues associated with nucleophilic addition to amide carbonyl groups to be overcome and as such is finding its place in a growing number of synthetic applications [10].

**Scheme 1:**
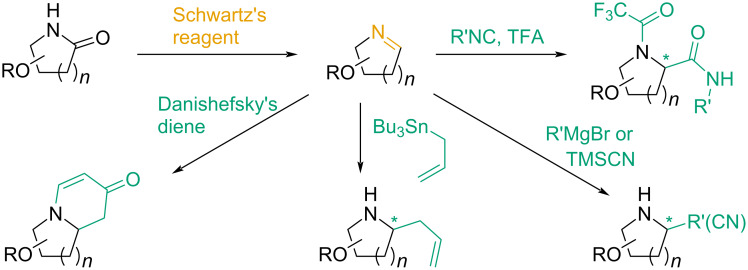
Our previous eﬀorts in the field of functionalization of sugar-derived lactams.

The employment of these methods for modification of lactams is a challenge in its own right – there are hardly any examples of such transformations available in the literature [23]. Our group was the first to surmount this challenge by means of Schwartz’s reagent-mediated reductive functionalization. Since then, we have performed a number of diﬀerent functionalizations of such cyclic systems with various complexity, and with a particular focus on the modification of sugar-derived lactams. As summarized in Scheme 1, this includes simple nucleophile addition to in situ-generated imines [23], the consecutive one-pot Mannich/Michael sequence leading to oligocyclic compounds [24], and employment in subsequent Joulié–Ugi multicomponent reactions [25].

**Figure 1:**
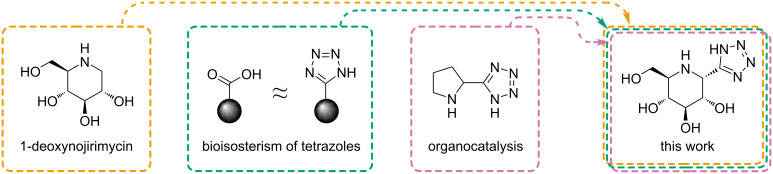
Key concepts behind the goal of this work [34].

This work is an extension of these eﬀorts and seeks to investigate the possibility of incorporating the Ugi–azide multicomponent reaction in this workflow. A molecule incorporating both an iminosugar and a tetrazole fragment is of particular interest, due to the interesting properties of both moieties (Figure 1). It is probably hard to overestimate the importance of sugar scaﬀolds in nature, and we believe that it speaks for itself, however, a significance of iminosugar derivatives may be less obvious. Several pharmaceuticals are based on this scaﬀold including the glucose-derived nojirimycin, an antibiotic and glycosidase inhibitor [26] and 1-deoxygalactonojirimycin, known under the trade name Galafold^®^, which is utilized for the treatment of the Fabry disease, a rare genetic condition [27]. On the other hand, the tetrazole moiety is known to have a bioisosteric relationship to carboxylic acids [28], which also makes them suitable for usage as biologically active compounds. Vasella et al., for example, have previously prepared compounds similar to reported ones in this work – fused iminosugar-tetrazoles – which have shown inhibition properties against bovine liver α-ᴅ-glucuronidase and human β-ʟ-iduronidase [29]. Moreover, there are numerous reports of the organocatalytic activity of chiral aminotriazoles and aminotetrazoles in number of reactions, such as the aldol reaction [30], Michael addition [31], Mannich reaction [32], and hydrogenation [33].

## Results and Discussion

**Scheme 2:**
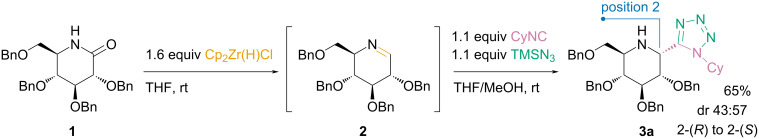
Preliminary experiment in search of a procedure for the synthesis of 2-(1*H*-tetrazol-5-yl)-iminosugars.

Quite recently Xie and Dixon showed that it is possible to synthesize α-tetrazolylamines from simple and linear tertiary amides using an iridium-based catalytic protocol [19]. They have, however, only reported one example of lactam functionalization which only proceeded with moderate eﬃciency (1-*tert*-butylazepan-2-one, 41% yield of the desired product). Unfortunately, this approach cannot be utilized for the functionalization of secondary amides, like sugar-derived lactams, due to the aforementioned method’s limitation to tertiary amides. Our previous work shows that Charette’s methodology is also not applicable in this case, as it does not lead to the formation of an imine [23]. Luckily, we were able to use a formerly established strategy based on Georg’s procedure with standard Ugi–azide reaction conditions [35–39] in a one-pot, tandem process. Subjecting glucose-derived lactam **1** to such a procedure gave the desired product in good yield, but with virtually no diastereoselectivity, as shown in Scheme 2.

### Optimization and scope

An initial optimization study for the proton donor for TMSN_3_ activation (shown in Table 1) using commonly encountered reagents for such reactions was performed. To our surprise, we observed the formation of the Ugi–azide product even in the absence of a protic additive. Moreover, the aprotic conditions proved to provide the highest yield and diastereoselectivity, thus were chosen as optimal (Table 1, entry 9.). We also tried to isolate the imine after the reduction step and carry out the second step in a solvent commonly used for the Ugi–azide reaction alone. For this, we observed a significant decrease in overall yield and suspect that the low stability of imines of type **2** may be the reason for this behavior.

**Table 1:** Optimization of 2-(1*H*-tetrazol-5-yl)-iminosugar synthesis via Schwartz’s reagent-mediated reduction of amides and Ugi–azide reaction.

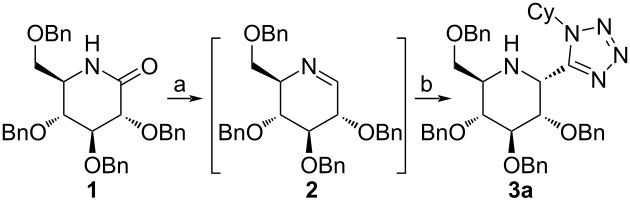				

Entry	Additive	Solvent	Yield [%]	dr^c^

1	MeOH^d^	THF	65	43:57
2	CF_3_CO_2_H	THF	24	43:57
3	AcOH	THF	47	80:20
4	Et_3_N · HCl	THF	45	74:26
5	H_2_O	THF	34	>95:5
6	(CF_3_)_2_CHOH	THF	35	>95:5
7	none	MeOH	19^e^	>95:5
8	none	DCM	36^e^	>95:5
**9**	**none**	**THF**	**73**	**>95:5**

^a^1.6 equiv Cp_2_Zr(H)Cl in THF under argon atmosphere; ^b^1.6 equiv of additive (if applicable), 1.1 equiv CyNC, and 1.1 equiv TMSN_3_. ^c^2-(*R*) to 2-(*S*), isolated. ^d^Additive used in excess. ^e^The imine was isolated after reduction.

The established optimal conditions were applied for the synthesis of selected examples of various 2-(1*H*-tetrazol-5-yl)-iminosugars (Table 2). Attempts at using this methodology to synthesize pentose-derived 2-(tetrazol-5-yl)-iminosugars, using 2,3,5-tri-*O*-benzyl-ᴅ-ribofuranose- and -arabinofuranose-derived lactams as substrates were made. Very unexpectedly, we failed to isolate such products although we did observe their formation via mass spectrometry of the reaction mixtures. Employing alternative procedures did not help, and none of the desired products were observed at all when applying iridium complex- or triflic anhydride-based methods.

**Table 2:** Synthesis of 2-(1*H*-tetrazol-5-yl)-iminosugars using optimized conditions. Reaction yield and dr are given.

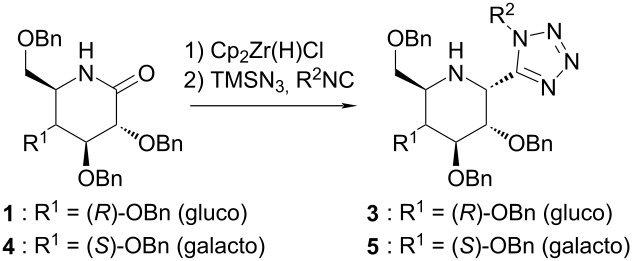				

Entry	Product	R^2^	Yield [%]	dr^a^

1	**3a**	Cy	73	>95:5
2	**3b**	CH_2_CO_2_Et	49	>95:5
3	**3c**	Bn	18	>95:5
4	**3d**	PMP	29	79:21
5	**3e**	PMB	42	>95:5
6	**3f**	*tert*-butyl	40	>95:5
7	**3g**	*tert*-octyl	48	>95:5
8	**5a**	Cy	33	>95:5
9	**5b**	CH_2_CO_2_Et	16	>95:5

^a^2-(*R*) to 2-(*S*), isolated.

The methodology described here provides a pathway to new, interesting compounds, containing both an iminosugar and tetrazole moiety. Such compounds have not been seen to date, and their accessibility creates exciting synthetic opportunities. Here we present two examples of possible further transformations of the products obtained over the course of this research directed towards novel, attractive molecules.

**Scheme 3:**
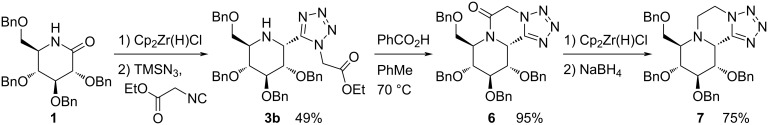
Synthesis of a new class of alkaloid scaﬀold using the presented methodology.

Compound **3b** underwent a cyclization reaction in the presence of benzoic acid at an elevated temperature yielding lactam **6** almost quantitatively. The deoxygenative reduction of this compound turned out to be challenging, as the typical procedure using LiAlH_4_ proved ineﬀective. We were able to obtain **7** using a Schwartz’s reagent-mediated amide activation methodology followed by NaBH_4_ reduction. This structure with three condensed rings can be seen as a new class of unnatural, chiral alkaloid scaﬀolds, potentially exhibiting pharmacological activity (Scheme 3) [40].

**Scheme 4:**
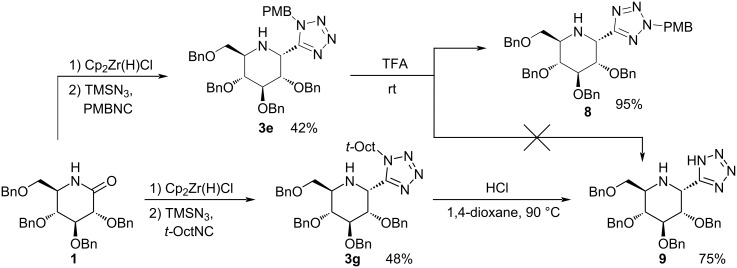
Synthesis of a new, chiral 2-(tetrazol-5-yl)-iminosugar based potential organocatalyst.

Various unsuccessful attempts were made to deprotect compound **3e**. Unexpectedly, however, one of those experiments resulted in a rearrangement in the tetrazole ring, as shown in Scheme 4, upper path. We were able to obtain the desired aminotetrazole **9** by treating **3g** with dry HCl at elevated temperature (Scheme 4, lower path). The resulting compound is particularly appealing, as similar scaﬀolds are widely used as organocatalysts. Such moieties are employed in a number of important synthetic transformations, including the aldol reaction [30], Michael addition [31], Mannich reaction [32], and hydrogenation [33]. We plan to test these possibilities in the near future.

### Stereochemistry and configuration of products

As presented in Table 2, only one diastereomer of the desired iminosugar is obtained in almost all cases. This outstanding selectivity has been observed before and is described in our previous works devoted to the functionalization of sugar-derived lactams [23–25]. We explain it in the light of Woerpel’s model, which characterizes the direction of nucleophilic addition to oxocarbenium ions [41–43]. According to this concept, the conformational stability of the compound in question is the key property to consider when predicting the reaction’s stereoselectivity.

**Scheme 5:**
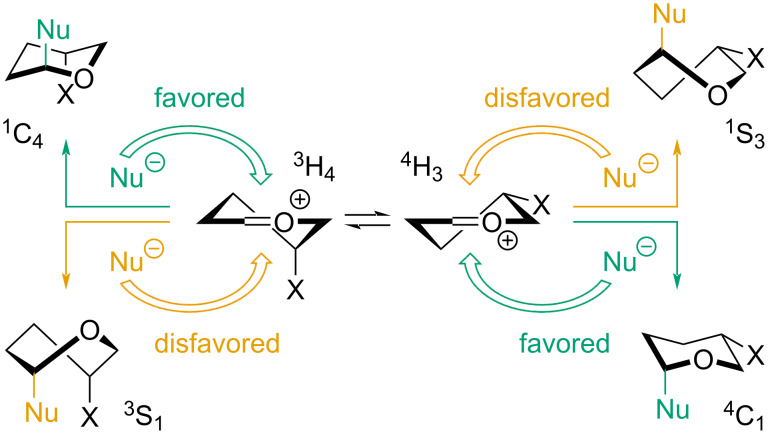
Principle behind Woerpel’s model for prediction of the direction of nucleophile addition to oxocarbenium cations.

When the oxocarbenium ion is substituted, two diastereomeric half-chair conformers are possible: ^3^H_4_ and ^4^H_3_ (shown for a 4-substituted pyranose cation in Scheme 5). Both may undergo attack by a nucleophile in two ways: on the axial trajectory from the top or the bottom face. Such an event would result in the formation of the product as a chair (^1^C_4_, ^4^C_1_) or a skew-boat (^1^S_3_, ^3^S_1_) conformer, of which the former is favored, as it proceeds via the lower-energetic chair-like transition state. The favored path of action will result in addition *syn* or *anti* to the substituent in position 5, depending on the starting conformer. Therefore, once the ground conformer of the oxocarbenium ion is established, this logic may be used to predict the reaction’s stereochemistry.

**Scheme 6:**
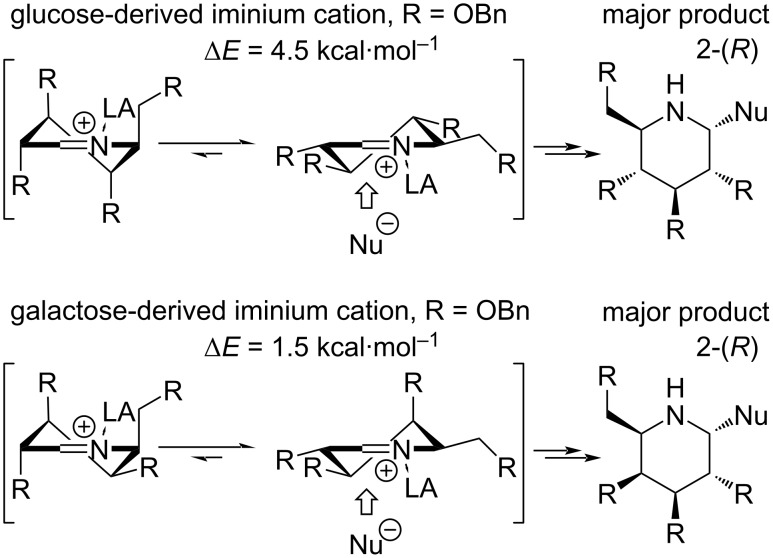
Diﬀerence in conformational stability of glucose- and galactose-derived iminium cations and the major product of the nucleophile attack according to Woerpel’s model [24].

The same principle may be successfully applied to reactions of iminium cations. We have previously shown that in the case of glucose- and galactose-derived, *O*-benzyl-protected iminosugars the addition *syn* to the substituent in position 3 is favored (Scheme 6). This work proves no diﬀerent, as the isolated major products were in such configuration. The experimental determination of this, however, was not straightforward in all cases.

**Figure 2:**
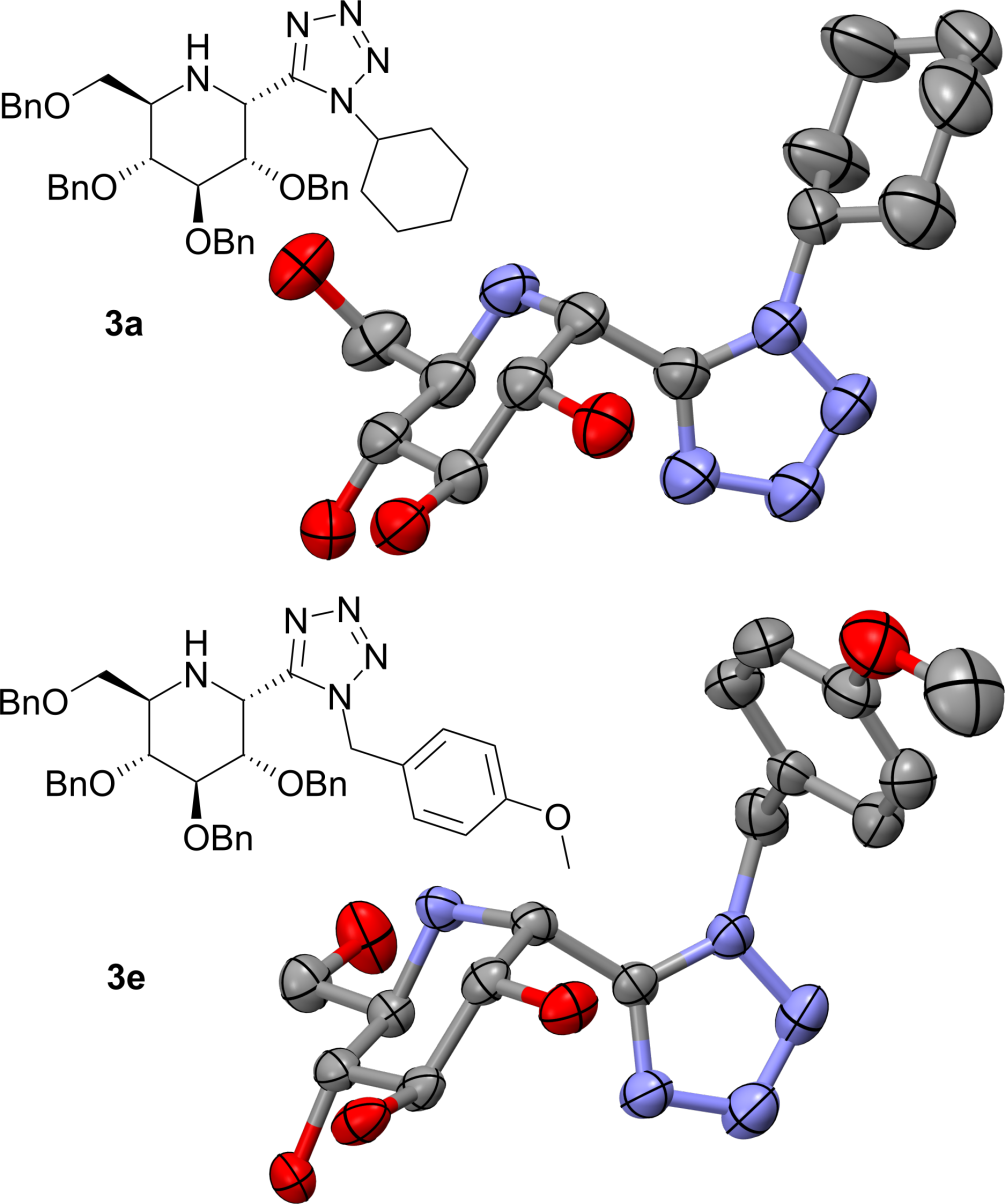
ORTEP structures of compounds **3a** and **3e** obtained by X-ray analysis. Hydrogen atoms and benzyl groups are omitted for clarity. Full crystallographic data available in Supporting Information File 1 and Supporting Information File 2, and in Cambridge Crystallographic Database under CCDC-2001373 and CCDC-2001372 numbers, respectively.

**Figure 3:**
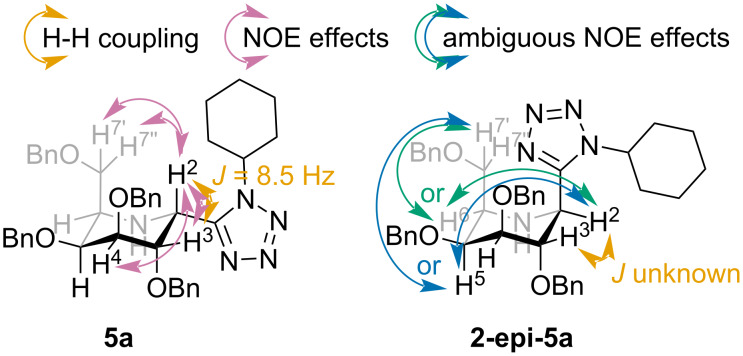
Proposed structures of compounds **5a** and **2-epi-5a** with ^1^H-^1^H couplings and NOE eﬀects shown.

We were able to determine the structure of compounds **3a** and **3e** unambiguously by means of X-ray analysis, as shown in Figure 2. The configuration of the remaining glucose based products **3** was easily determined by the analysis of ^1^H-^1^H coupling constants and NOE eﬀects. Unfortunately, the same approach was not possible in the case of compounds **5**, as ^1^H NMR spectroscopy showed indefinite results. In compound **5a** the coupling constant between protons H^2^ and H^3^ has a value of 8.5 Hz. This cannot be associated with a particular relative configuration without comparison with the corresponding coupling constant in **2-epi-5a**. But alas, this value is unknown, due to overlapping and broadening of the relevant signals in the ^1^H NMR spectrum of the compound in question. For the same reasons NOE eﬀects present in **2-epi-5a** cannot be accurately interpreted. However, analysis of the NOE eﬀects in **5a**, particularly a small eﬀect between protons H^2^ and H^7^, suggest that it may be the diastereomer 2-(*R*), as shown in Figure 3. This result would be in accordance with the previously mentioned Woerpel’s model. The structure of compound **5b** was assigned per analogiam, as diagnostic signals in ^1^H NMR spectrum were also overlapped.

We made an attempt at resolving this problem by means of the electronic circular dichroism (ECD) technique. We recorded an ECD spectrum of both compounds and compared it with simulated spectra, generated for both possible diastereomers (2-(*R*) and 2-(*S*)) using computational chemistry software. Unfortunately, we were not able to fit any of these simulations to the experimental data with suﬃcient certainty. For the inquisitive readers, this work is fully described in Supporting Information File 3.

### Mechanism of the reaction

**Scheme 7:**
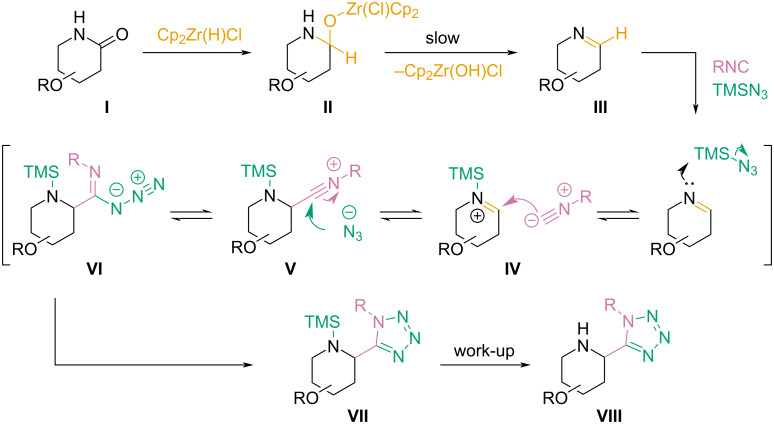
Proposed reaction mechanism for the described Ugi–azide reaction variant.

As mentioned previously, we observed Ugi–azide products, despite the absence of a proton donor in the reaction mixture. Intriguingly, this behavior is inconsistent with the generally accepted mechanism of this transformation, which assumes the hydrolysis of TMSN_3_ to HN_3_ and activation of the imine species by protonation. Scheme 7 presents our proposal for the possible course of the Ugi–azide reaction variant described in this work. We suppose that after reduction of amide **I** by Schwartz’s reagent, complex **II** undergoes a slow, spontaneous decomposition, yielding imine **III**. **III** then reacts with TMSN_3_, which acts as both, an imine activator and an azide anion source. Complex **IV** undergoes a subsequent addition of an isocyanide moiety (intermediate **V**), followed by an azide anion addition. Intermediate **VI** undergoes a cyclization, producing **VII**, a silylated derivative of the expected product. The hydrolysis of **VII** most likely occurs during the reaction’s work-up.

Preliminary DTF calculations were performed on a simplified model to provisionally validate this proposed mechanism. The geometry of the intermediate species were optimized with Gaussian 09 software [44], using the B3LYP/LANL2DZ theory level for Zr and B3LYP/6-31G(d,p) for other atoms, with GD3 empirical dispersion correction. The optimization was followed by a single-point energy calculation using the larger basis set Def2TZVP with a PCM solvatation model for THF, as implemented in the Gaussian software. Energy values reported are a sum of electronic and zero-point energies.

**Scheme 8:**
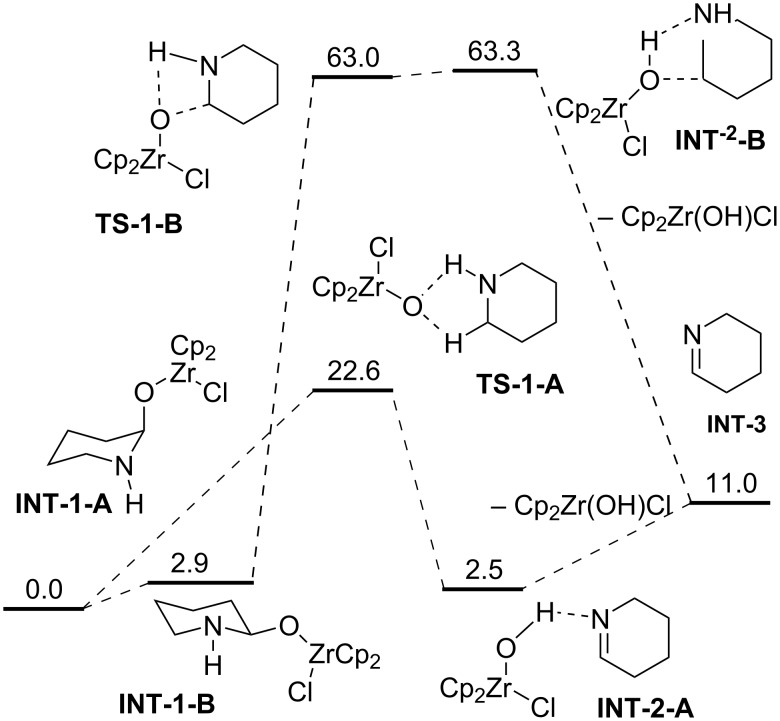
Possible pathway for spontaneous imine formation. Values reported are in kcal·mol^−1^.

Scheme 8 shows possible pathways for the spontaneous decomposition of the zirconium complex **INT-1-A** to the free imine species **INT-3**. This process is much more likely to occur via the 5-membered cyclic transition state **TS-1-A** than the alternative **TS-1-B**, as the energy barrier of 60.1 kcal·mol^−1^ is definitely too high for the reaction to take place, even at an elevated temperature. Path A with a barrier of 22.6 kcal·mol^−1^ is certainly more feasible. We assume that the Cp_2_Zr(OH)Cl species just leaves the initial complex, as this seems to be the simplest possibility in absence of any Lewis acid which could catalyze this decomposition.

**Scheme 9:**
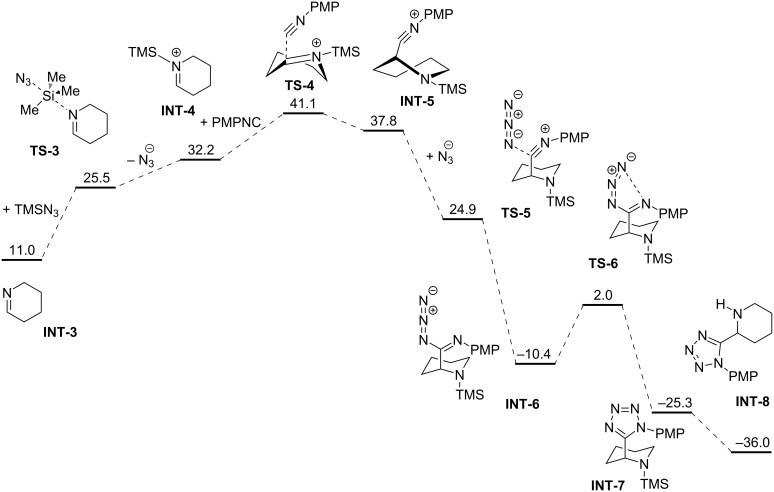
A possible path for tetrazole formation in the described conditions. Values reported are in kcal·mol^−1^.

Scheme 9 shows the energy diﬀerences in the subsequent steps of the examined reaction. The reported energy barriers are reasonably high for a slow process taking place at room temperature. The overall barrier is not considerably diﬀerent to those previously published for typical mechanisms of tetrazole formation by azide addition to nitriles [45]. It is important to note that the computational investigation of this reaction mechanism was not a primary goal of this work. That said, we consider this simple, crude DFT research to support our model of the transformation described herein.

## Conclusion

During the course of this research we have developed a methodology for the synthesis of sugar-derived α-tetrazolylamines. Such compounds – incorporating both iminosugar and tetrazole fragments – are particularly interesting, thanks to the well-known biological and catalytic activity of these moieties. This work is the first example of using Schwartz’s reagent-mediated partial reduction of lactams and the Ugi–azide multicomponent reaction in a tandem process. Yields of the described products are moderate to good, a satisfying result for such a multistep process. We have shown that such a reaction does not necessarily requires protic conditions, in opposition to what is generally agreed upon for these type of reactions. An alternative reaction mechanism is proposed and provisionally confirmed with DFT calculations. Moreover, selected α-tetrazolyl-iminosugars were subjected to further transformations, yielding new, potentially biologically active and organocatalytic compounds.

## Experimental

Experimental procedures and other data are available in Supporting Information File 3.

## Supporting Information

File 1 Experimental procedures, characterization data, ECD analyses for **5a** and **2-epi-5a**, calculations of appropriate ECD and UV spectra, crystallographic data for **3a** and **3e**, atomic coordinates, energies, and number of imaginary frequencies for computed stationary points, and copies of ^1^H and ^13^C NMR spectra.

File 2 CCDC-2001373; X-ray crystallographic data for compound **3a**.

File 3 CCDC-2001372; X-ray crystallographic data for compound **3e**.
