# Supplementary material for: Direct synthesis of anomeric tetrazolyl iminosugars from sugar-derived lactams
Source: Beilstein J Org Chem. 2021 Jan 13;17:115–23. doi: 10.3762/bjoc.17.12 (PMC7814180; doi:10.3762/bjoc.17.12)
Supplement: File 1 — Experimental procedures, characterization data, ECD analyses for 5a and 2-epi-5a, calculations of appropriate ECD and UV spectra, crystallographic data for 3a and 3e, atomic coordinates, energies, and number of imaginary frequencies for computed stationary points, and copies of 1H and 13C NMR spectra. [file Beilstein_J_Org_Chem-17-115-s001.pdf]

BEILSTEIN JOURNAL OF ORGANIC CHEMISTRY

Supporting Information

for

Direct synthesis of anomeric tetrazolyl iminosugars from sugar-derived lactams

Michał M. Więcław and Bartłomiej Furman

Beilstein J. Org. Chem. **2021,** *17,* 115–123. doi:10.3762/bjoc.17.12

Experimental procedures, characterization data, ECD analyses for 5a and 2-epi-5a, calculations of appropriate ECD and UV spectra, crystallographic data for 3a and 3e, atomic coordinates, energies, and number of imaginary frequencies for computed stationary points, and copies of ^{1}H and ^{13}C NMR spectra

License and Terms: This is a supporting information file under the terms of the Creative Commons Attribution License (https://creativecommons.org/licenses/by/4.0). Please note that the reuse, redistribution and reproduction in particular requires that the author(s) and source are credited and that individual graphics may be subject to special legal provisions.
The license is subject to the *Beilstein Journal of Organic Chemistry* terms and conditions: (https://www.beilstein-journals.org/bjoc/terms)

Contents

1 Experimental **S2**

1.1 General remarks S2

1.2 Synthetic procedures and characterization data S2

1.2.1 Synthesis of sugar-derived lactams. S2

1.2.2 Synthesis of glucose-derived α-tetrazolylamines (**3**). S3

1.2.3 Synthesis of galactose-derived α-tetrazolylamines (**5**). S8

1.2.4 Synthesis of derived compounds (**6**, **7**, **8** and **9**). S10

2 Crystallographic data of compounds 3a and 3e **S13**

2.1 Crystal structure of compound **3a** S14

2.2 Crystal structure of compound **3e** S15

3 Analysis of circular dichroism spectra of compounds 5a and 2-epi-5a **S16**

4 NMR spectra **S18**

5 Computational data **S51**

6 References **S69**

1 Experimental

1.1 General remarks

The reagents were purchased from Sigma Aldrich, Alfa Aesar or TCI Chemicals and used without further purification. All reactions involving air- and moisture-sensitive materials were carried out under an argon atmosphere in flame-dried glassware with magnetic stirring. THF was distilled from Na and benzophenone. CH_2Cl_2 was distilled from CaH_2. Column chromatography was performed with Kieselgel (230-400 mesh). Analytical TLC was performed with Silica gel 60 F254 aluminum plates (Merck) with visualization by UV light and charring with Pancaldi reagent ($(NH_4)_6MoO_4$, $Ce(SO_4)_2$, H_2SO_4, H_2O). NMR analyses were performed with Varian Mercury 400 MHz, Varian VNMRS 500 MHz and 600 MHz spectrometers. Chemical shifts (*δ*) are reported relative to tetramethylsilane (TMS). The residual signal of the solvent was used as an internal secondary reference ($CDCl_3$: $\delta_{^1\mathrm{H}} = 7.26$, $\delta_{^{13}\mathrm{C}} = 77.0$). Infrared (IR) spectra were recorded on a JASCO FT/IR-6200 spectrophotometer and are reported in frequency of absorption (cm^{-1}). HRMS spectra were recorded on an ESI-TOF Mariner spectrometer (Perspective Biosystem) and are given in *m*/*z*. Melting points were measured on a Melting Point Meter MPMH2 apparatus and are uncorrected.

1.2 Synthetic procedures and characterization data

1.2.1 Synthesis of sugar-derived lactams.

Sugar-derived lactams used in this work were prepared according to procedures published earlier [S1].

1.2.2 Synthesis of glucose-derived α-tetrazolylamines (3).

General Procedure: To a solid mixture of lactam **1** (107.4 mg, 0.20 mmol) and Schwartz's reagent (82.5 mg, 0.36 mmol, 1.6 equiv) 4 mL of dry THF was added at room temperature. The reaction mixture was stirred at rt until it cleared, typically for about 2 h. Then isocyanide was added (0.22 mmol, 1.1 equiv, dissolved in 0.5 mL of dry THF if it wasn't liquid), followed by $TMSN_3$ (29.1 µL, 0.22 mmol, 1.1 equiv). The reaction mixture was stirred overnight, if not stated otherwise. It was then evaporated using a rotary evaporator and purified by flash column chromatography in the appropriate solvent system to give a pure product.

(2*R*,3*S*,4*R*,5*R*,6*R*)-3,4,5-Tris(benzyloxy)-6-((benzyloxy)methyl)-2-(1-cyclohexyl-1*H*-tetrazol-5-yl)piperidine (**3a**)

The compound was synthesized according to the general procedure for glucose-derived α-tetrazoloamines **3** using 26.7 µL (0.22 mmol) of cyclohexyl isocyanide. It was purified using AcOEt in hexanes in 25% to 40% gradient as eluent and exceptionally using Florisil® as stationary phase. It was recrystallized from diethyl ether/hexanes mixture for X-ray analysis. **yield** 73% (white needles); **mp.** 166–167°C; $[\alpha_D^{23}]$ = 27.3 (c = 2:10, DCM); **^{1}H-NMR (500 MHz, $CDCl_3$):** δ (ppm) 7.58–6.95 (m, 20 H), 4.97–4.87 (m, 2 H), 4.90, 4.55 (ABq, *J* = 11:4 Hz, 2 H), 4.84, 4.58 (ABq, *J* = 12:1 Hz, 2 H), 4.67 (t, *J* = 9:1 Hz, 1 H), 4.39, 4.34 (ABq, *J* = 11:8 Hz, 2 H), 4.32 (d, *J* = 6:2 Hz, 1 H), 4.02–3.92 (m, 1 H), 3.84 (dd, *J* = 9:4; 6:2 Hz, 1 H), 3.61–3.54 (m, 1 H), 3.51 (t, *J* = 9:5 Hz, 1 H), 3.51–3.45 (m, 1 H), 3.43–3.34 (m, 1 H), 2.04–1.64 (m, 7 H), 1.31–1.10 (m, 3 H); **^{13}C-NMR (126 MHz, $CDCl_3$):** δ (ppm) 152.3, 138.7, 138.7, 138.3, 137.9, 128.5, 128.4, 128.4, 128.3, 128.0, 128.0, 127.9, 127.8, 127.8, 127.6, 127.6, 127.5, 83.2, 80.9, 80.4, 75.6, 74.6, 74.5, 73.2, 69.6, 57.6, 53.9, 49.8, 33.3, 32.5, 25.3, 25.3, 24.8; **IR (film)** 3334, 3087, 3062, 3030, 2934, 2861, 1953, 1874, 1811, 1604, 1586, 1496, 1453, 1362, 1292, 1247, 1209, 1095, 1068, 1028, 1002; **HRMS (ESI-TOF)** m/z calcd for $C_{41}H_{47}N_5O_4Na$: 696.3526 found: 696.3503

(2*S*,3*S*,4*R*,5*R*,6*R*)-3,4,5-Tris(benzyloxy)-6-((benzyloxy)methyl)-2-(1-cyclohexyl-1*H*-tetrazol-5-yl)piperidine (**2-epi-3a**)

The compound was synthesized according to the general procedure for glucose-derived α-tetrazoloamines **3**, but with addition of 0.5 µL of dry MeOH before addition of 26.7 µL (0.22 mmol) of cyclohexyl isocyanide. It was purified using AcOEt in hexanes in 30% to 40% gradient as eluent. **yield** 37% (white solid); **mp.** 153–154°C; $[\alpha_D^{23}]$ = 21.5 (c = 1:00, DCM); **^{1}H-NMR (600 MHz, $CDCl_3$):** δ (ppm) 7.62–7.12 (m, 20 H), 4.83, 4.74 (ABq, *J* = 11:1 Hz, 2 H), 4.78, 4.68 (ABq, *J* = 11:1 Hz, 2 H), 4.76, 4.46 (ABq, *J* = 11:1 Hz, 2 H), 4.58, 4.44 (ABq, *J* = 12:1 Hz, 2 H), 4.05–4.00 (m, 1 H), 3.83–3.76 (m, 1 H), 3.70–3.59 (m, 3 H), 3.53–3.47 (m, 1 H), 3.44–3.37 (m, 1 H), 3.03–2.98 (m, 1 H), 1.85–1.75 (m, 2 H), 1.62–1.50 (m, 3 H), 1.37–1.27 (m, 3 H), 1.19–1.01 (m, 1 H); **^{13}C-NMR (151 MHz, $CDCl_3$):** δ (ppm) 168.5, 137.3, 137.2, 137.1, 136.3, 127.5, 127.4, 127.3, 127.3, 127.2, 127.1, 127.0, 126.9, 126.9, 126.7, 126.7, 126.6, 81.7, 78.8, 78.6, 73.9, 73.6, 73.1, 71.9, 68.9, 55.1, 54.0, 46.6, 31.9, 24.5, 23.4; **IR (film)** 3340, 3087, 3062, 3030, 2928, 2854, 1951, 1874, 1809, 1666, 1529, 1496, 1453, 1363, 1311, 1252, 1208, 1070, 1028; **HRMS (ESI-TOF)** m/z calcd for $C_{41}H_{48}N_5O_4$: 674.3706 found: 674.3685

Ethyl (5-((2*R*,3*S*,4*R*,5*R*,6*R*)-3,4,5-tris(benzyloxy)-6-((benzyloxy)methyl)piperidin-2-yl)-1*H*-tetrazol-1-yl)acetate (**3b**)

The compound was synthesized according to the general procedure for glucose-derived α-tetrazoloamines **3** using 24.0 µL (0.22 mmol) of ethyl isocyanoacetate. It was purified using AcOEt in DCM in 3% to 7% gradient as eluent. **yield** 49% (white solid); **mp.** 94–95°C; $[\alpha_D^{23}]$ = 23.0 (c = 1:00, DCM); **^{1}H-NMR (600 MHz, $CDCl_3$):** δ (ppm) 7.35–7.03 (m, 20 H), 5.12, 4.61 (ABq, *J* = 17:7 Hz, 2 H), 4.85, 4.78 (ABq, *J* = 10:9 Hz, 2 H), 4.79, 4.45 (ABq, *J* = 11:2 Hz, 2 H), 4.71, 4.49 (ABq, *J* = 12:1 Hz, 2 H), 4.46–4.42 (m, 1 H), 4.33, 4.27 (ABq, *J* = 11:9 Hz, 2 H), 4.29 (d, *J* = 5:7 Hz, 1 H), 4.06 (q, *J* = 7:1 Hz, 2 H), 3.81 (dd, *J* = 8:9; 5:7 Hz, 1 H), 3.48 (dd, *J* = 9:4; 4:9 Hz, 1 H), 3.43 (dd, *J* = 9:7; 8:4 Hz, 1 H), 3.40 (dd, *J* = 9:4; 2:8 Hz, 1 H), 3.15 (ddd, *J* = 9:7; 4:9; 2:8 Hz, 1 H), 1.14 (t, *J* = 7:1 Hz, 3 H); **^{13}C-NMR (151 MHz, $CDCl_3$):** δ (ppm) 164.8, 153.1, 137.6, 137.5, 137.2, 136.8, 127.6, 127.4, 127.3, 127.3, 127.0, 127.0, 127.0, 126.8, 126.7, 126.7, 126.6, 126.5, 81.5, 79.5,

78.7, 74.4, 73.5, 73.1, 72.1, 68.4, 61.5, 52.9, 48.9, 47.1, 13.0; **IR (film)** 3337, 3087, 3062, 3030, 2982, 2908, 2868, 1955, 1877, 1811, 1751, 1604, 1496, 1453, 1396, 1373, 1308, 1211, 1094, 1069, 1027; **HRMS (ESI-TOF)** m/z calcd for $C_{39}H_{43}N_5O_6$: 678.3292 found: 678.3286

(2*R*,3*S*,4*R*,5*R*,6*R*)-3,4,5-Tris(benzyloxy)-6-[(benzyloxy)methyl]-2-(1-benzyl-1*H*-tetrazol-5-yl)piperidine (**3c**)

The compound was synthesized according to the general procedure for glucose-derived α-tetrazoloamines **3** using 26.8 µL (0.22 mmol) of benzyl isocyanide. It was purified using AcOEt in hexanes in 10% to 40% gradient as eluent. **yield** 18% (white solid); **mp.** 160–161°C; $[\alpha_D^{23}]$ = 19.4 (c = 1:07, DCM); **^{1}H-NMR (600 MHz, CDCl$_3$):** *δ* (ppm) 7.30–6.89 (m, 25 H), 5.44, 5.06 (ABq, *J* = 15:6 Hz, 2 H), 4.86, 4.81 (ABq, *J* = 10:8 Hz, 2 H), 4.80, 4.44 (ABq, *J* = 11:3 Hz, 2 H), 4.61, 4.35 (ABq, *J* = 12:3 Hz, 2 H), 4.59 (t, *J* = 9:2 Hz, 1 H), 4.26, 4.21 (ABq, *J* = 11:8 Hz, 2 H), 4.18 (d, *J* = 6:1 Hz, 1 H), 3.70 (dd, *J* = 9:4; 6:1 Hz, 1 H), 3.36–3.29 (m, 3 H), 3.12 (ddd, *J* = 9:7; 5:1; 2:8 Hz, 1 H); **^{13}C-NMR (151 MHz, cdcl$_3$):** *δ* (ppm) 153.1, 138.7, 138.5, 138.3, 137.8, 133.7, 129.2, 128.7, 128.6, 128.4, 128.3, 128.3, 128.0, 127.9, 127.9, 127.8, 127.8, 127.6, 127.5, 127.5, 127.1, 83.0, 80.6, 80.1, 75.7, 74.6, 73.9, 73.1, 69.6, 53.6, 50.7, 49.6; **IR (film)** 3334, 3087, 3062, 3031, 2923, 2858, 1954, 1875, 1811, 1731, 1680, 1604, 1496, 1453, 1361, 1313, 1259, 1208, 1069, 1028, 1002; **HRMS (ESI-TOF)** m/z calcd for $C_{42}H_{44}N_5O_4$: 682.3393 found: 682.3383

(2*R*,3*S*,4*R*,5*R*,6*R*)-3,4,5-Tris(benzyloxy)-6-((benzyloxy)methyl)-2-(1-(4-methoxyphenyl)-1*H*-tetrazol-5-yl)piperidine (**3d**)

The compound was synthesized according to the general procedure for glucose-derived α-tetrazoloamines **3** using 26.8 µL (0.22 mmol) of benzyl isocyanide. It was purified using 25% AcOEt in hexanes as eluent. **yield** 23% (white solid); **mp.** 156–157°C; $[\alpha_D^{23}]$ = 54.5 (c = 0:99, DCM); **^{1}H-NMR (600 MHz, CDCl$_3$):** *δ* (ppm) 7.35–7.14 (m, 20 H), 7.03–6.99 (m, 2 H), 6.91–6.86 (m, 2 H), 4.94 (ABq, *J* = 11:1 Hz, 2 H), 4.92, 4.56 (ABq, *J* = 11:4 Hz, 2 H), 4.85–4.79 (m, 1 H), 4.69, 4.43 (ABq, *J* = 12:2 Hz, 2 H), 4.40, 4.34 (ABq, *J* = 11:7 Hz, 2 H), 4.37 (d, *J* = 6:3 Hz, 1 H), 3.85 (s, 3 H), 3.72 (dd, *J* = 9:5; 6:3 Hz, 1 H), 3.60–3.51

(m, 2 H), 3.47 (d, J = 5:6 Hz, 2 H); **^{13}C-NMR (151 MHz, $CDCl_3$):** δ (ppm) 160.9, 153.5, 138.8, 138.7, 138.0, 137.9, 128.4, 128.4, 128.4, 128.3, 128.0, 127.8, 127.8, 127.7, 127.6, 127.5, 127.5, 127.5, 127.1, 126.3, 114.7, 83.2, 80.8, 80.4, 75.7, 74.7, 73.8, 73.2, 69.7, 55.7, 53.7, 49.2; **IR (film)** 3652, 3329, 3062, 3030, 2909, 2867, 2053, 1955, 1879, 1813, 1607, 1589, 1517, 1454, 1362, 1306, 1255, 1209, 1098, 1068, 1027; **HRMS (ESI-TOF)** m/z calcd for $C_{42}H_{44}N_5O_5$: 698.3342 found: 698.3341

(2*S*,3*S*,4*R*,5*R*,6*R*)-3,4,5-Tris(benzyloxy)-6-((benzyloxy)methyl)-2-(1-(4-methoxyphenyl)-1*H*-tetrazol-5-yl)piperidine (**2-epi-3d**)

The compound was synthesized according to the general procedure for glucose-derived α-tetrazoloamines **3** using 26.8 µL (0.22 mmol) of benzyl isocyanide. It was purified using 25% AcOEt in hexanes as eluent. **yield** 6% (yellow waxy oil); $[\alpha_D^{23}]$ = 8.7 (c = 0:27, DCM); **^{1}H-NMR (600 MHz, $CDCl_3$):** δ (ppm) 7.63–7.55 (m, 2 H), 7.49–7.28 (m, 20 H), 7.10–7.02 (m, 2 H), 4.88, 4.65 (ABq, J = 11:4 Hz, 2 H), 4.82 (s, 2 H), 4.80, 4.59 (ABq, J = 12:1 Hz, 2 H), 4.52–4.48 (m, 1 H), 4.43 (d, J = 5:1 Hz, 1 H), 4.41–4.36 (m, 1 H), 4.04 (t, J = 7:3 Hz, 1 H), 3.99 (s, 3 H), 3.81 (dd, J = 9:3; 5:0 Hz, 1 H), 3.57 (dd, J = 9:3; 4:1 Hz, 1 H), 3.05 (bs, 1 H); **^{13}C-NMR (151 MHz, $CDCl_3$):** δ (ppm) 160.8, 153.8, 138.5, 138.5, 138.3, 138.1, 128.4, 128.4, 128.3, 127.9, 127.8, 127.8, 127.8, 127.6, 127.6, 127.6, 127.5, 126.7, 126.6, 114.6, 79.0, 76.7, 76.0, 73.8, 73.1, 73.0, 72.8, 69.0, 55.6, 55.4, 48.9; **IR (film)** 3330, 3061, 3029, 2924, 2854, 1952, 1878, 1728, 1607, 1517, 1497, 1454, 1364, 1306, 1256, 1208, 1174, 1096, 1027; **HRMS (ESI-TOF)** m/z calcd for $C_{42}H_{44}N_5O_5$: 698.3342 found: 698.3348

(2*R*,3*R*,4*R*,5*S*,6*R*)-3,4,5-Tris(benzyloxy)-6-((benzyloxy)methyl)-2-(1-(4-methoxybenzyl)-1*H*-tetrazol-5-yl)piperidine (**3e**)

The compound was synthesized according to the general procedure for glucose-derived α-tetrazoloamines (**3**) using 32.4 mg (0.22 mmol) of 4-methoxybenzyl isocyanide dissolved in 0.5 mL of dry THF. It was purified using *tert*-butyl methyl ether in DCM in 0% to 3% gradient as eluent. **yield** 42% (white needles); **mp.** 139–142°C; $[\alpha_D^{23}]$ = 22.1 (c = 1:00, DCM); **^{1}H-NMR (600 MHz, $CDCl_3$):** δ (ppm) 7.33–7.18 (m, 18 H), 7.13–7.09 (m, 2

H), 6.97–6.94 (m, 2 H), 6.75–6.72 (m, 2 H), 5.43, 5.09 (ABq, *J* = 15:4 Hz, 2 H), 4.92, 4.88 (ABq, *J* = 10:8 Hz, 2 H), 4.87, 4.51 (ABq, *J* = 11:3 Hz, 2 H), 4.68, 4.42 (ABq, *J* = 12:3 Hz, 2 H), 4.65 (t, *J* = 9:2 Hz, 1 H), 4.34, 4.30 (ABq, *J* = 11:9 Hz, 2 H), 4.29 (d, *J* = 5:8 Hz, 1 H), 3.81–3.76 (m, 1 H), 3.70 (s, 3 H), 3.45–3.37 (m, 3 H), 3.24–3.18 (m, 1 H); **^{13}C-NMR (151 MHz, $CDCl_3$):** *δ* (ppm) 159.8, 152.9, 138.8, 138.6, 138.3, 137.8, 128.8, 128.6, 128.4, 128.4, 128.3, 128.1, 127.9, 127.8, 127.8, 127.8, 127.7, 127.6, 127.5, 114.6, 83.1, 80.7, 80.0, 75.7, 74.6, 73.9, 73.1, 69.5, 55.2, 53.7, 50.4, 49.6; **IR (film)** 3335, 3087, 3062, 3030, 2908, 2866, 1954, 1877, 1812, 1613, 1586, 1515, 1496, 1454, 1398, 1361, 1306, 1293, 1252, 1208, 1178, 1093, 1069, 1029, 1002; **HRMS (ESI-TOF)** m/z calcd for $C_{43}H_{46}N_5O_5$: 712.3499 found: 712.3484

(2*R*,3*R*,4*R*,5*S*,6*R*)-3,4,5-Tris(benzyloxy)-6-((benzyloxy)methyl)-2-(1-(*tert*-butyl)-1*H*-tetrazol-5-yl)piperidine (**3f**)

The compound was synthesized according to the general procedure for glucose-derived α-tetrazoloamines **3** using 22.6 µL (0.22 mmol) of *tert*-butyl isocyanide. The reaction was stirred for 12 d. It was purified using AcOEt in hexanes in 10% to 40% gradient as eluent. **yield** 40 % (white solid); **mp.** 164–165°C; $[\alpha_D^{23}]$ = 37.1 (c = 0:54, DCM); **^{1}H-NMR (600 MHz, $CDCl_3$):** *δ* (ppm) 7.32–6.96 (m, 20 H), 4.98 (t, *J* = 9:2 Hz, 1 H), 4.88, 4.87 (ABq, *J* = 10:8 Hz, 2 H), 4.85, 4.49 (ABq, *J* = 11:2 Hz, 2 H), 4.76, 4.53 (ABq, *J* = 12:0 Hz, 2 H), 4.68 (d, *J* = 6:4 Hz, 1 H), 4.30, 4.27 (ABq, *J* = 11:8 Hz, 2 H), 3.79 (dd, *J* = 9:5; 6:4 Hz, 1 H), 3.52–3.46 (m, 1 H), 3.43 (t, *J* = 9:5 Hz, 1 H), 3.40–3.37 (m, 1 H), 3.09 (ddd, *J* = 10:0; 4:7; 2:7 Hz, 1 H), 1.54 (s, 9 H); **^{13}C-NMR (151 MHz, $CDCl_3$):** *δ* (ppm) 152.4, 138.9, 138.7, 138.2, 137.9, 128.4, 128.4, 128.4, 128.3, 128.1, 127.7, 127.7, 127.7, 127.7, 127.6, 127.5, 127.4, 83.5, 81.4, 80.6, 75.7, 74.6, 74.3, 73.1, 69.7, 61.2, 53.2, 50.5, 30.2; **IR (film)** 3328, 3087, 3061, 3030, 2984, 2915, 2866, 1954, 1875, 1812, 1728, 1604, 1496, 1453, 1400, 1363, 1334, 1285, 1238, 1211, 1094, 1069, 1028; **HRMS (ESI-TOF)** m/z calcd for $C_{39}H_{46}N_5O_4$: 648.3550 found: 648.3542

(2*R*,3*S*,4*R*,5*R*,6*R*)-3,4,5-Tris(benzyloxy)-6-((benzyloxy)methyl)-2-(1-(*tert*-octyl)-1*H*-tetrazol-5-yl)piperidine (**3g**)

The compound was synthesized according to the general procedure for glucose-derived α-tetrazoloamines **3** using 40.9 µL (0.22 mmol) of *tert*-octyl isocyanide. The reaction was stirred for 3 d. It was purified using 20 % AcOEt in hexanes as eluent. **yield** 48 % (yellow oil); $[\alpha_D^{23}]$ = 24.2 (c = 0:83, DCM); **^{1}H-NMR (600 MHz, $CDCl_3$):** *δ* (ppm) 7.34–7.01 (m, 20 H), 5.07 (t, *J* = 9:2 Hz, 1 H), 4.89, 4.87 (ABq, *J* = 10:8 Hz, 2 H), 4.85, 4.50 (d, *J* = 11:3 Hz, 2 H), 4.70 (d, *J* = 6:5 Hz, 1 H), 4.74, 4.54 (ABq, *J* = 12:1 Hz, 2 H), 4.27, 4.27 (ABq, *J* = 12:1 Hz, 2 H), 3.77 (dd, *J* = 9:5; 6:4 Hz, 1 H), 3.49 (dd, *J* = 9:1; 4:6 Hz, 1 H), 3.45 (dd, *J* = 10:1; 8:9 Hz, 1 H), 3.35 (dd, *J* = 9:1; 2:7 Hz, 1 H), 3.02 (ddd, *J* = 10:1; 4:6; 2:7 Hz, 1 H), 2.06 (s, 1 H), 1.99 (d, *J* = 15:2 Hz, 1 H), 1.74 (s, 3 H), 1.74 (d, *J* = 15:2 Hz, 1 H), 1.50 (s, 3 H), 0.62 (s, 9 H); **^{13}C-NMR (151 MHz, $CDCl_3$):** *δ* (ppm) 152.2, 138.9, 138.8, 138.2, 137.9, 128.5, 128.4, 128.3, 128.3, 128.1, 127.8, 127.7, 127.7, 127.6, 127.5, 127.4, 127.0, 83.6, 81.5, 80.7, 75.7, 74.6, 74.1, 73.2, 69.5, 65.1, 53.7, 53.0, 50.7, 31.6, 30.8, 30.5, 30.0; **IR (film)** 3327, 3087, 3062, 3030, 2952, 2906, 2868, 1952, 1874, 1810, 1604, 1586, 1496, 1453, 1394, 1362, 1334, 1286, 1247, 1209, 1139, 1098, 1068, 1028, 1001; **HRMS (ESI-TOF)** m/z calcd for $C_{41}H_{55}N_5O_4Na$: 704.4152 found: 704.4152

1.2.3 Synthesis of galactose-derived α-tetrazolylamines (5).

General procedure: To a suspension of Schwartz's reagent (82.5 mg, 0.36 mmol, 1.6 equiv) in dry THF (2 mL), a solution of lactam **4** (107.4 mg, 0.20 mmol) in 2 mL of dry THF was added at room temperature. The reaction mixture was stirred at rt until it cleared, typically for about 15 min. Then isocyanide was added (0.22 mmol, 1.1 equiv, dissolved in 0.5 mL of dry THF if it wasn't liquid), followed by $TMSN_3$ (29.1 µL, 0.22 mmol, 1.1 equiv). The reaction mixture was stirred overnight, if not stated otherwise. It was then evaporated using a rotary evaporator and purified by flash column chromatography in the appropriate solvent system to give a pure product.

(2*R*,3*S*,4*R*,5*S*,6*R*)-3,4,5-Tris(benzyloxy)-6-((benzyloxy)methyl)-2-(1-cyclohexyl-1*H*-tetrazol-5-yl)piperidine (**5a**)

The compound was synthesized according to the general procedure for galactose-derived α-tetrazoloamines **5** using 26.7 µL (0.22 mmol) of cyclohexyl isocyanide. It was purified using a 2:28:70 mixture of Et_3N/AcOEt/hexanes as eluent. **yield** 33% (white solid); **mp.** 158–159°C; $[\alpha_D^{23}]$ = 33.4 (c = 1:01, DCM); **^{1}H-NMR (600 MHz, $CDCl_3$):** *δ* (ppm) 7.30–7.12 (m, 18 H), 6.97–6.91 (m, 2 H), 4.69, 4.33 (ABq, *J* = 10:8 Hz, 2 H), 4.60, 4.52 (ABq, *J* = 12:1 Hz, 2 H), 4.53–4.50 (m, 2 H), 4.42, 4.41 (ABq, *J* = 12:2 Hz, 2 H), 4.39 (t, *J* = 8:3 Hz, 1 H), 4.35–4.29 (m, 1 H), 4.12 (d, *J* = 8:5 Hz, 1 H), 3.88 (dd, *J* = 3:7; 2:9 Hz, 1 H), 3.84 (dd, *J* = 8:0; 2:6 Hz, 1 H), 3.56 (dd, *J* = 9:6; 5:1 Hz, 1 H), 3.50 (dd, *J* = 9:6; 5:6 Hz, 1 H), 3.20 (q, *J* = 5:0 Hz, 1 H), 1.94–1.49 (m, 7 H), 1.21–0.99 (m, 3 H); **^{13}C-NMR (151 MHz, $CDCl_3$):** *δ* (ppm) 154.2, 138.6, 138.5, 138.4, 138.1, 128.8, 128.7, 128.7, 128.6, 128.2, 128.2, 128.2, 128.2, 128.1, 128.0, 128.0, 127.7, 79.2, 75.2, 75.0, 73.8, 72.4, 72.2, 70.3, 58.1, 55.2, 51.8, 33.4, 32.8, 25.5, 25.5, 25.1; **IR (film)** 3328, 3087, 3062, 3030, 2933, 2860, 1952, 1872, 1811, 1670, 1604, 1496, 1453, 1365, 1261, 1207, 1097, 1027; **HRMS (ESI-TOF)** m/z calcd for $C_{41}H_{48}N_5O_4$: 674.3706 found: 674.3694

(2*R*,3*S*,4*R*,5*S*,6*R*)-3,4,5-Tris(benzyloxy)-6-((benzyloxy)methyl)-2-(1-cyclohexyl-1*H*-tetrazol-5-yl)piperidine (**2-epi-5a**)

The compound was synthesized according to the general procedure for galactose-derived α-tetrazoloamines (**5**), but with addition of 0.5 µL of dry MeOH before addition of 26.7 µL (0.22 mmol) of cyclohexyl isocyanide. It was purified by preparative HPLC using 10% acetone in toluene as eluent. **yield** 3% (white waxy oil); $[\alpha_D^{23}]$ = 21.8 (c = 0:17, DCM); **^{1}H-NMR (600 MHz, $CDCl_3$):** *δ* (ppm) 7.37–7.23 (m, 18 H), 7.16–7.12 (m, 2 H), 4.91, 4.56 (ABq, *J* = 11:3 Hz, 2 H), 4.79, 4.75 (ABq, *J* = 11:7 Hz, 2 H), 4.78, 4.59 (ABq, *J* = 11:7 Hz, 2 H), 4.48–4.45 (m, 1 H), 4.43, 4.40 (ABq, *J* = 11:9 Hz, 1 H), 4.32–4.25 (m, 1 H), 4.17–4.07 (bs, 1 H), 4.09 (t, *J* = 2:4 Hz, 1 H), 3.72 (td, *J* = 6:8; 2:1 Hz, 1 H), 3.52–3.44 (m, 1 H), 3.43–3.35 (m, 1 H), 2.00–1.91 (m, 1 H), 1.91–1.86 (m, 1 H), 1.86–1.74 (m, 3 H), 1.72–1.63 (m, 1 H), 1.25–1.11 (m, 4 H); **^{13}C-NMR (151 MHz, $CDCl_3$):** *δ* (ppm) 152.8, 138.8, 138.6, 138.3, 138.0, 128.4, 128.4, 128.3, 128.3, 128.1, 128.0, 127.8, 127.8, 127.7, 127.6, 127.5, 127.5, 76.6, 75.3, 74.4, 74.2, 73.2, 72.9, 69.5, 57.8, 53.2, 49.9, 33.2, 32.5, 25.3,

25.2, 24.8; **IR (film)** 3316, 3061, 3031, 2923, 2855, 1952, 1876, 1811, 1733, 1668, 1604, 1496, 1453, 1365, 1266, 1208, 1097, 1027; **HRMS (ESI-TOF)** m/z calcd for $C_{41}H_{48}N_5O_4$: 674.3706 found: 674.3707

Ethyl (5-((2*R*,3*S*,4*R*,5*S*,6*R*)-3,4,5-tris(benzyloxy)-6-((benzyloxy)methyl)piperidin-2-yl)-1*H*-tetrazol-1-yl)acetate (**5b**)

The compound was synthesized according to the general procedure for galactose-derived α-tetrazoloamines (**5**) using 26.7 µL (0.22 mmol) of cyclohexyl isocyanide. It was purified using 30% AcOEt in cyclohexane as eluent. **yield** 16% (white solid); **mp.** 144–145°C; $[\alpha_D^{23}]$ = −48.9 (c = 0:43, DCM); **^{1}H-NMR (400 MHz, $CDCl_3$):** *δ* (ppm) 7.38–7.13 (m, 20 H), 5.29, 5.26 (ABq, *J* = 17:3 Hz, 2 H), 4.74 (t, 1 H), 4.62 (s, 2 H), 4.53, 4.44 (ABq, *J* = 12:4 Hz, 2 H), 4.50, 4.46 (ABq, *J* = 12:1 Hz, 2 H), 4.40, 4.32 (ABq, *J* = 11:8 Hz, 2 H), 4.36–4.32 (m, 1 H), 4.14 (q, *J* = 7:1 Hz, 2 H), 3.91–3.85 (m, 2 H), 3.71 (dd, *J* = 9:3; 4:2 Hz, 1 H), 3.53 (dd, *J* = 9:3; 3:8 Hz, 1 H), 3.16–2.98 (m, 1 H), 2.41 (s, 1 H), 1.20 (t, *J* = 7:1 Hz, 3 H); **^{13}C-NMR (126 MHz, $CDCl_3$):** *δ* (ppm) 165.1, 153.8, 137.1, 136.9, 136.9, 136.8, 127.5, 127.4, 127.3, 127.3, 127.1, 126.9, 126.8, 126.8, 126.7, 126.6, 126.6, 126.5, 75.1, 72.4, 72.2, 72.1, 70.5, 70.1, 68.2, 61.1, 51.1, 50.5, 48.5, 13.1; **IR (film)** 3341, 3061, 3030, 2924, 2866, 1955, 1879, 1813, 1751, 1604, 1496, 1453, 1396, 1373, 1308, 1260, 1211, 1100, 1026; **HRMS (ESI-TOF)** m/z calcd for $C_{39}H_{43}N_5O_6$: 678.3292 found: 678.3298 1.2.4

1.2.4 Synthesis of derived compounds (6, 7, 8 and 9).

(8*R*,9*R*,10*R*,11*S*,11*aR*)-9,10,11-tris(benzyloxy)-8-((benzyloxy)methyl)-9,10,11,11a-tetrahydro-8*H*-pyrido[1,6-*a*]tetrazolo[5,1-*c*]pyrazin-6(5*H*)-one (**6**)

The compound was synthesized by the following procedure: 70 mg (0.10 mmol) of compound **3b** and 14.7 mg (0.12 mmol, 1.2 equiv.) of benzoic acid was dissolved in 5.0 mL of toluene. The mixture was heated to 70 °C and stirred for 16 h. It was then let to cool to room temperature, quenched with 10 mL saturated $NaHCO_{3(aq)}$ and extracted with AcOEt (2 × 10 mL). Organic phase was washed with water, dried over $MgSO_4$ and evaporated. The crude product was purified by flash column chromatography using 40%

tert-butyl methyl ether in hexanes as eluent. **yield** 95% (light yellow waxy oil); $[\alpha_D^{23}]$ = −10.8 (c = 1:00, DCM); **^{1}H-NMR (600 MHz, CDCl$_3$):** δ (ppm) 7.37–7.13 (m, 18 H), 6.80–6.77 (m, 2 H), 5.36–5.33 (m, 1 H), 5.29–5.25 (m, 1 H), 5.01 (dd, *J* = 17:3; 1:0 Hz, 1 H), 4.83 (dd, *J* = 17:3; 1:0 Hz, 1 H), 4.69, 4.53 (ABq, *J* = 11:5 Hz, 2 H), 4.51, 4.48 (ABq, *J* = 11:8 Hz, 2 H), 4.48, 4.39 (ABq, *J* = 12:1 Hz, 2 H), 4.34, 4.01 (ABq, *J* = 11:1 Hz, 2 H), 3.98 (t, *J* = 2:1 Hz, 1 H), 3.82 (t, *J* = 3:5 Hz, 1 H), 3.81–3.78 (m, 2 H), 3.68 (dd, *J* = 10:4; 5:7 Hz, 1 H); **^{13}C-NMR (151 MHz, CDCl$_3$):** δ (ppm) 161.6, 148.4, 137.6, 137.5, 136.9, 136.5, 128.7, 128.5, 128.4, 128.4, 128.3, 128.2, 128.1, 128.0, 127.9, 127.8, 127.8, 127.6, 77.5, 74.9, 73.0, 73.0, 72.6, 72.3, 72.3, 66.2, 52.1, 49.5, 47.9; **IR (film)** 3087, 3062, 3031, 2923, 2868, 1956, 1879, 1813, 1725, 1671, 1604, 1585, 1567, 1496, 1454, 1421, 1393, 1367, 1347, 1256, 1207, 1177, 1092, 1028; **HRMS (ESI-TOF)** m/z calcd for $C_{37}H_{37}N_5O_5Na$: 654.2692 found: 654.2670

(8*R*,9*S*,10*R*,11*S*,11a*R*)-9,10,11-tris(benzyloxy)-8-((benzyloxy)methyl)-5,6,9,10,11,11a-hexahydro-8*H*-pyrido[1,6-*a*]tetrazolo[5,1-*c*]pyrazine (**7**)

The compound was synthesized by the following procedure: 10 mg (0.016 mmol) of compound **6** was dissolved in 0.3 mL of dry THF and added to 6.6 mg (0.026 mmol, 1.6 equiv) of $Cp_2Zr(H)Cl$. The mixture was stirred at room temperature until it cleared (for about 4 h). 2.0 mg (0.048 mmol, 3.0 equiv) of $NaBH_4$ was added and mixture was stirred at rt overnight. Then 0.048 mL 1.0 m solution of BH_3·THF in THF (0.048 mmol, 3.0 equiv) was added. After 3 d of stirring at rt the reaction was quenched by 2 mL of water, extracted with AcOEt (3 × 5 mL), dried with $MgSO_4$ and evaporated. The crude product was purified by flash column chromatography using *tert*-butyl methyl ether in DCM in 10% to 40% gradient as eluent. **yield** 75% (yellow oil); $[\alpha_D^{23}]$ = 3.8 (c = 0:41, DCM); **^{1}H-NMR (600 MHz, CDCl$_3$):** δ (ppm) 7.34–7.24 (m, 12 H), 7.24–7.20 (m, 4 H), 7.18–7.14 (m, 2 H), 7.13–7.09 (m, 2 H), 4.58, 4.48 (ABq, *J* = 12:4 Hz, 2 H), 4.56 (d, *J* = 2:8 Hz, 1 H), 4.53 (ddd, *J* = 12:7; 8:5; 4:5 Hz, 1 H), 4.46–4.38 (m, 4 H), 4.40, 4.27 (ABq, *J* = 11:7 Hz, 2 H), 4.36 (dt, *J* = 12:5; 4:3 Hz, 1 H), 4.20–4.16 (m, 1 H), 3.80 (dd, *J* = 10:1; 6:3 Hz, 1 H), 3.67 (dt, *J* = 12:9; 4:4 Hz, 1 H), 3.61 (dt, *J* = 7:5; 3:2 Hz, 2 H), 3.58 (dd, *J* = 10:1; 5:5 Hz, 1 H), 3.41–

3.37 (m, 1 H), 3.18 (dt, *J* = 8:4; 4:4 Hz, 1 H); **^{13}C-NMR (151 MHz, $CDCl_3$):** *δ* (ppm) 150.8, 137.1, 136.8, 136.6, 136.4, 127.5, 127.5, 127.4, 127.4, 127.3, 126.9, 126.9, 126.8, 126.8, 126.7, 126.7, 126.6, 75.3, 73.0, 72.8, 72.6, 72.3, 71.5, 71.1, 66.1, 61.0, 50.6, 47.0, 44.2; **IR (film)** 3059, 3031, 2923, 2852, 1953, 1878, 1812, 1728, 1676, 1604, 1544, 1496, 1454, 1364, 1265, 1207, 1172, 1090, 1028; **HRMS (ESI-TOF)** m/z calcd for $C_{37}H_{39}N_5O_4Na$: 640.2900 found: 640.2892

(2*R*,3*S*,4*R*,5*R*,6*R*)-3,4,5-tris(benzyloxy)-6-((benzyloxy)methyl)-2-(2-(4-methoxyphenyl)-2*H*-tetrazol-5-yl)piperidine (**8**)

The compound was synthesized by the following procedure: 20.0 mg (0.028 mmol) of compound **3e** was dissolved in 0.5 mL of TFA and was stirred at rt for 24 h. It was then evaporated, dissolved in 20 mL of DCM, washed with 20 mL of $NaHCO_{3(aq)}$ and 20 mL of brine and then dried with $MgSO_4$ and evaporated. The crude product was purified by flash column chromatography using 5% of AcOEt in DCM as eluent. **yield** 95% (colourless oil); $[\alpha_D^{23}]$ = 49.6 (c = 0:87, DCM); **^{1}H-NMR (600 MHz, $CDCl_3$):** *δ* (ppm) 7.32–7.12 (m, 22 H), 6.84–6.79 (m, 2 H), 5.68 (ABq, *J* = 14:5 Hz, 2 H), 4.91 (d, *J* = 5:9 Hz, 1 H), 4.88, 4.52 (ABq, *J* = 11:0 Hz, 2 H), 4.87, 4.77 (ABq, *J* = 10:8 Hz, 2 H), 4.66, 4.57 (ABq, *J* = 11:5 Hz, 2 H), 4.36 (ABq, *J* = 11:7 Hz, 2 H), 4.38–4.32 (m, 1 H), 3.90 (dd, *J* = 9:2; 6:5 Hz, 1 H), 3.74 (s, 3 H), 3.63–3.58 (m, 1 H), 3.49–3.41 (m, 3 H); **^{13}C-NMR (151 MHz, $CDCl_3$):** *δ* (ppm) 165.2, 159.8, 138.9, 138.4, 137.9, 137.8, 129.7, 128.3, 128.2, 128.2, 128.2, 127.9, 127.9, 127.8, 127.8, 127.6, 127.5, 127.5, 127.4, 125.4, 114.2, 82.9, 80.4, 80.0, 75.5, 74.9, 73.0, 72.2, 70.2, 56.2, 55.2, 53.9, 50.9; **IR (film)** 3341, 3087, 3062, 3030, 3005, 2925, 2864, 2056, 1954, 1877, 1812, 1613, 1586, 1515, 1496, 1454, 1394, 1362, 1330, 1305, 1251, 1208, 1178, 1088, 1070, 1029; **HRMS (ESI-TOF)** m/z calcd for $C_{43}H_{46}N_5O_5$: 712.3499 found: 712.3477

(2*R*,3*S*,4*R*,5*R*,6*R*)-3,4,5-Tris(benzyloxy)-6-((benzyloxy)methyl)-2-(1*H*-tetrazol-5-yl)piperidine (**9**)

The compound was synthesized by the following procedure: 44.3 mg (0.063 mmol) of compound **3g** was dissolved in 6.0 mL of 4.0 m solution of HCl in dioxane and the flask

was closed tightly. The mixture was stirred at 90 °C for 24 h. It was then let to cool to room temperature, diluted with 10 mL of *tert*-butyl methyl ether and 10 mL of water. $NaHCO_3$ was slowly added while stirring until pH = 7 was reached, then the mixture was separated. Aqueous phase was extracted with *tert*-butyl methyl ether (2 × 5 mL). Combined organic phases were dried with $MgSO_4$ and evaporated. The crude product was purified by flash column chromatography using $NH_{3(aq)}$/MeOH/DCM in 0:10:90 to 1:20:80 gradient as eluent. **yield** 75% (light grey solid); **mp.** 180–183°C; $[\alpha_D^{23}]$ = 57.2 (c = 0:60, DCM); **^{1}H-NMR (600 MHz, $CDCl_3$):** δ (ppm) 7.34–7.10 (m, 20 H), 4.86, 4.71 (ABq, *J* = 11:2 Hz, 2 H), 4.81, 4.78 (ABq, *J* = 11:0 Hz, 2 H), 4.77, 4.46 (ABq, *J* = 11:0 Hz, 2 H), 4.69–4.66 (m, 1 H), 4.55, 4.41 (ABq, *J* = 11:9 Hz, 2 H), 4.03 (dd, *J* = 9:2; 5:7 Hz, 1 H), 3.64 (dd, *J* = 9:8; 2:7 Hz, 1 H), 3.54 (dd, *J* = 9:8; 6:0 Hz, 1 H), 3.51–3.42 (m, 2 H), 3.16–3.07 (m, 1 H); **^{13}C-NMR (126 MHz, $CDCl_3$):** δ (ppm) 154.8, 138.1, 137.8, 137.6, 136.8, 128.9, 128.7, 128.5, 128.4, 128.4, 128.2, 128.1, 127.9, 127.9, 127.8, 127.8, 127.8, 83.1, 79.6, 79.4, 75.7, 75.1, 74.8, 73.1, 69.1, 54.6, 51.0; **IR (film)** 3316, 3087, 3062, 3031, 2955, 2925, 2855, 1951, 1875, 1810, 1737, 1667, 1590, 1554, 1496, 1454, 1399, 1364, 1332, 1312, 1277, 1248, 1208, 1187, 1085, 1028; **HRMS (ESI-TOF)** m/z calcd for $C_{35}H_{38}N_5O_4$: 592.2924 found: 592.2925

2 Crystallographic data of compounds 3a and 3e

Colorless crystals suitable for X-ray structural analysis were obtained by slow evaporation of hexane–diethyl ether solution of **3a** and heptane-diethyl ether solution of **3e**. Crystal data were obtained on a Bruker APEX II CCD detector employing graphite monochromated Cu-Kα radiation (λ = 1:541 78 Å) at 296(2) K and operating in the φ - ω scan mode. The structure was solved by direct methods SHELXS-2014[S2] and refined with full-matrix least-squares calculations on F^2 using SHELX-2014.[S2] All non-hydrogen atoms were refined anisotropically. The hydrogen atom positions were geometrically

idealized and allowed to ride on their parent atoms. Crystallographic data for **3a** and **3e** have been deposited at the Cambridge Crystallographic Data Centre (deposition no. CCDC 1878740). Copies of these data can be obtained free of charge *via* www.ccdc.cam.ac.uk/conts/retrieving.html or from the Cambridge Crystallographic Data Centre, 12, Union Road, Cambridge CB21EZ, UK [fax: (+44) 1223-336-033; or email: deposit@ccdc.cam.ac.uk].

2.1 Crystal structure of compound 3a

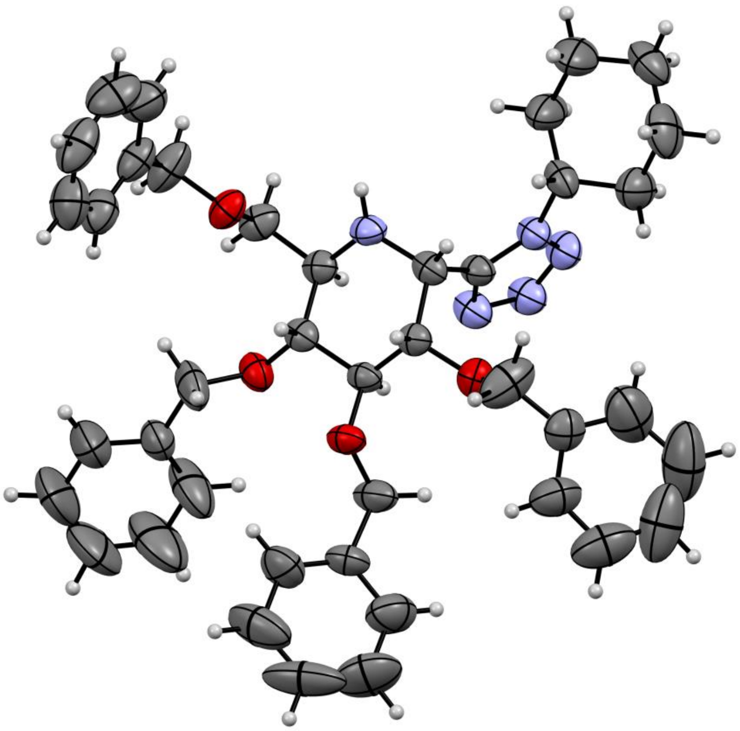

Figure S1: ORTEP plot of **3a**, represented by thermal ellipsoids shown at the 35% probability level.

Table S1: Summary ofthe crystal parameters for **3e**

Chemical formula	$C_{41}H_{47}N_5O_4$
Formula weigh	673.83 g·mol^{-1}
CCDC number	2001373
Crystal appearance	cubic, colourless
Crystal system	monoclinic

Space group	P 2_1
a	11.7353(5) Å
b	10.8296(4) Å
c	15.1159(6) Å
α	90°
β	93:981(3)°
γ	90°
volume	1916.42(13) $Å^3$
Z	2
R-factor	5.77%

2.2 Crystal structure of compound 3e

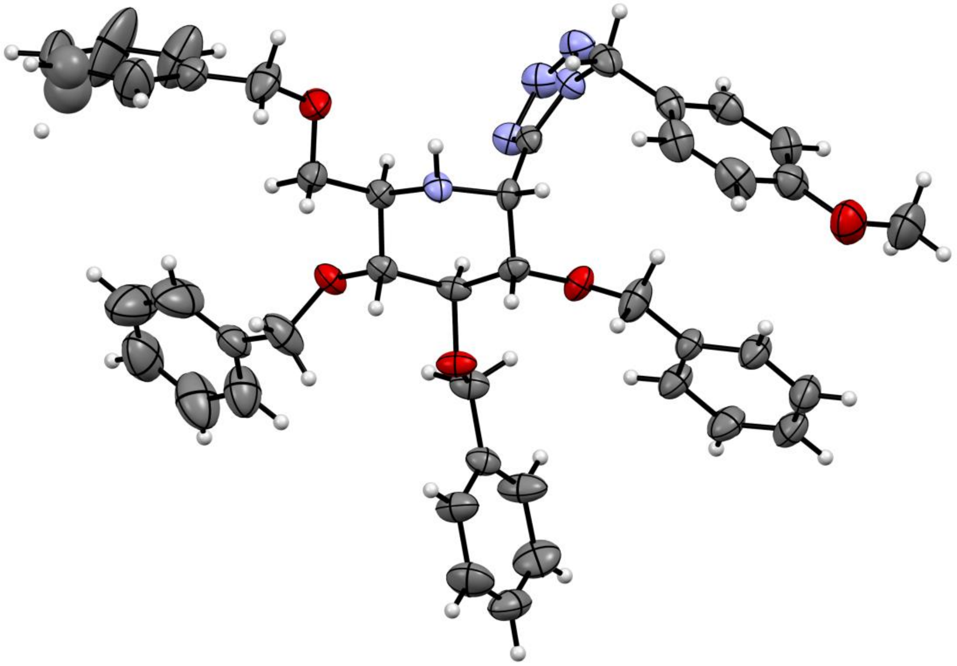

Figure S2: ORTEP plot of **3e**, represented by thermal ellipsoids shown at the 35% probability level.

Table S2: Summary of the crystal parameters for **3a**

Chemical formula	$C_{43}H_{45}N_5O_5$
Formula weigh	711:84 g·mol^{-1}
CCDC number	2001372
Crystal appearance	plate, yellow-colourless
Crystal system	monoclinic
Space group	P 2_1

a	5.8938(18) Å
b	19.634(6) Å
c	16.520(5) Å
α	90°
β	92.037(17)°
γ	90°
volume	1910.5(10) $Å^3$
Z	2
R-factor	4.01%

3 Analysis of circular dichroism spectra of compounds 5a and 2-epi-5a

As stated in the main text, we recorded an ECD spectrum of compounds **5a** and **2-epi-5a** and compared it with the simulated spectra, generated for both possible diastereomers (2-(*R*) and 2-(*S*)) using computational chemistry software. The comparison of these results can be seen in Figure S3. The ECD spectra were acquired at room temperature in CH_3CN (for UV-Spectroscopy, Fluka) on a Jasco J-815 spectropolarimeter and were collected with 0.2 nm/step and an integration time of 0.5 s over the range 195 nm to 400 nm. UV–vis spectra were measured on a Jasco V-670 spectrophotometer in CH_3CN. The conformational search for the calculations was made using the CONFLEX software. Resulting sets of conformers were further optimized using the Gaussian 09 software[S3], using B3LYP functional and 6-31G** basis set. Optimized structures up to 3 kcal were used to calculate ECD spectra in Gaussian 16 software[S4], using B3LYP functional and TZVP basis set, including PCM solvent model for acetonitrile. Simulated spectra were plotted with original Tesliper software[1], using a gaussian function with 0.15 eV line width and hypsochromically shifted by 15 nm.

[1] Source code available under an open software license at https://github.com/Mishioo/tesliper.

Unfortunately, the significant lability of the structures in question does not allow for accurate simulation in an acceptable time limit. This is particularly visible in the 180 nm to 200 nm range, with the strongest bands most likely coming from the —OBn groups. On the other hand, the bands in range 220 nm to 300 nm, shown in charts D, F, and H are not well developed and therefore hard to analyse. In conclusion, these experiments did not help to determine the absolute configuration of compounds **5a** and **2-epi-5a**, as there is no clear correspondence in simulated and experimental spectra.

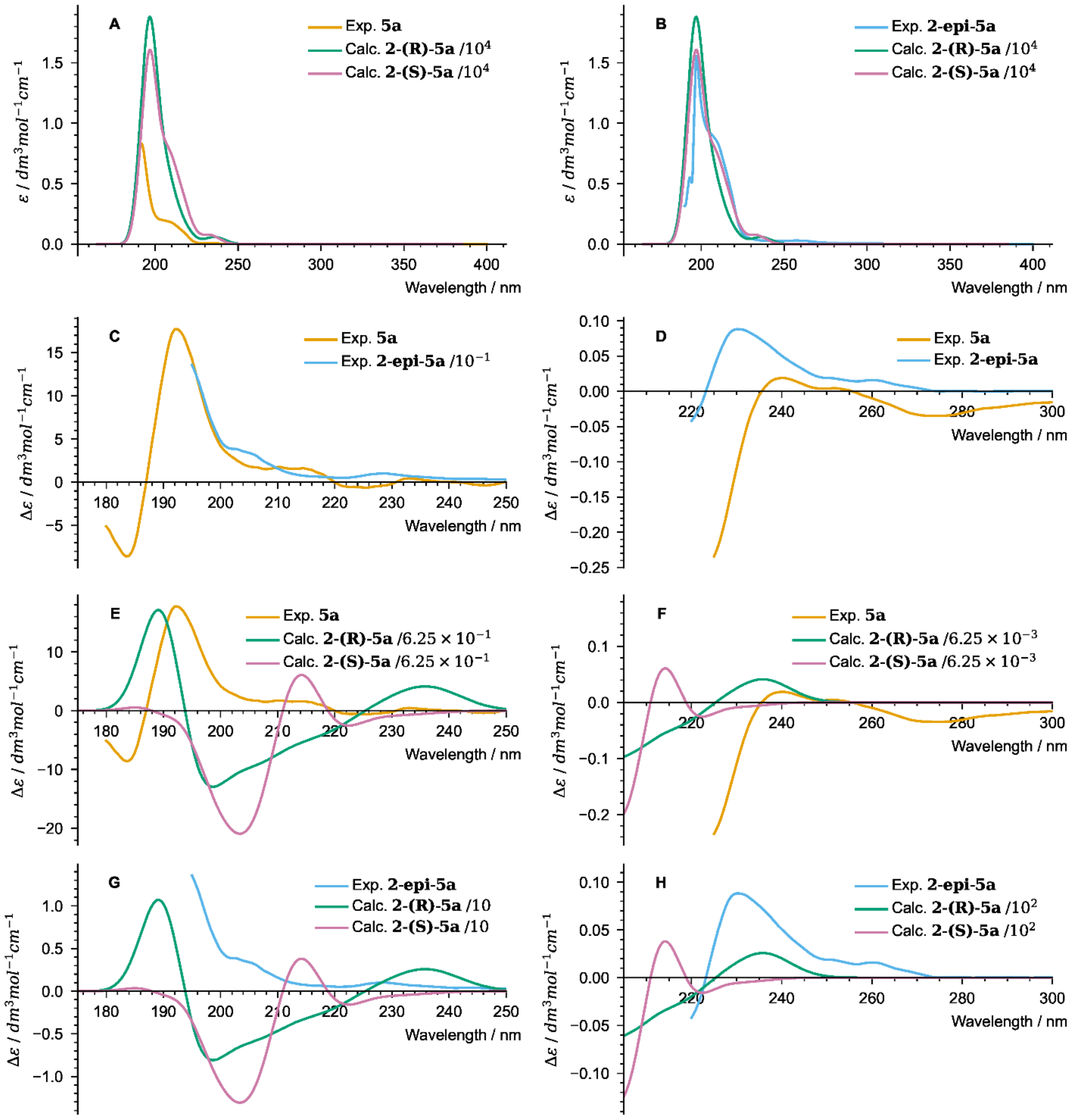

Figure S3: Comparison of experimental and simulated ECD spectra of **5a** and **2-epi-5a**. Intensity values were scaled for clarity by a factor given in the Figure.

4 NMR spectra

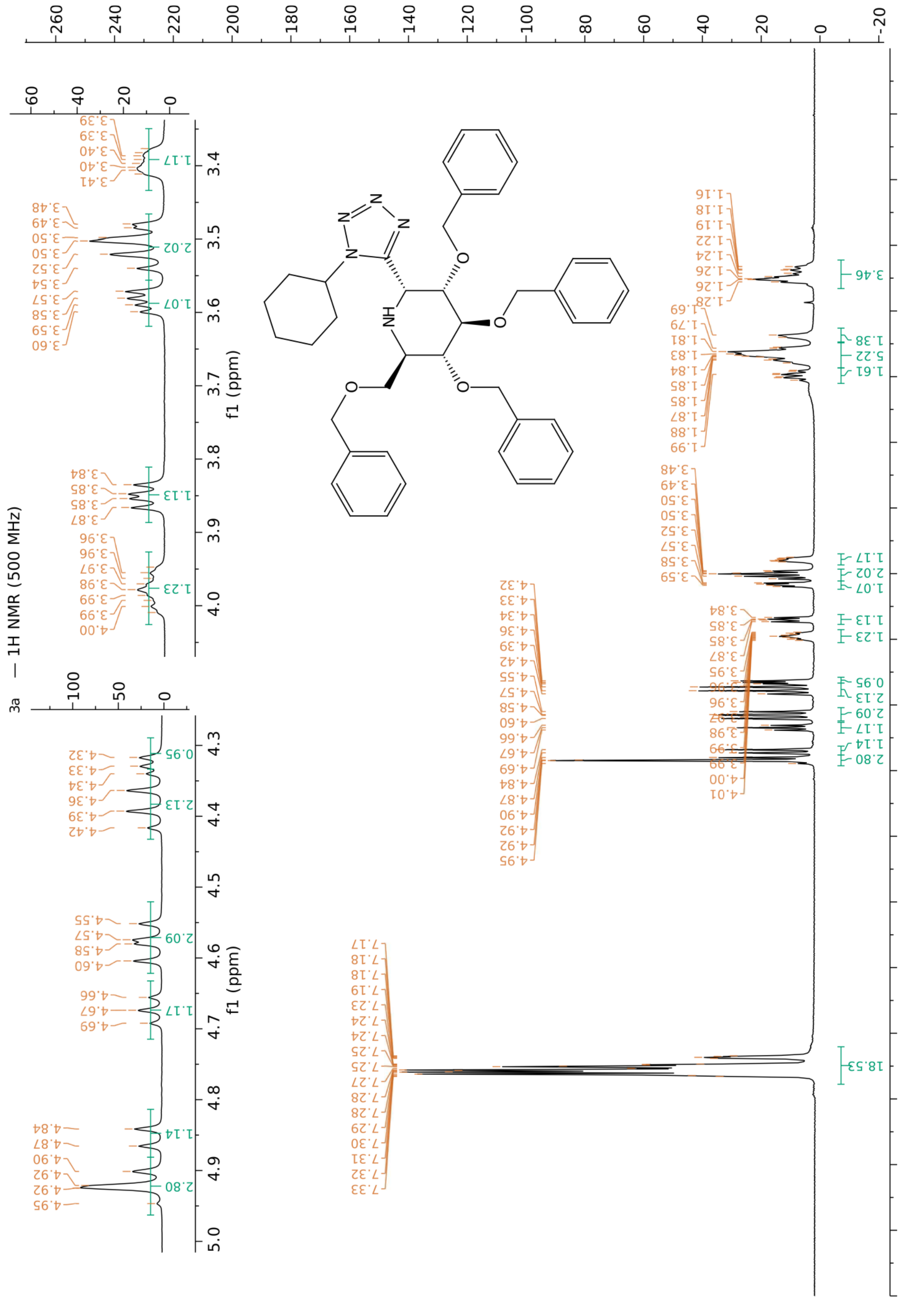
3a — 1H NMR (500 MHz)
f1 (ppm)
NH

3a — 13C NMR (126 MHz)

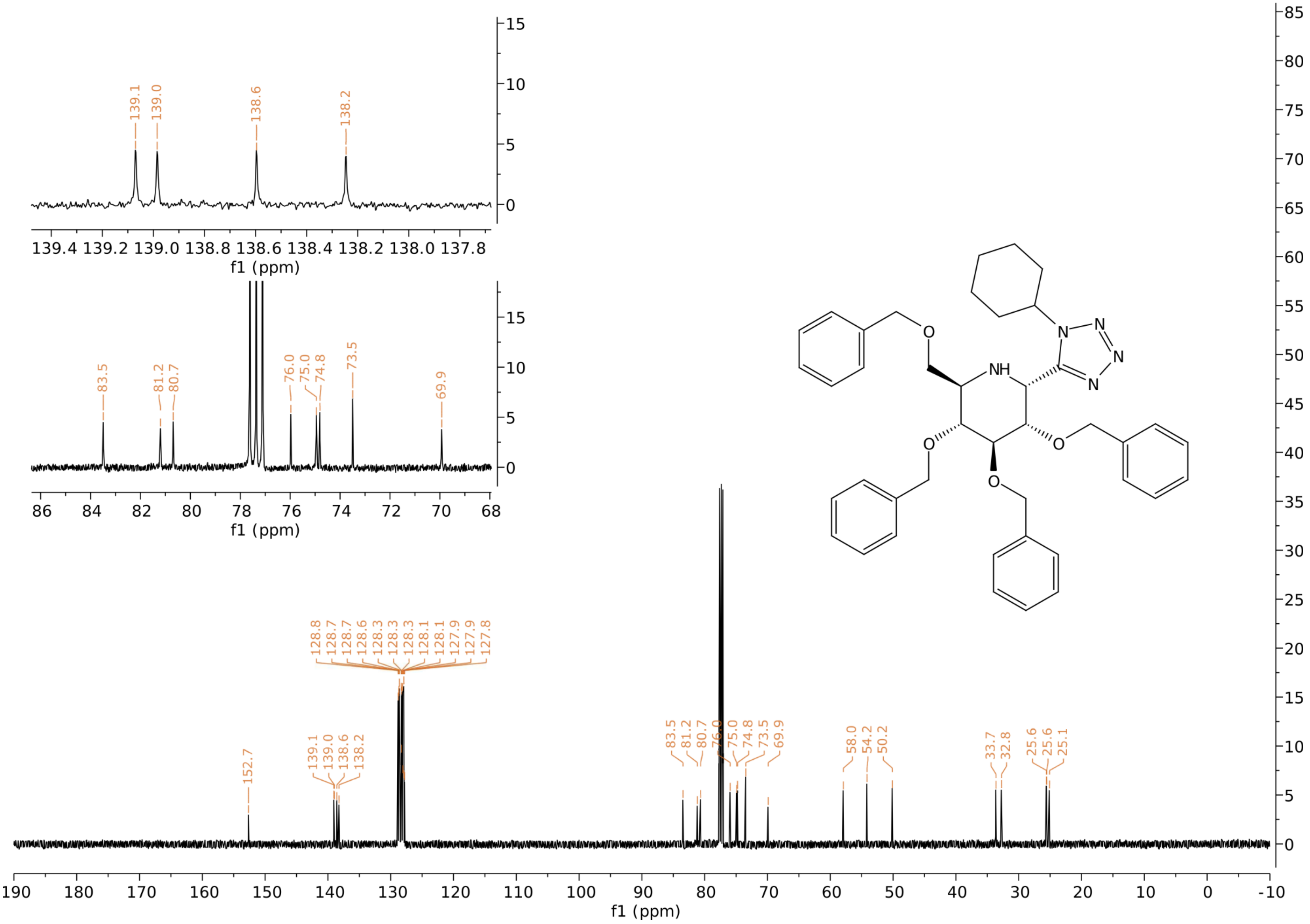

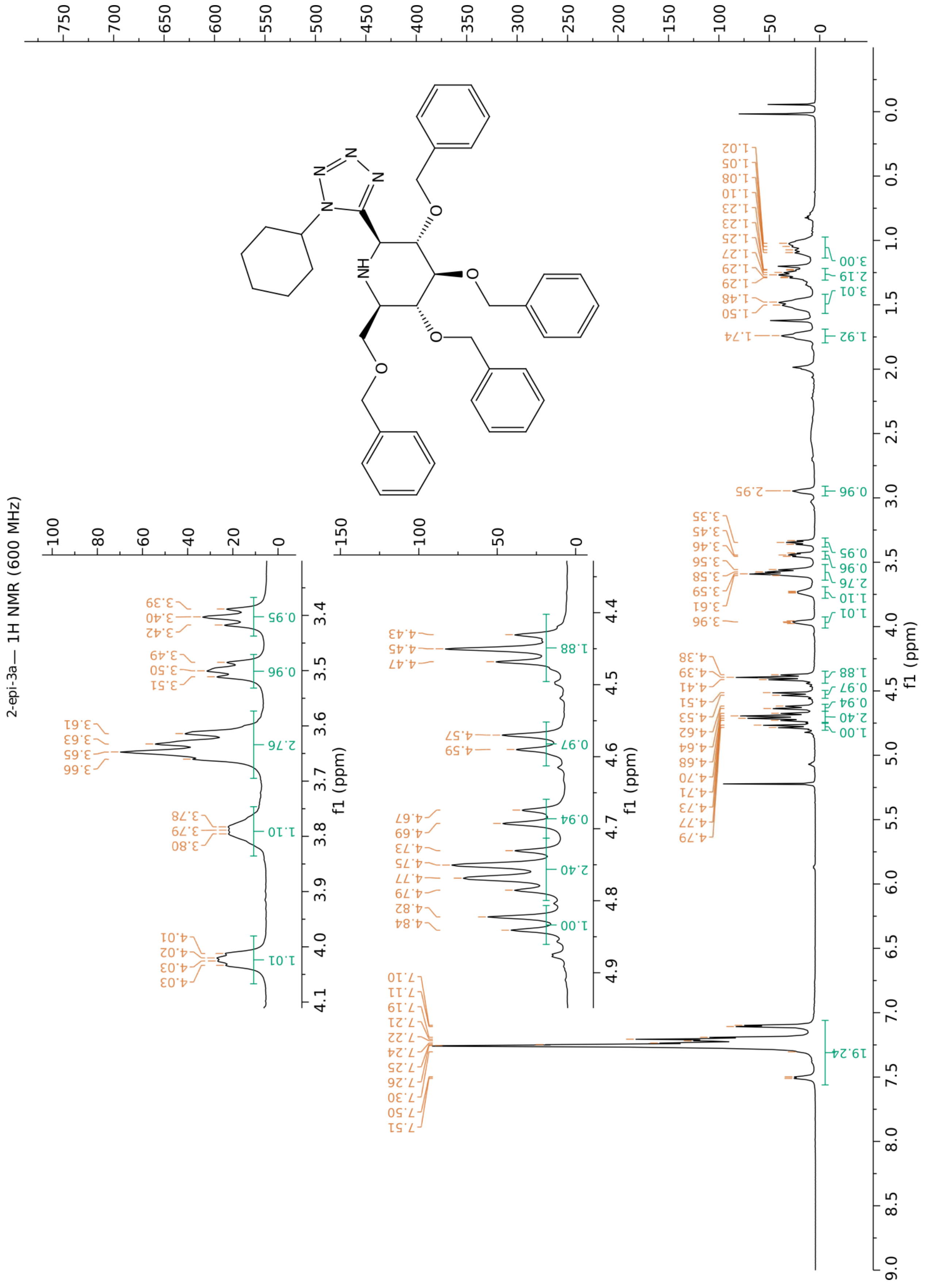
2-epi-3a— 1H NMR (600 MHz)
f1 (ppm)

2-epi-3a— 13C NMR (151 MHz)

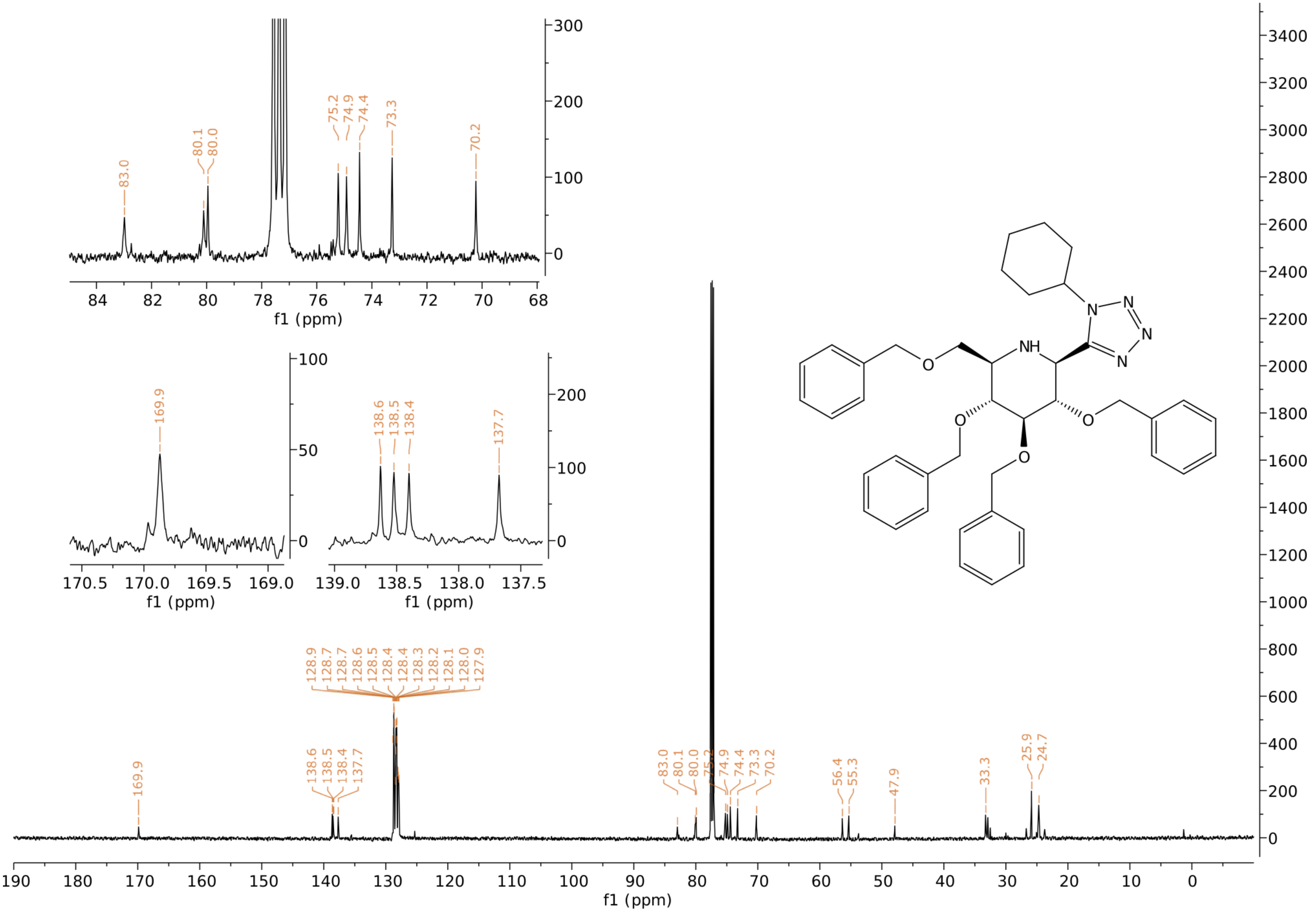

3b — 1H NMR (600 MHz)

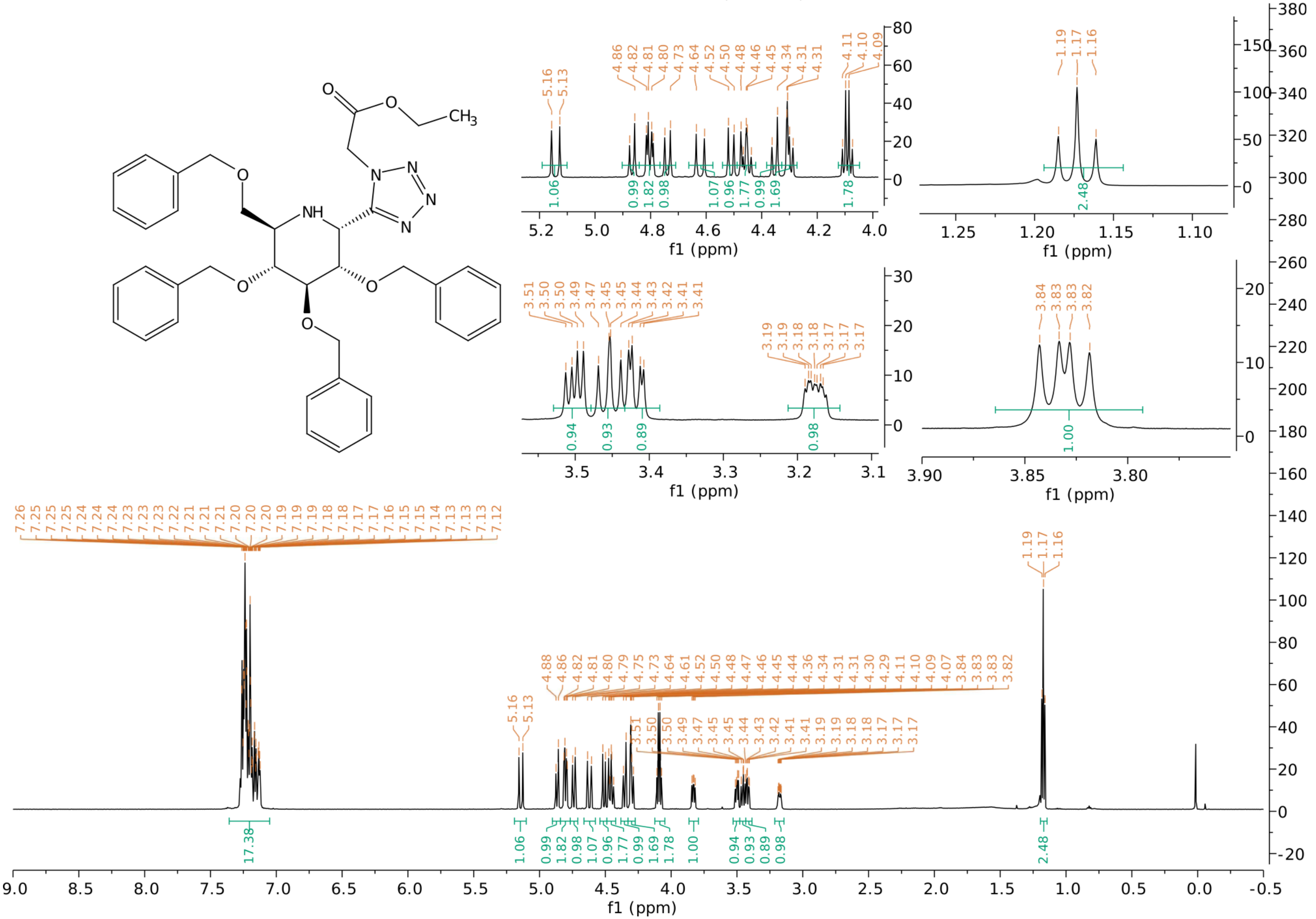

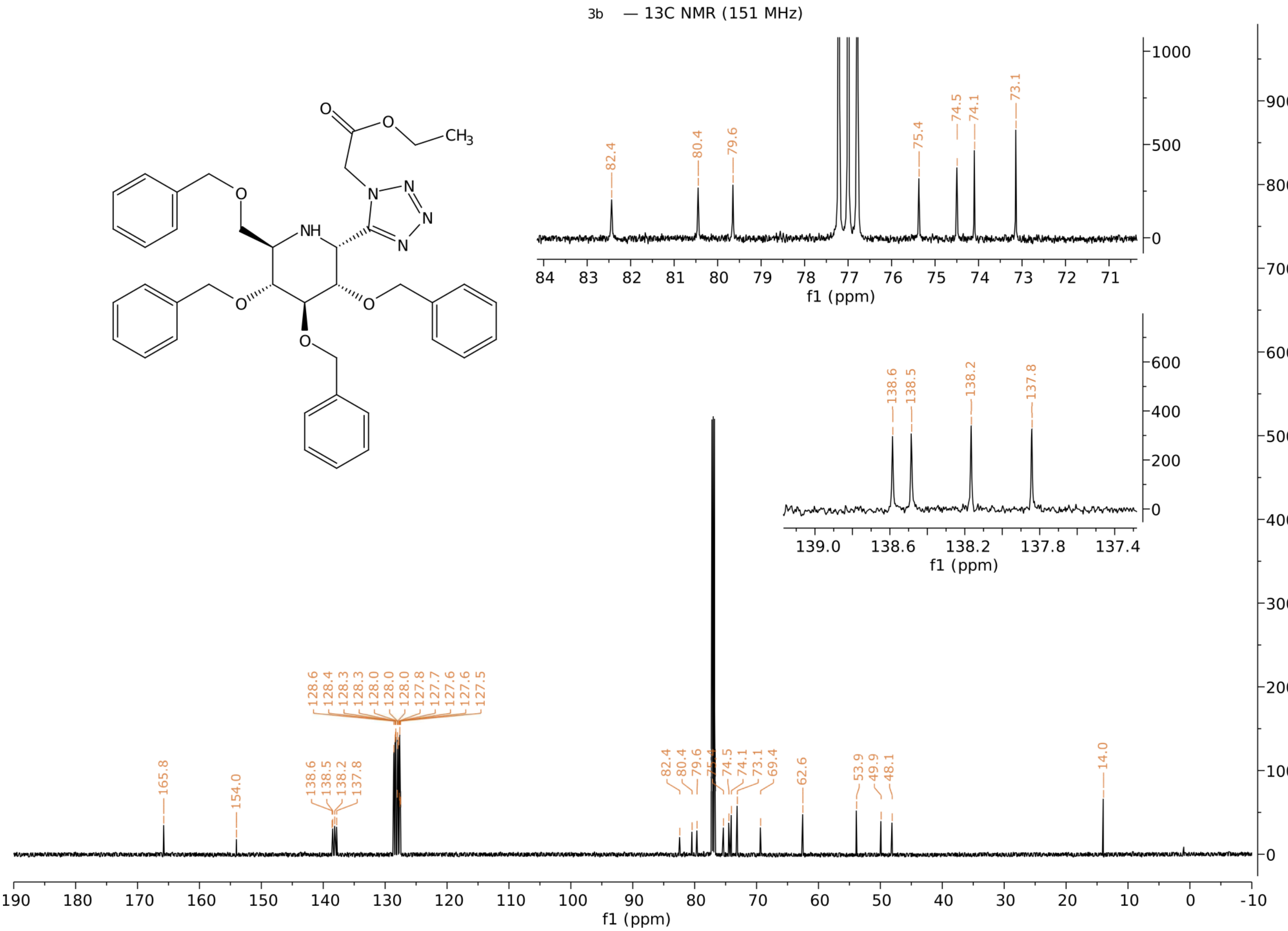
3b — 13C NMR (151 MHz)
NH
N
O
CH3
165.8
154.0
138.6
138.5
138.2
137.8
128.6
128.4
128.3
128.3
128.0
128.0
128.0
127.8
127.7
127.6
127.6
127.5
82.4
80.4
79.6
75.4
74.5
74.1
73.1
69.4
62.6
53.9
49.9
48.1
14.0
f1 (ppm)

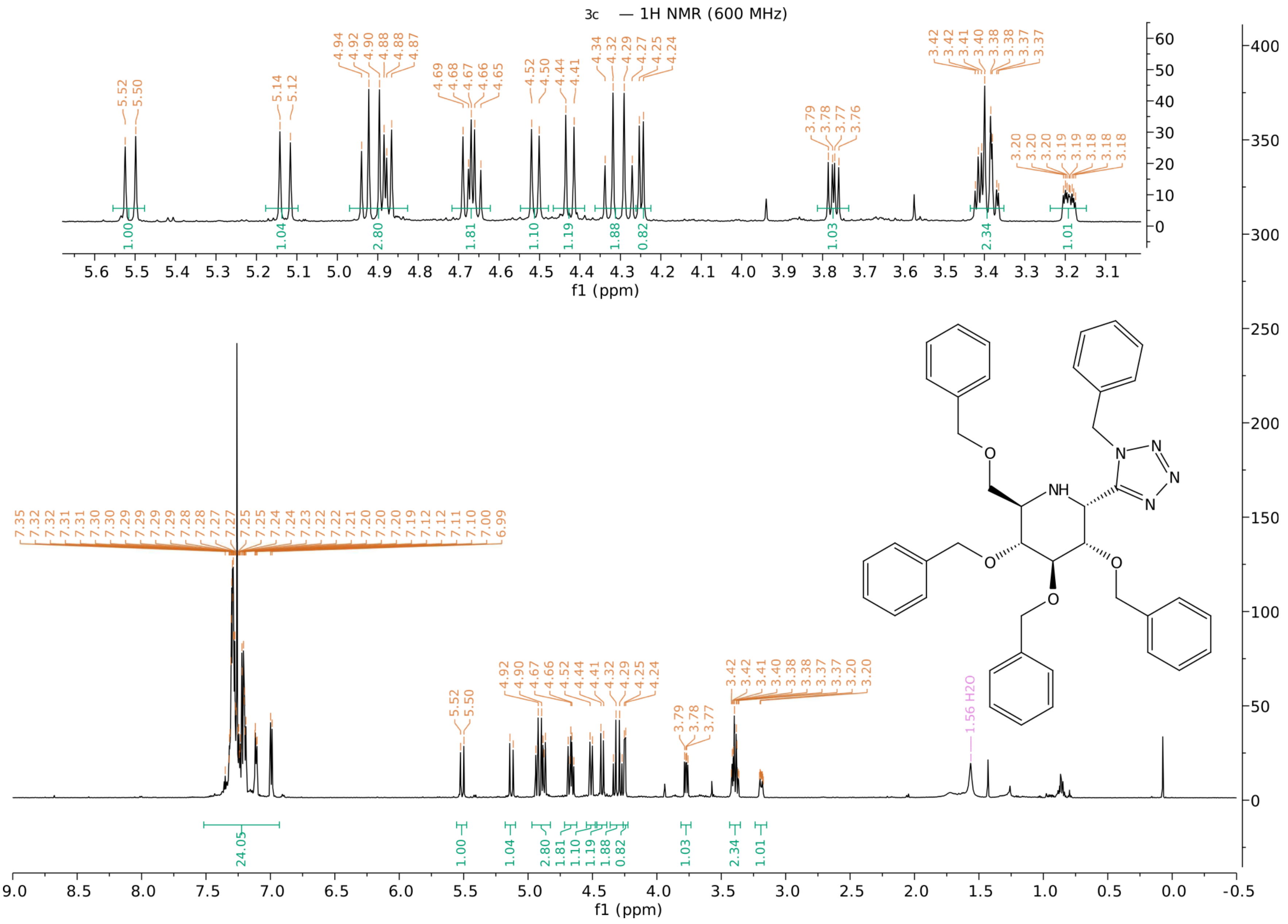
3c — 1H NMR (600 MHz)
f1 (ppm)
1.56 H2O
NH
24.05
1.00
1.04
2.80
1.81
1.10
1.19
1.88
0.82
1.03
2.34
1.01

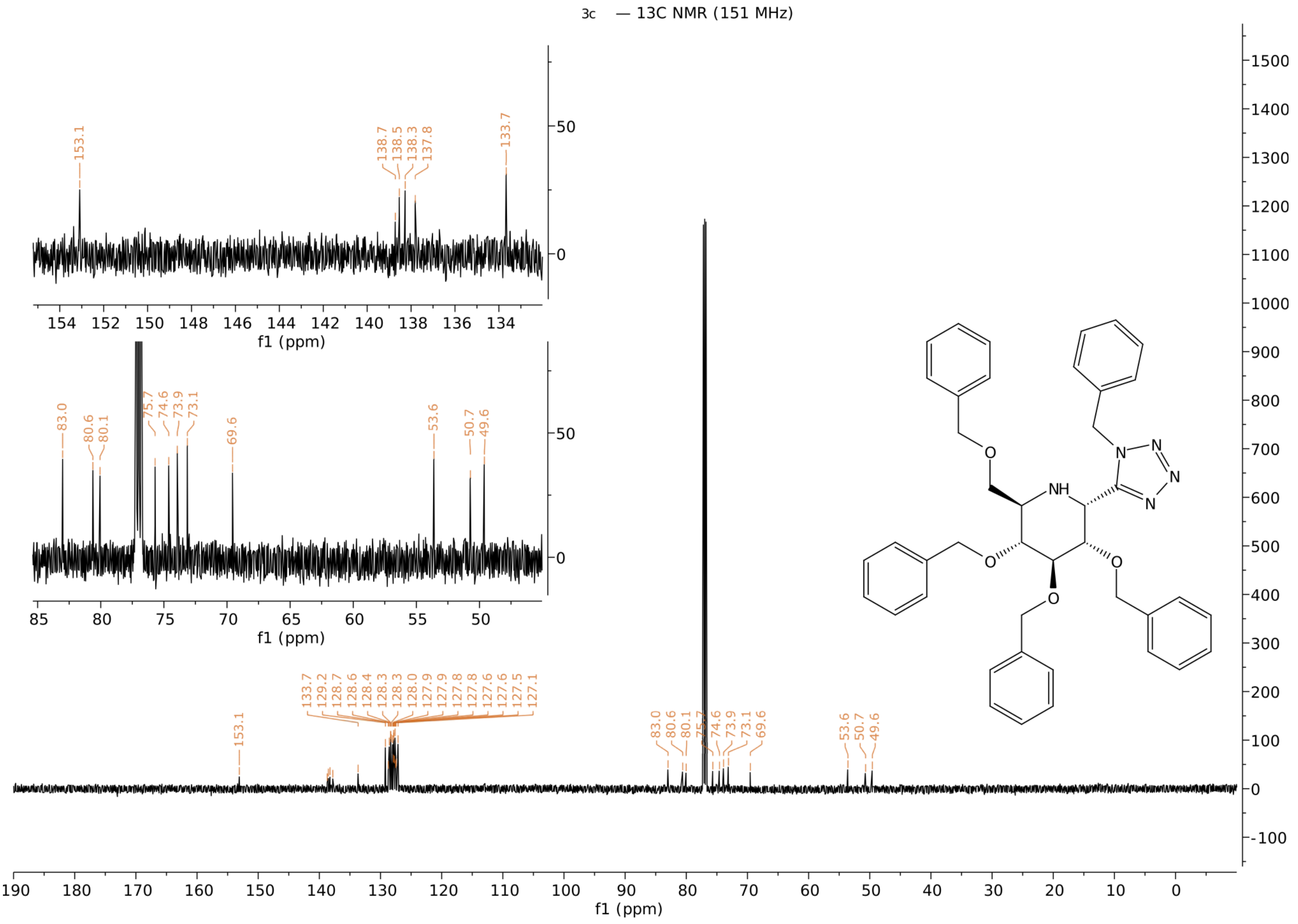
3c — 13C NMR (151 MHz)
153.1
138.7
138.5
138.3
137.8
133.7
83.0
80.6
80.1
75.7
74.6
73.9
73.1
69.6
53.6
50.7
49.6
133.7
129.2
128.7
128.6
128.4
128.3
128.3
128.0
127.9
127.9
127.8
127.8
127.6
127.6
127.5
127.1
NH
O
N
f1 (ppm)

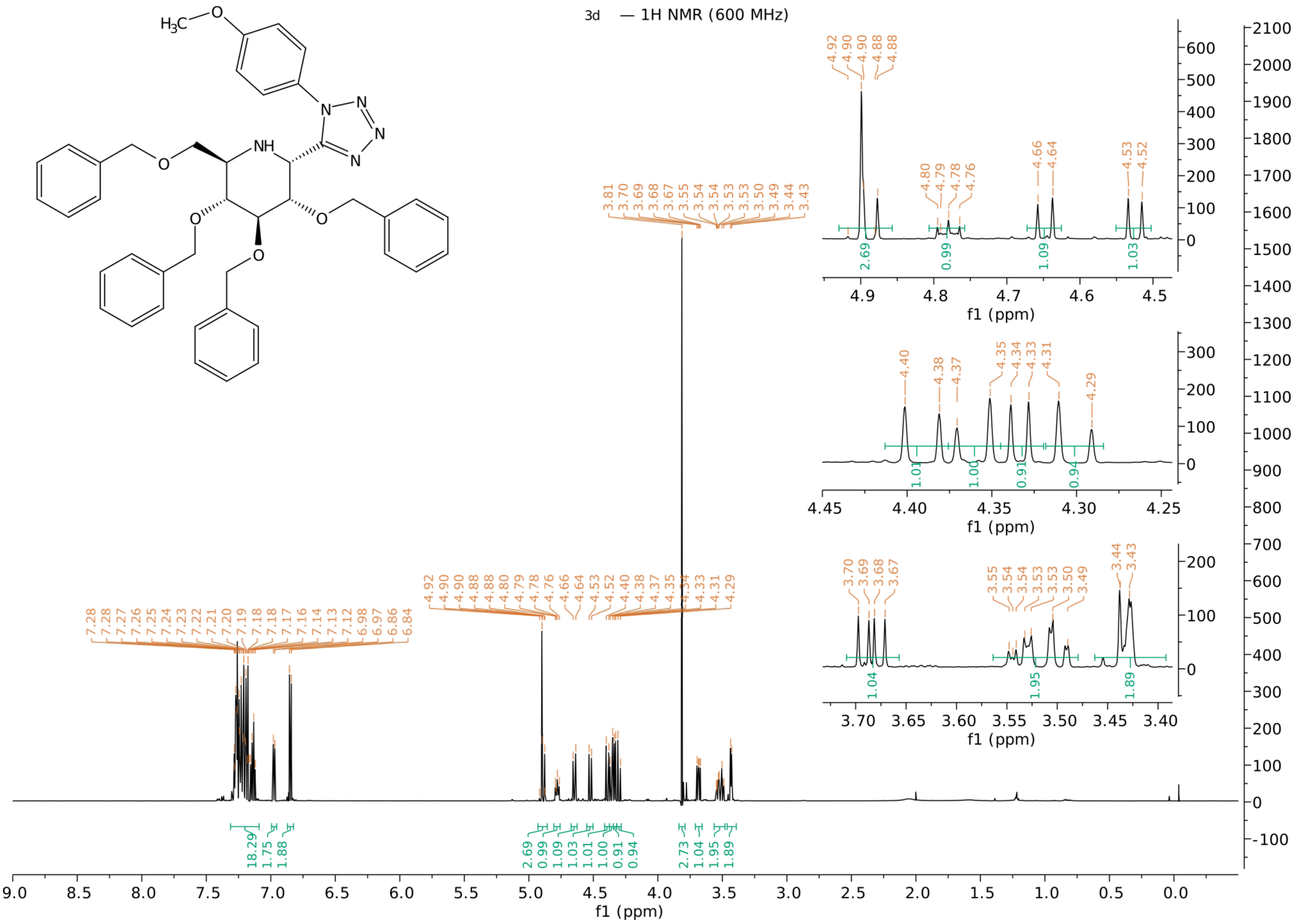
H3C
O
N
N
N
N
NH
O
O
O
O
3d — 1H NMR (600 MHz)
f1 (ppm)
f1 (ppm)
f1 (ppm)
f1 (ppm)

3d — 13C NMR (151 MHz)

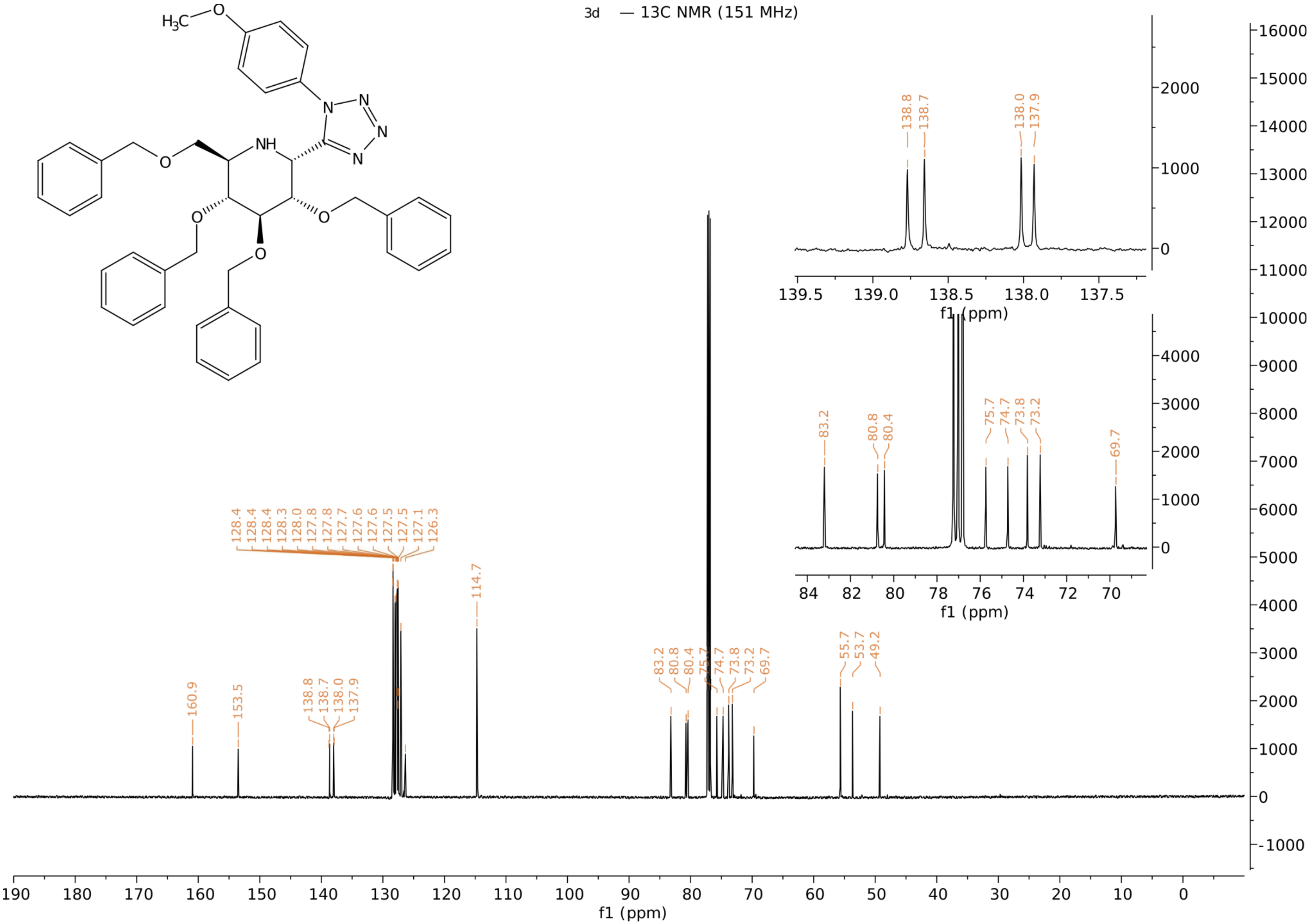

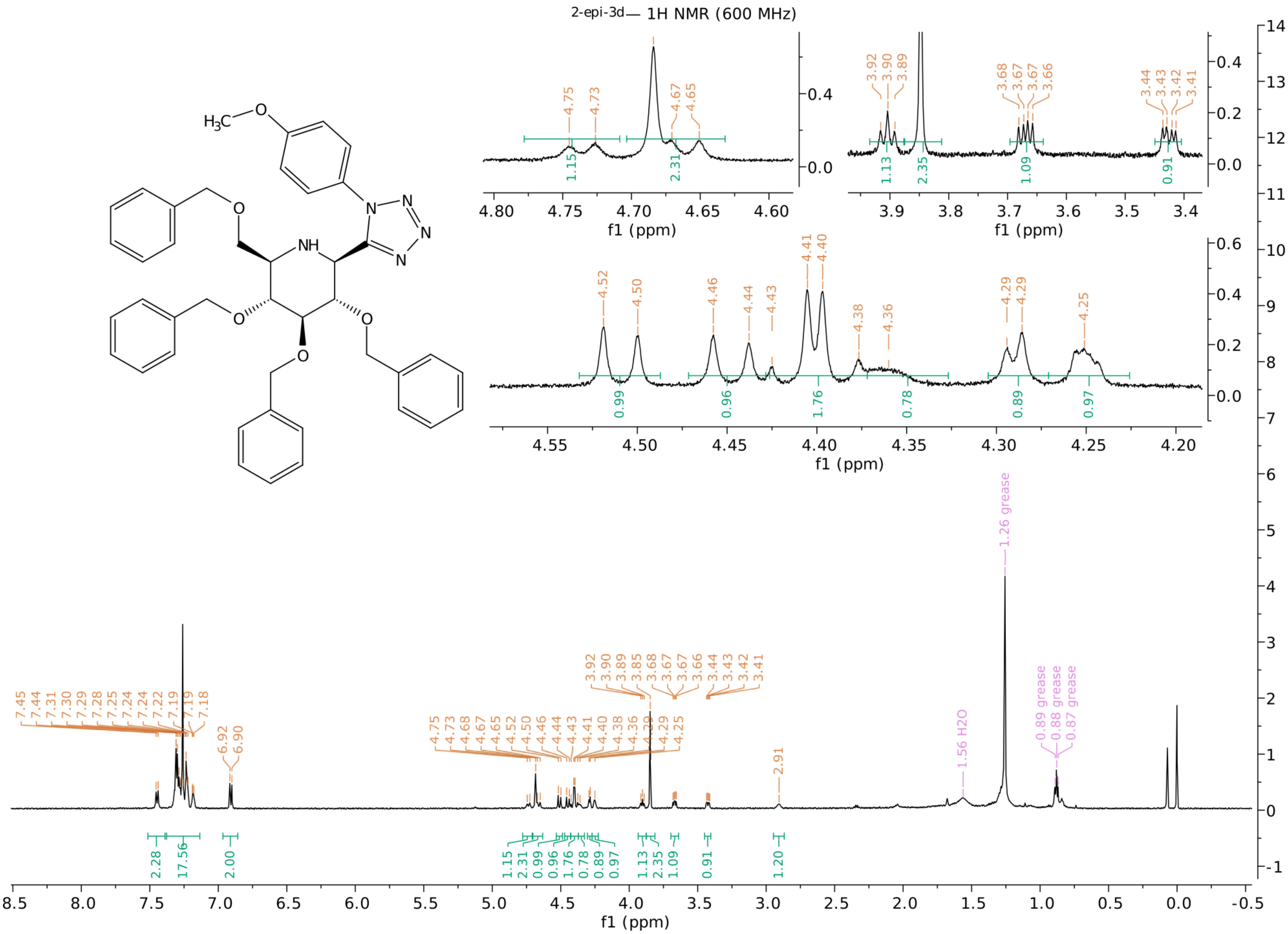
2-epi-3d— 1H NMR (600 MHz)
f1 (ppm)
1.26 grease
1.56 H2O
0.89 grease
0.88 grease
0.87 grease
2.91
7.45 7.44 7.31 7.30 7.29 7.28 7.25 7.24 7.24 7.22 7.19 7.19 7.18
6.92 6.90
4.75 4.73 4.68 4.67 4.65 4.52 4.50 4.46 4.44 4.43 4.41 4.40 4.38 4.36 4.29 4.29 4.25
3.92 3.90 3.89 3.85 3.68 3.67 3.67 3.66 3.44 3.43 3.42 3.41
2.28 17.56 2.00 1.15 2.31 0.99 0.96 1.76 0.78 0.89 0.97 1.13 2.35 1.09 0.91 1.20
NH
N
O
H_3C

2-epi-3d— 13C NMR (151 MHz)

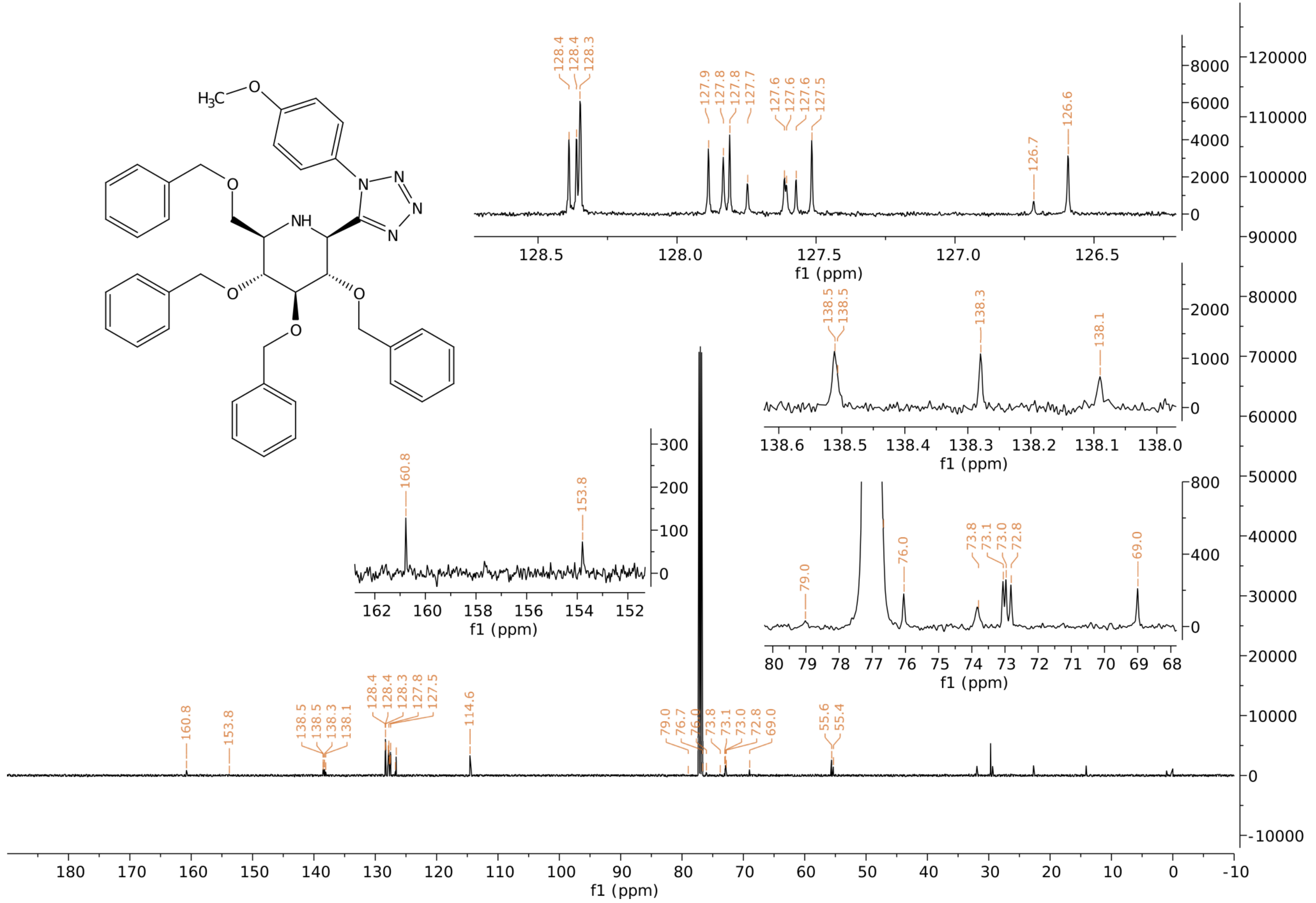

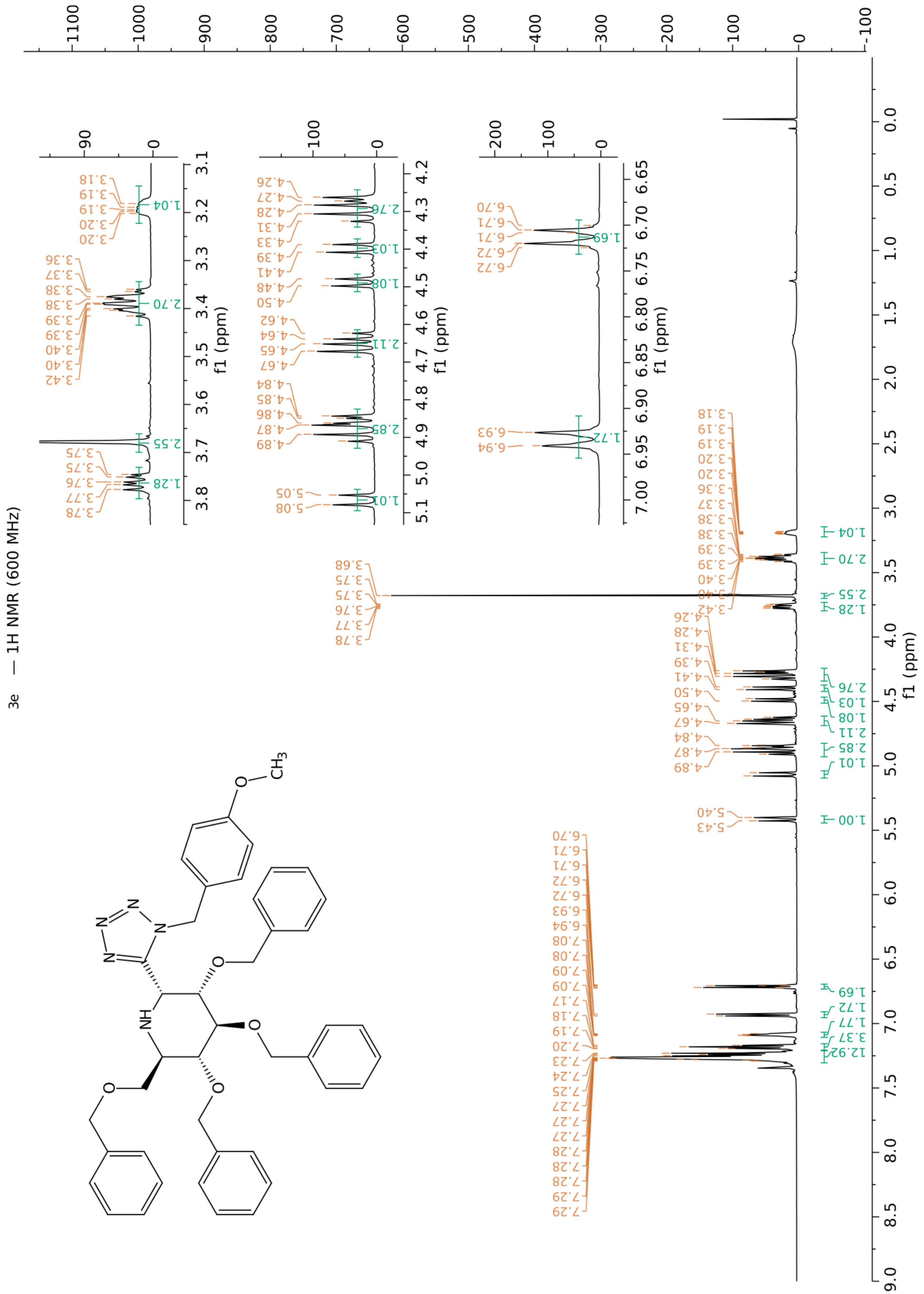
3e
— 1H NMR (600 MHz)
f1 (ppm)

3e — 13C NMR (151 MHz)

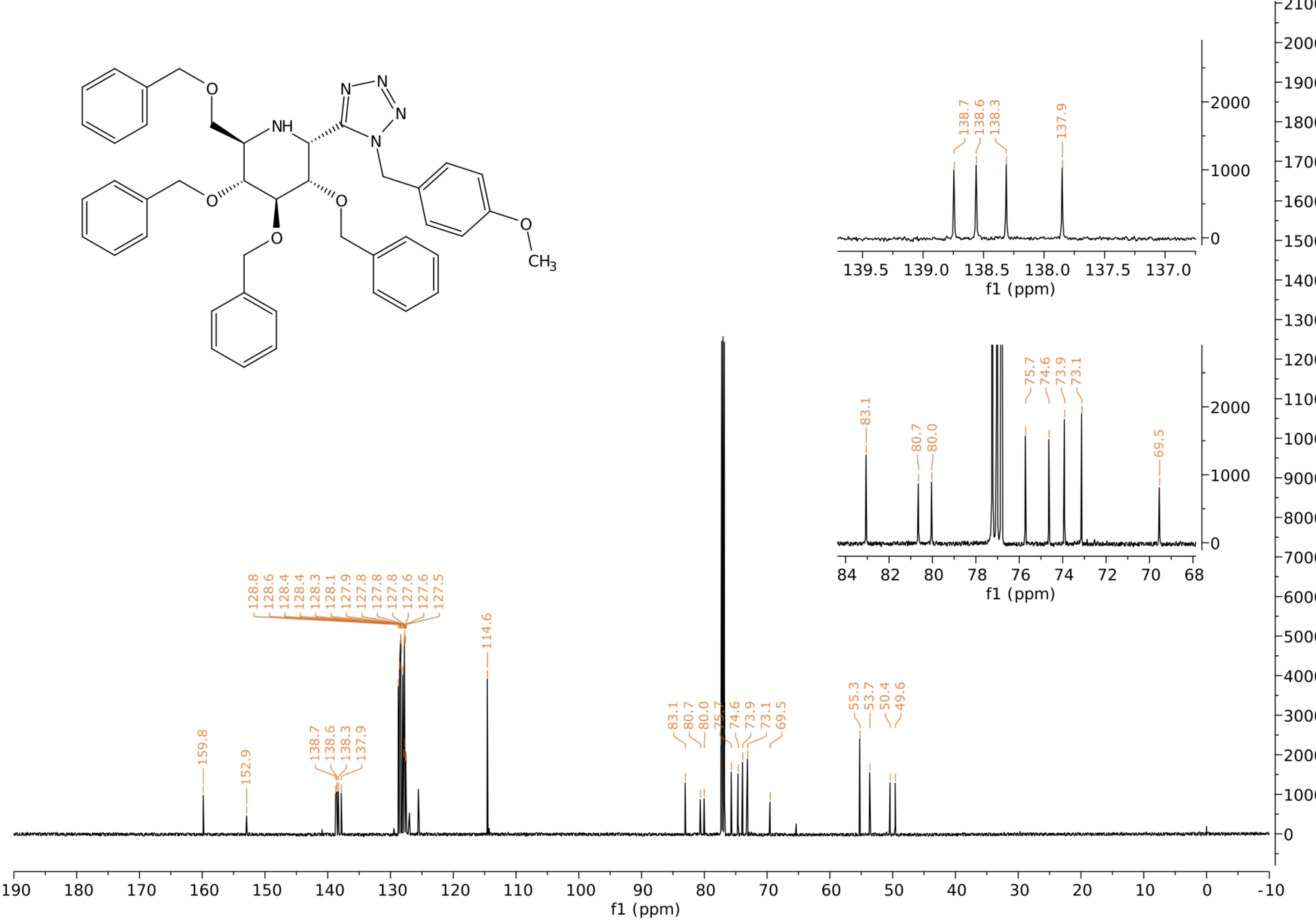

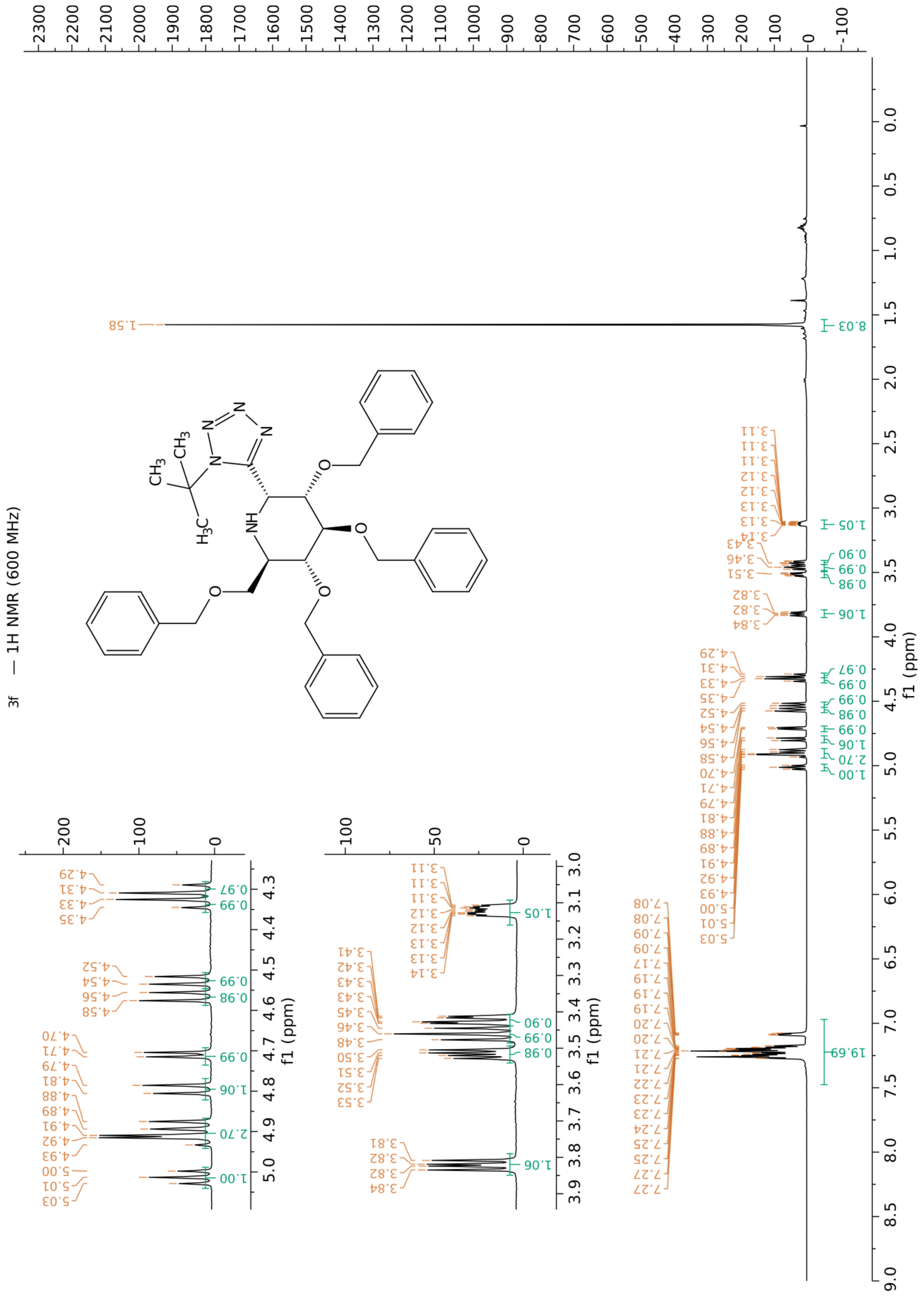
3f — 1H NMR (600 MHz)

3f — 13C NMR (151 MHz)

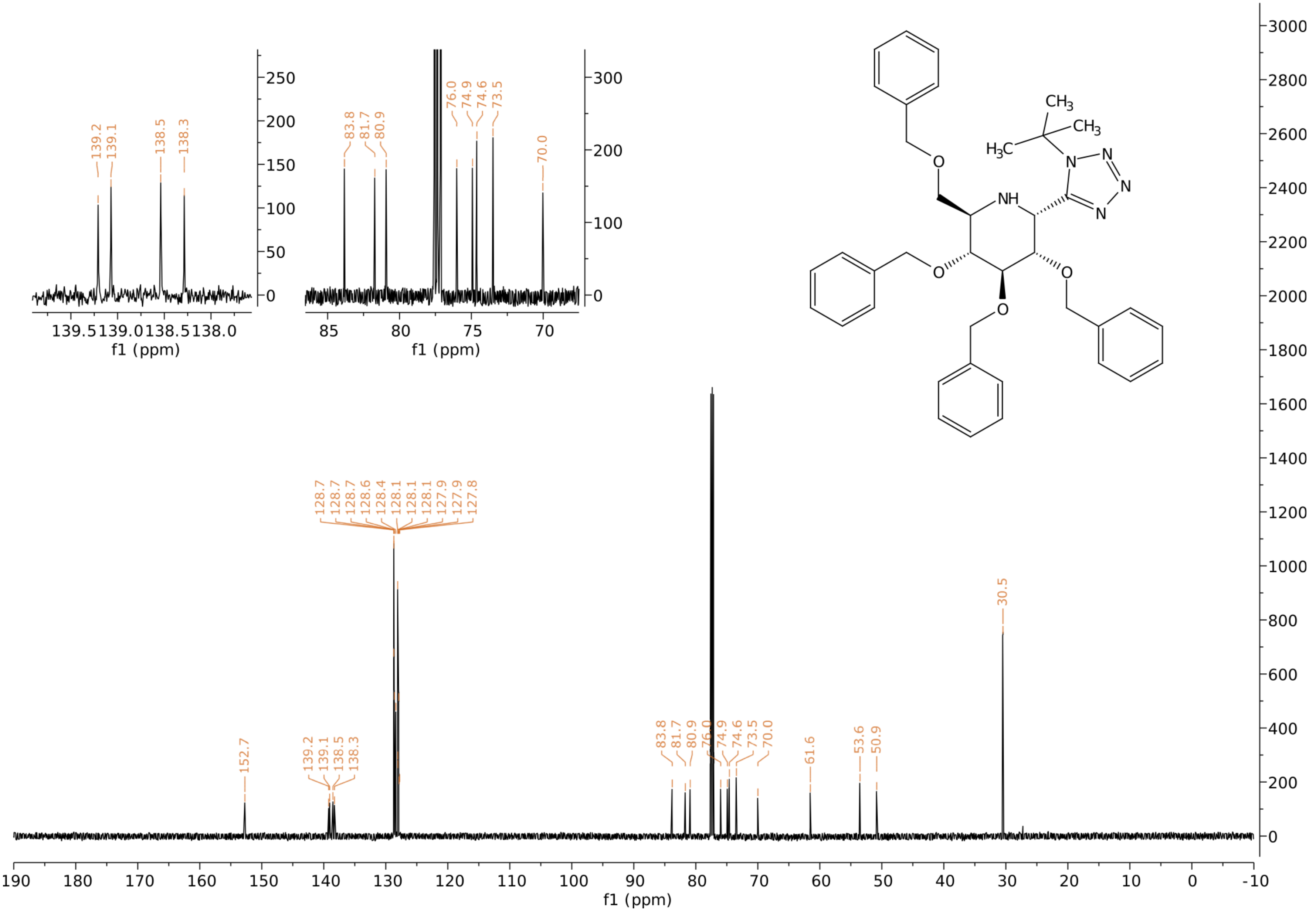

3g — 1H NMR (600 MHz)

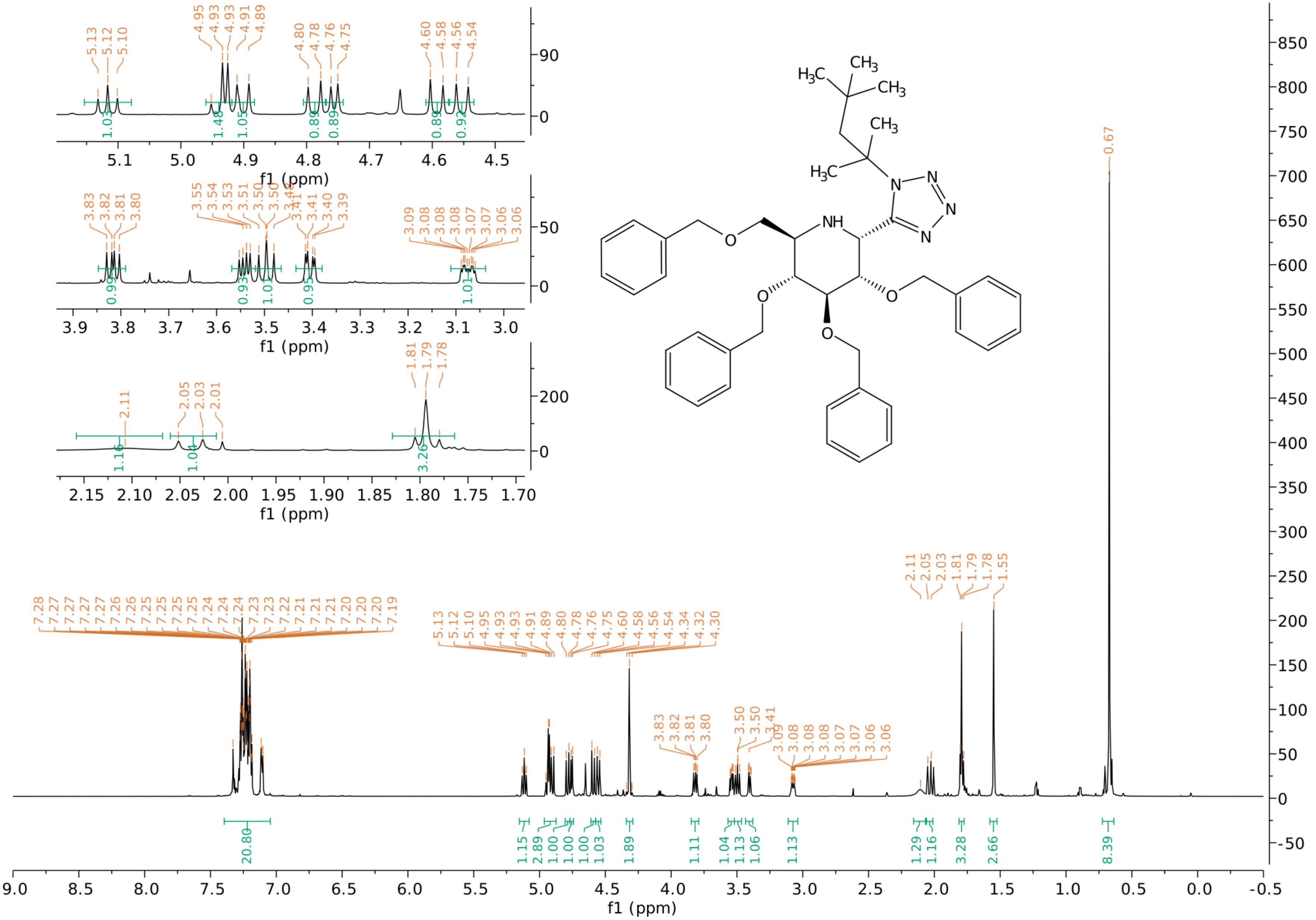

3g — 13C NMR (151 MHz)

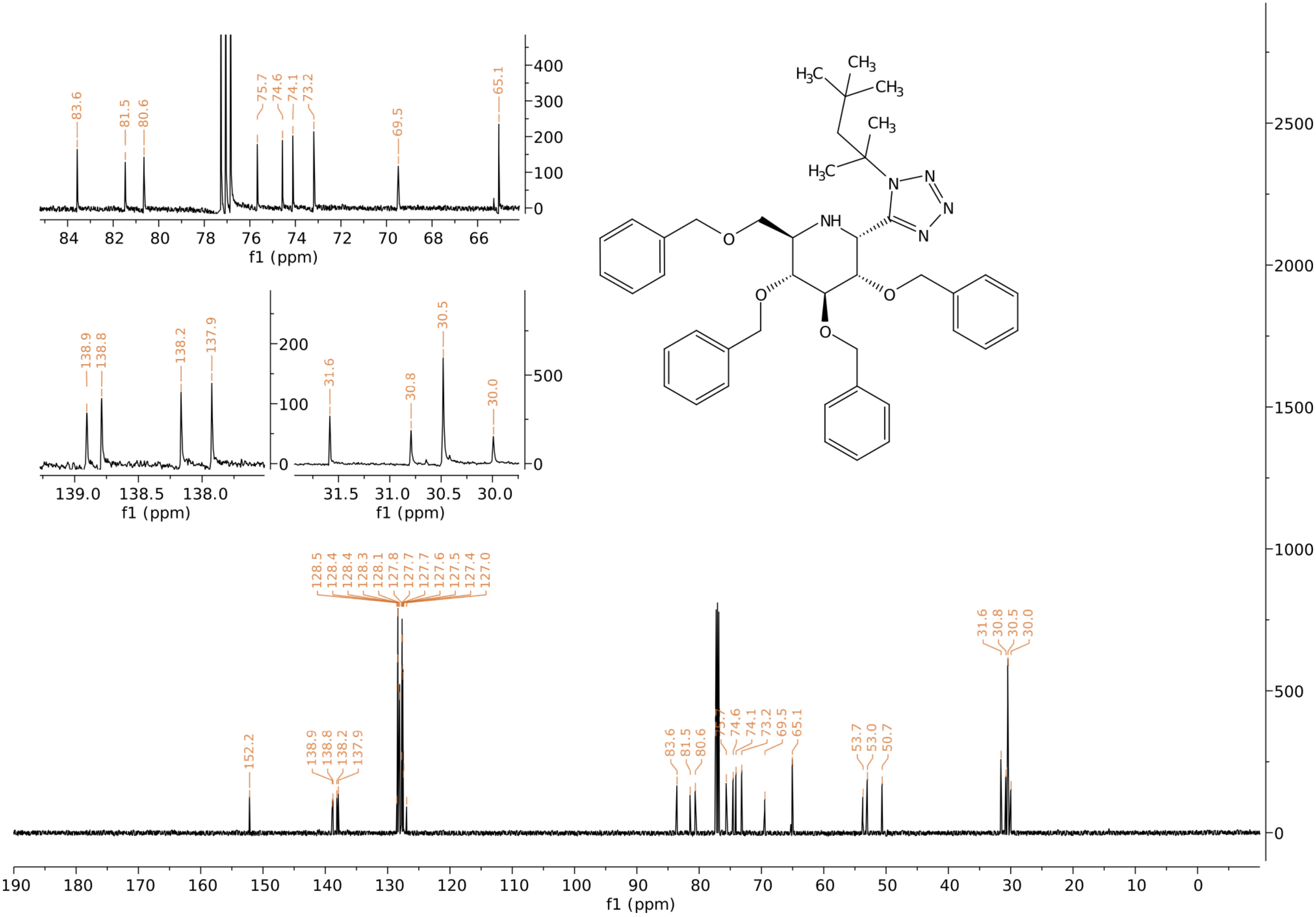

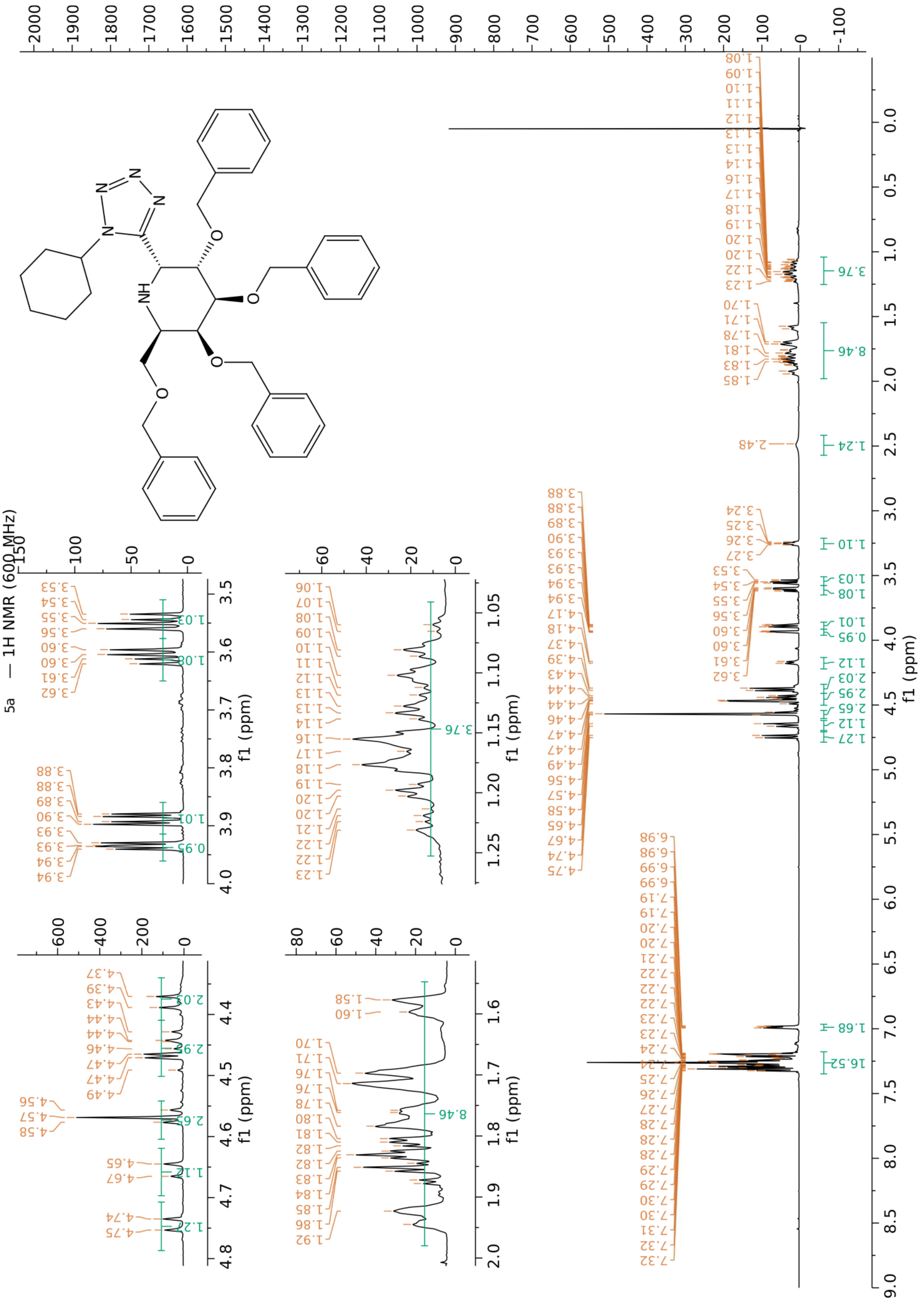
5a
— 1H NMR (600 MHz)
f1 (ppm)

2-epi-5a— 1H NMR (600 MHz)

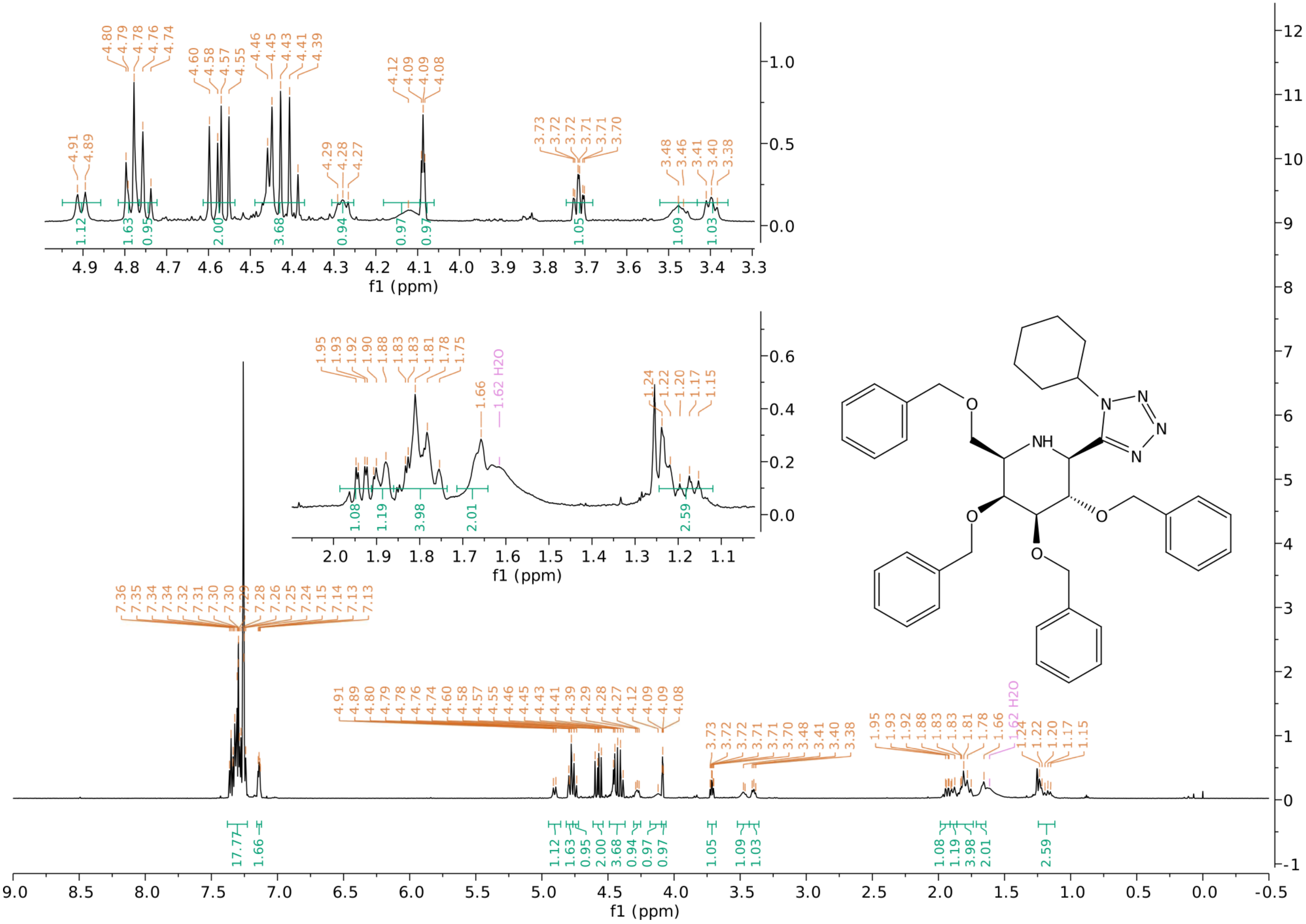

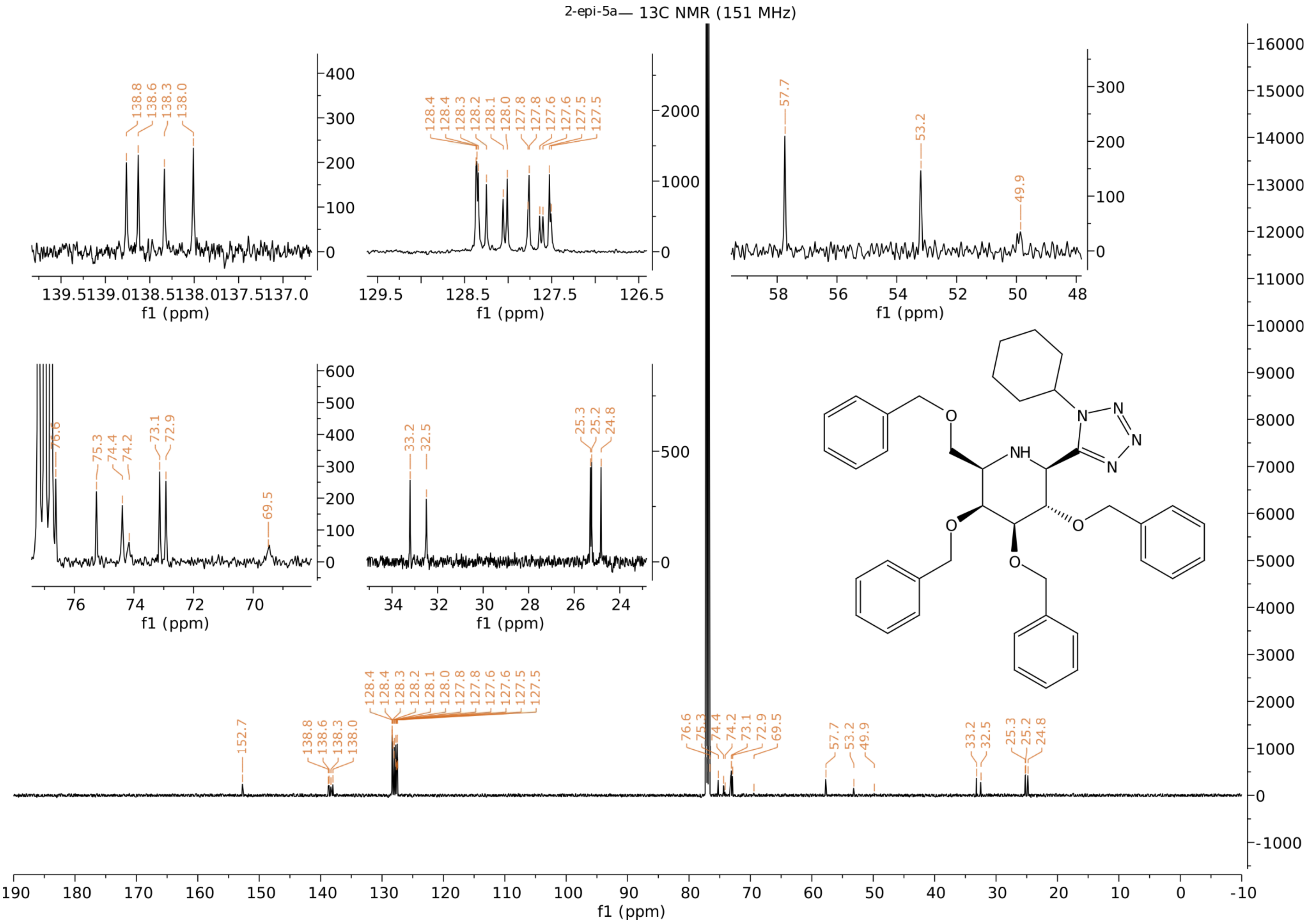
2-epi-5a— 13C NMR (151 MHz)
f1 (ppm)

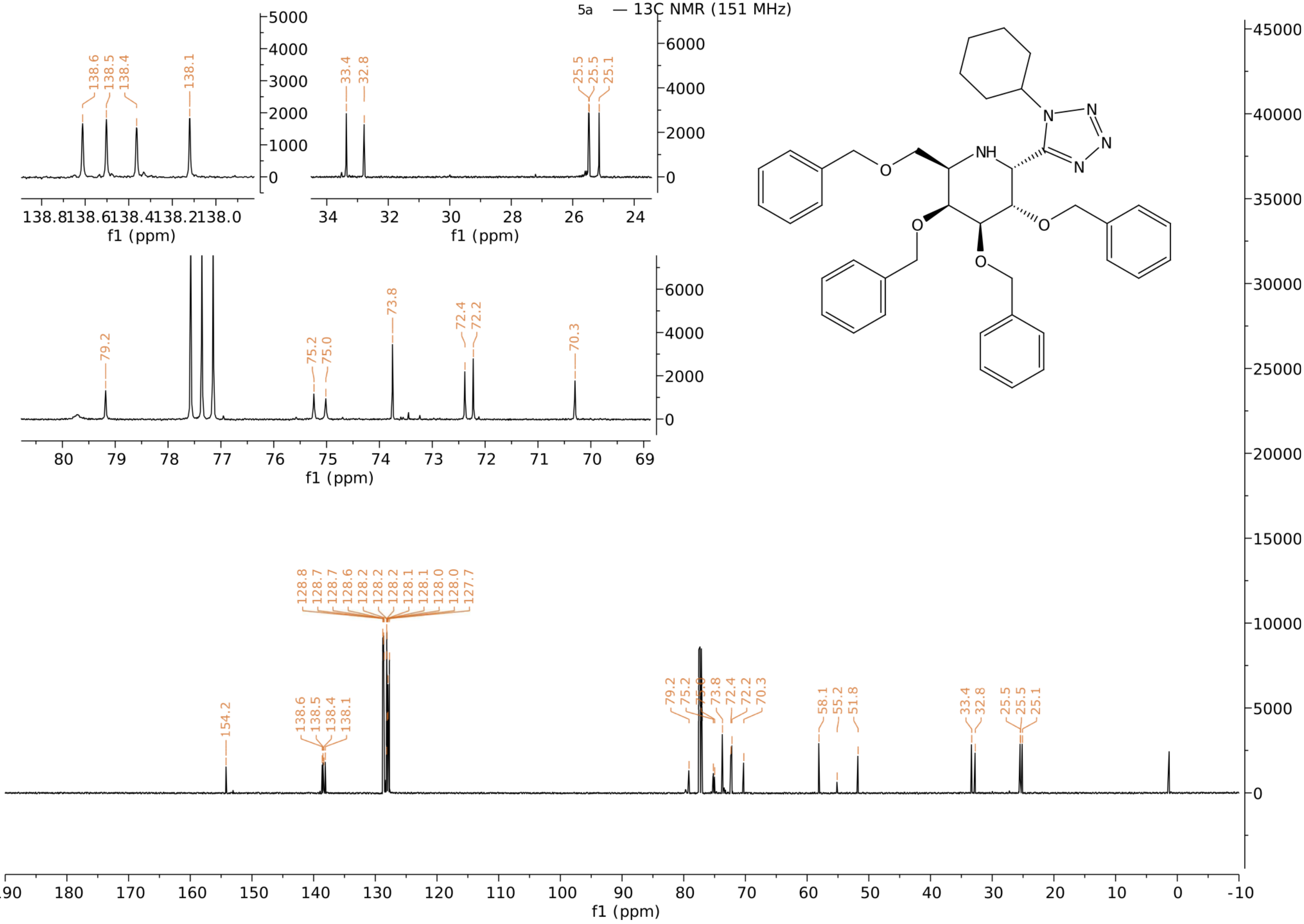
5a — 13C NMR (151 MHz)
154.2
138.6 138.5 138.4 138.1
128.8 128.7 128.7 128.6 128.2 128.2 128.2 128.1 128.1 128.0 128.0 127.7
79.2 75.2 75.0 73.8 72.4 72.2 70.3
58.1 55.2 51.8
33.4 32.8
25.5 25.5 25.1
f1 (ppm)
NH
O
N

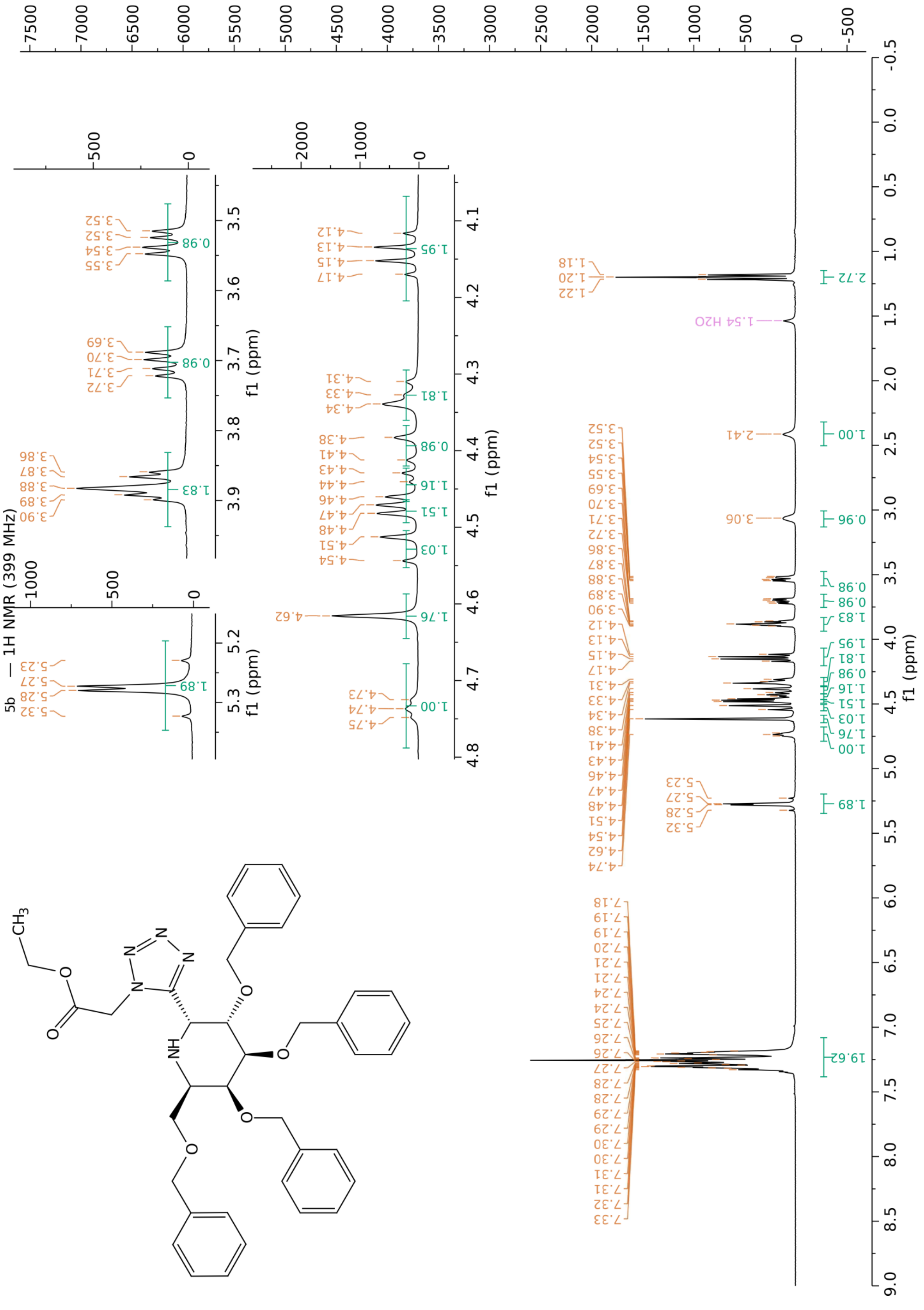
5b — 1H NMR (399 MHz)
f1 (ppm)
1.54 H2O
NH
CH3

5b — 13C NMR (126 MHz)

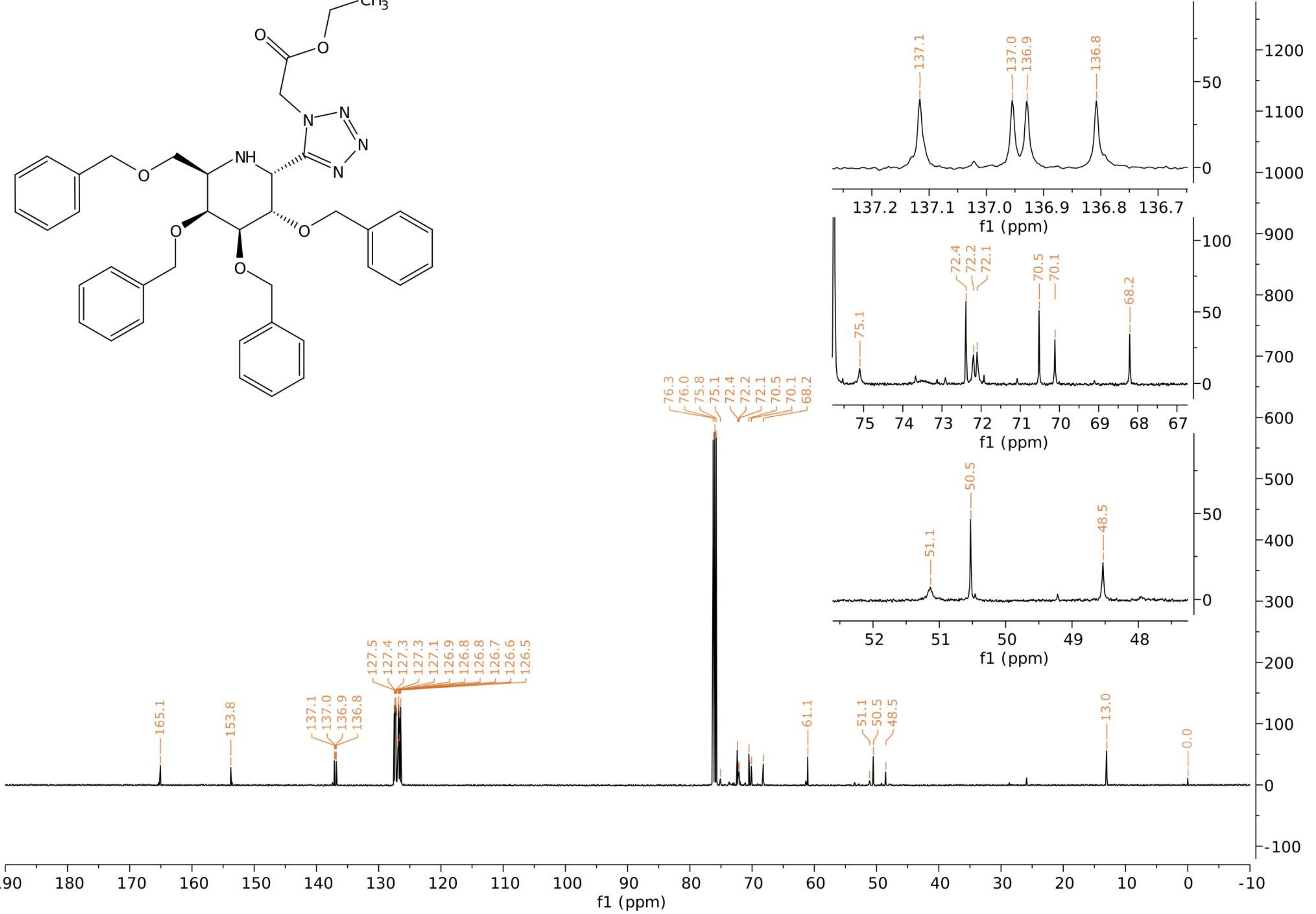

6 — 1H NMR (600 MHz)

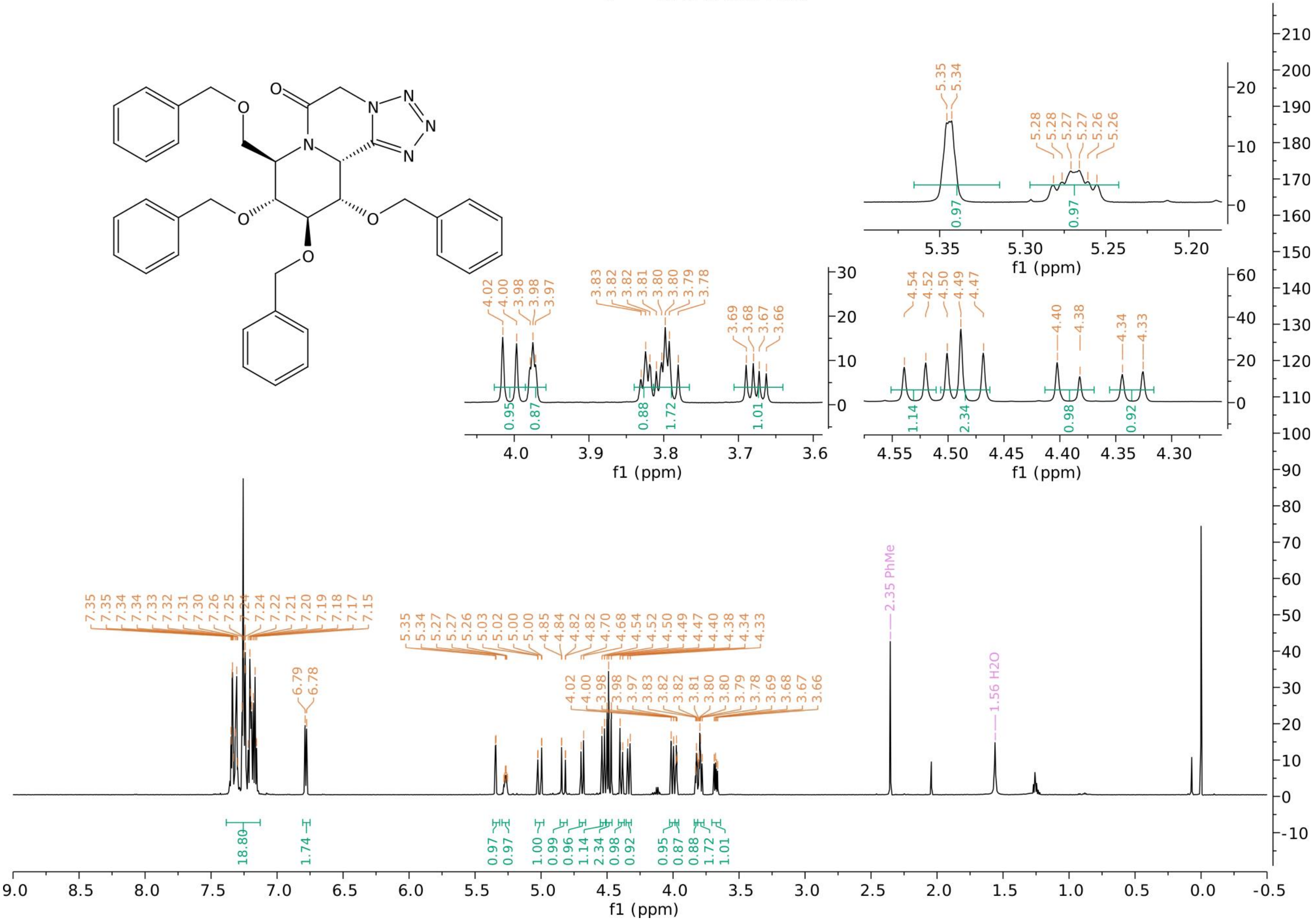

6 — 13C NMR (151 MHz)

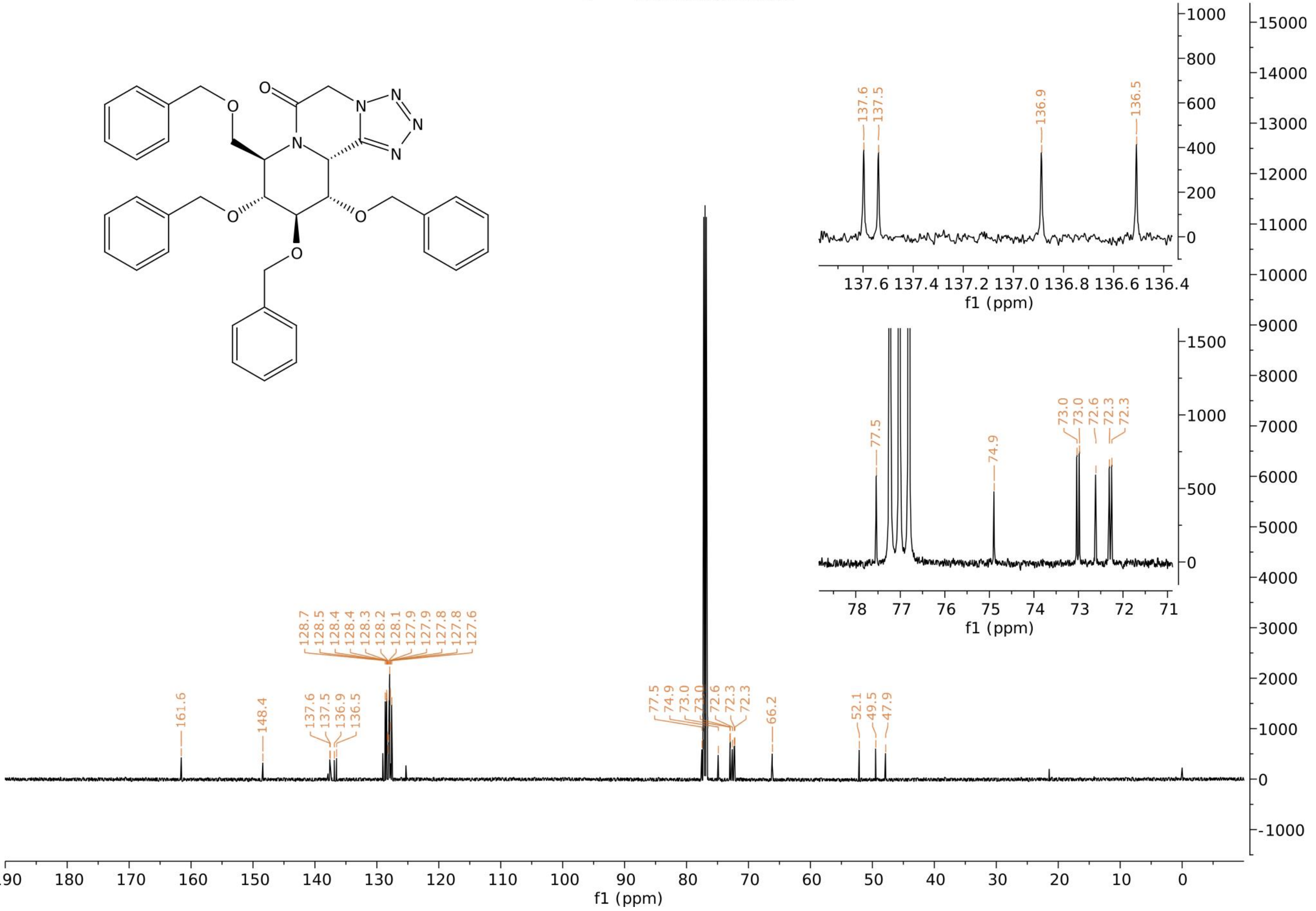

7 — 1H NMR (500 MHz)

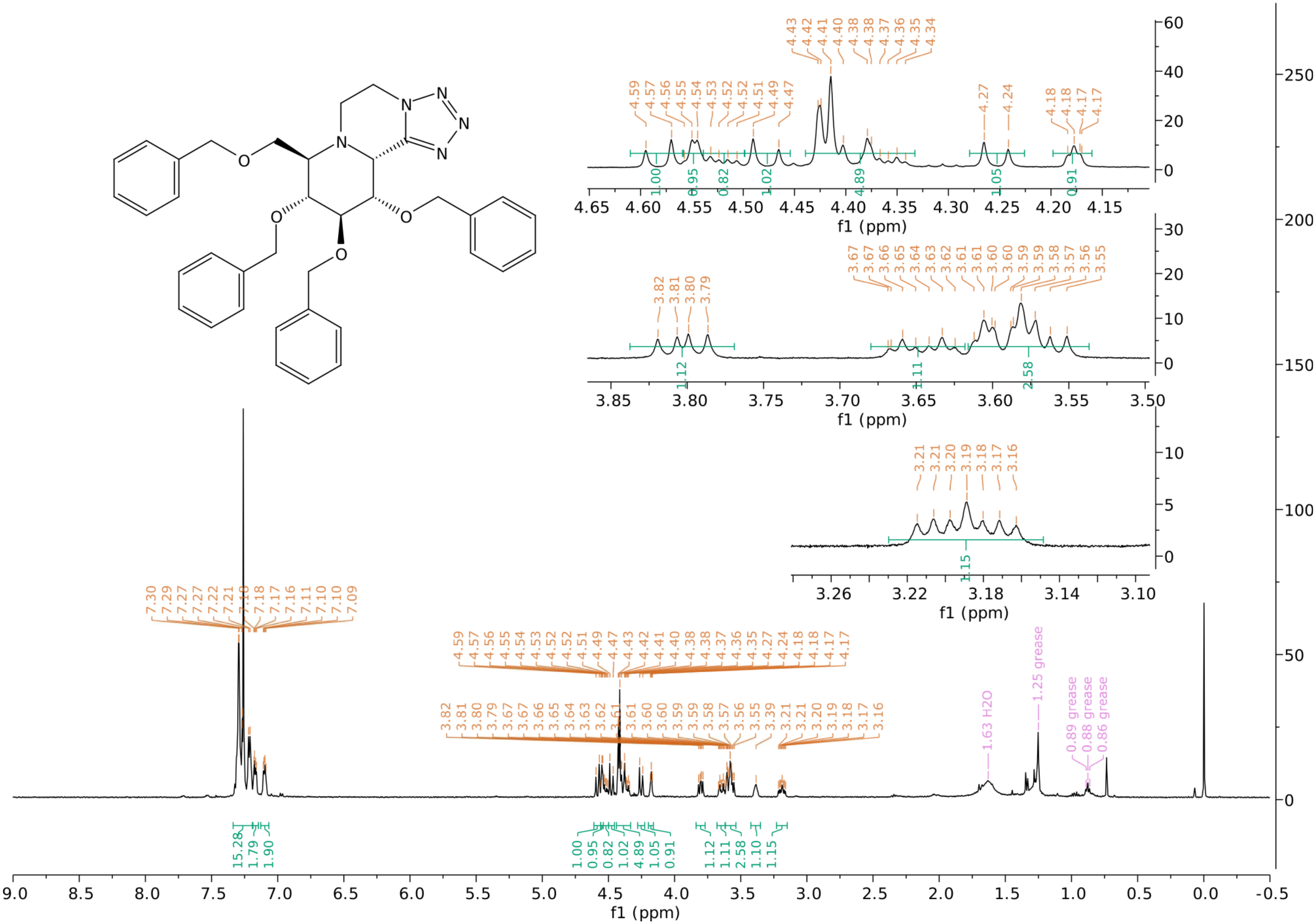

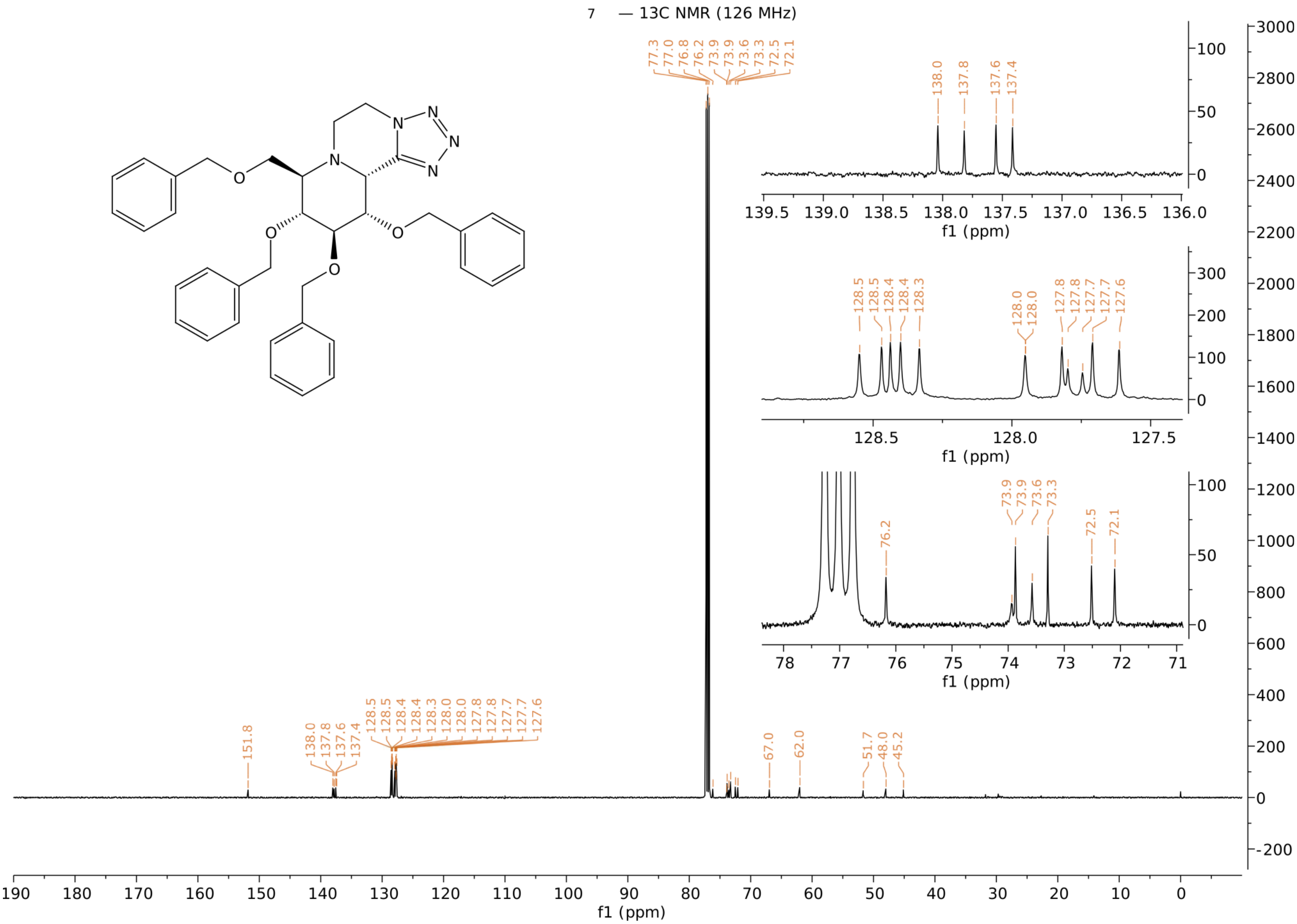
7 — 13C NMR (126 MHz)
77.3 77.0 76.8 76.2 73.9 73.9 73.6 73.3 72.5 72.1
151.8
138.0 137.8 137.6 137.4
128.5 128.5 128.4 128.4 128.3 128.0 128.0 127.8 127.8 127.7 127.7 127.6
67.0
62.0
51.7 48.0 45.2
f1 (ppm)

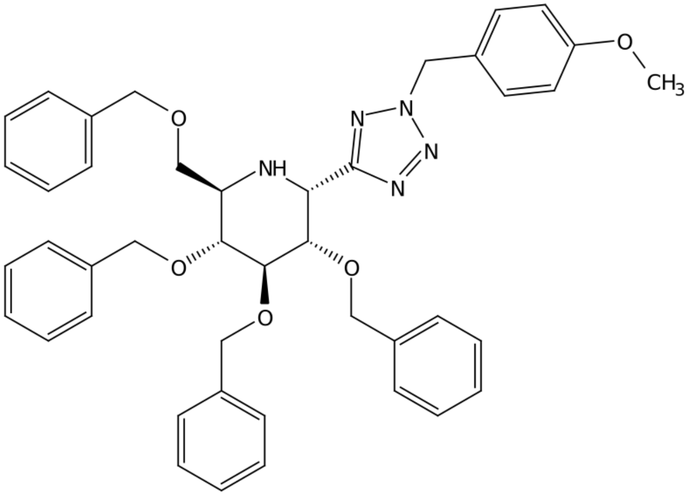

8 — 13C NMR (126 MHz)

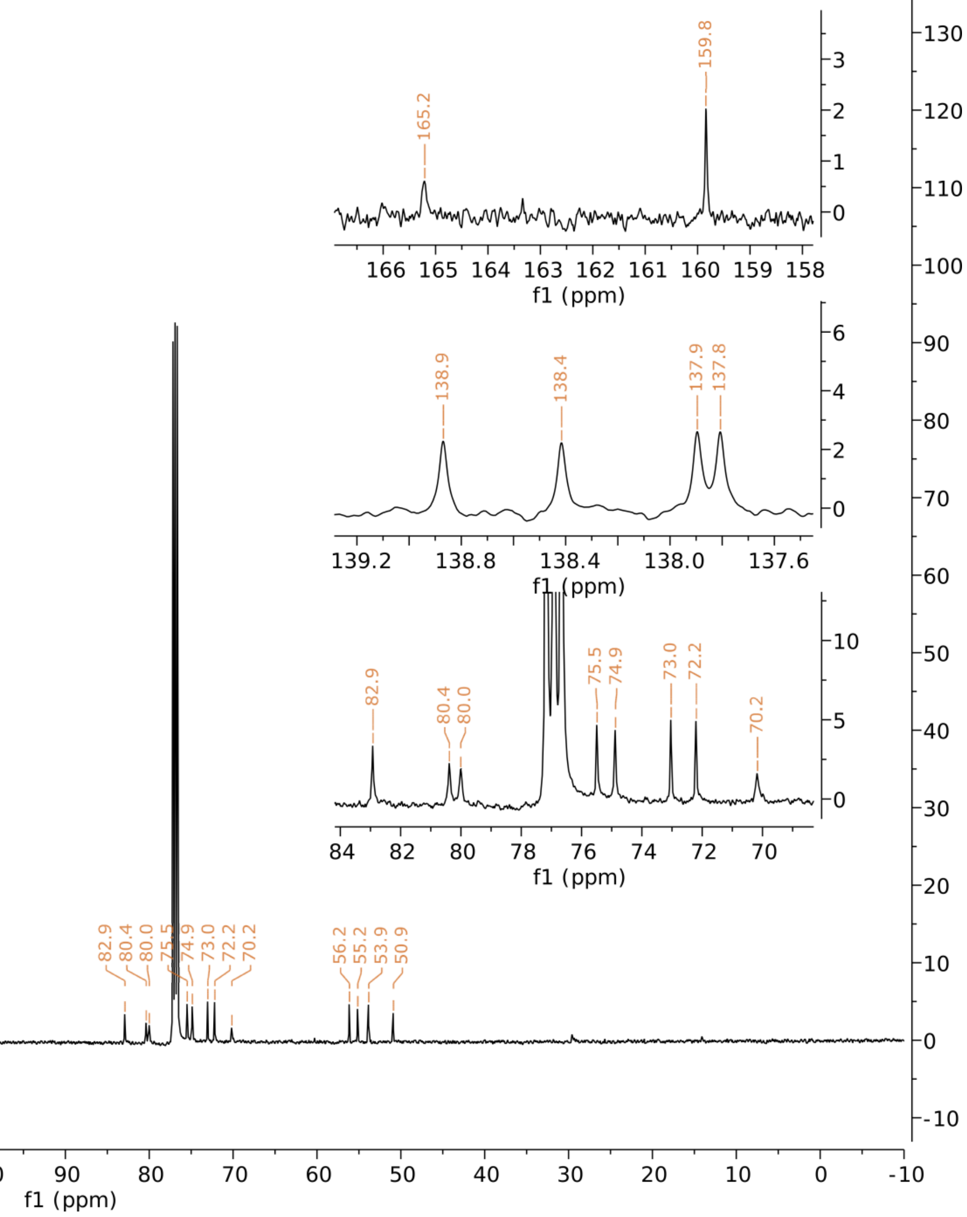

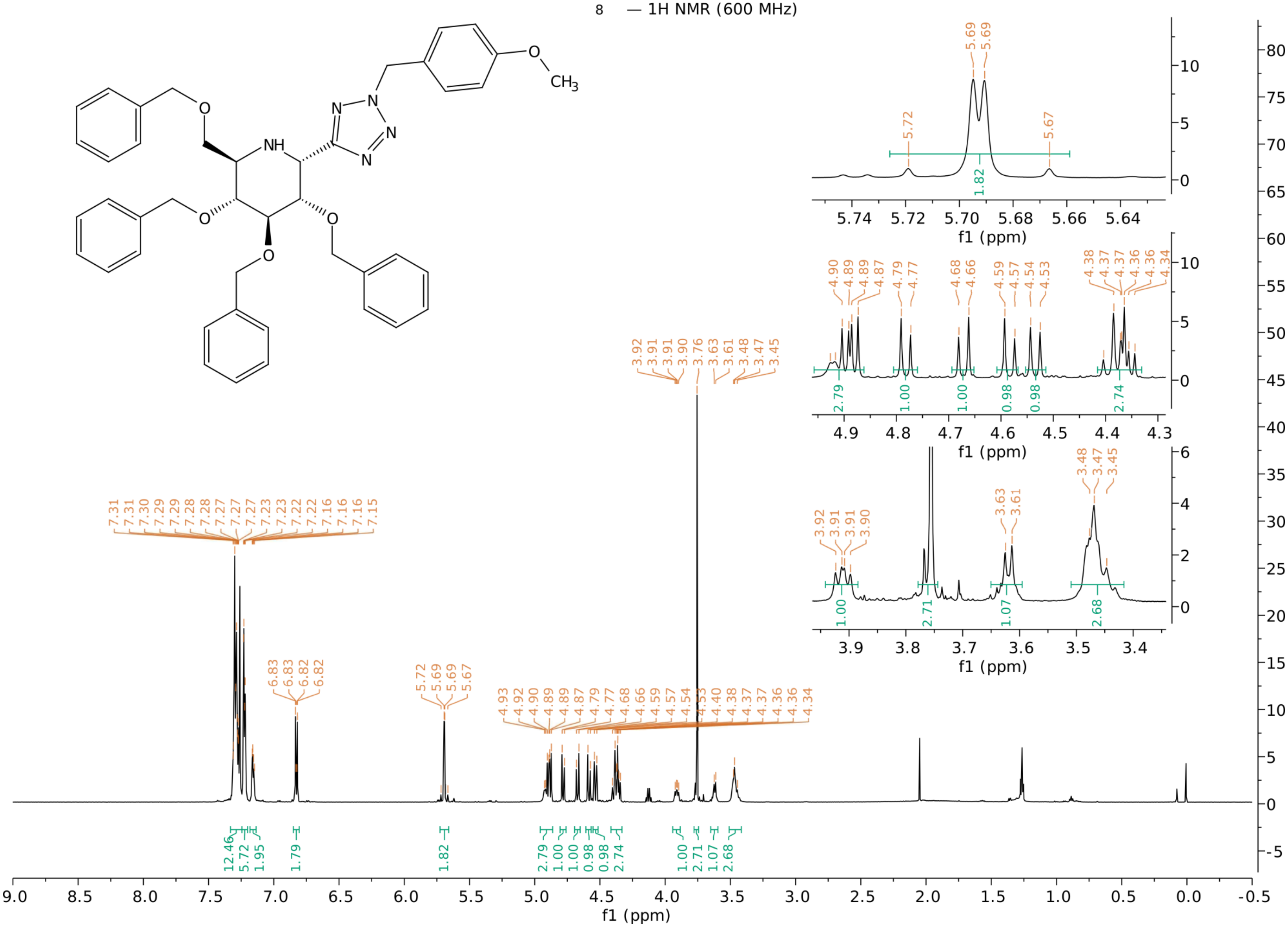

8 — 1H NMR (600 MHz)
7.31 7.31 7.30 7.29 7.29 7.28 7.28 7.27 7.27 7.27 7.23 7.23 7.22 7.22 7.16 7.16 7.16 7.15
6.83 6.83 6.82 6.82
5.72 5.69 5.69 5.67
4.93 4.92 4.90 4.89 4.89 4.87 4.79 4.77 4.68 4.66 4.59 4.57 4.54 4.53 4.40 4.38 4.37 4.37 4.36 4.36 4.34
3.92 3.91 3.91 3.90 3.76 3.63 3.61 3.48 3.47 3.45
12.46 5.72 1.95 1.79 1.82 2.79 1.00 1.00 0.98 0.98 2.74 1.00 2.71 1.07 2.68
f1 (ppm)

9 — 1H NMR (500 MHz)

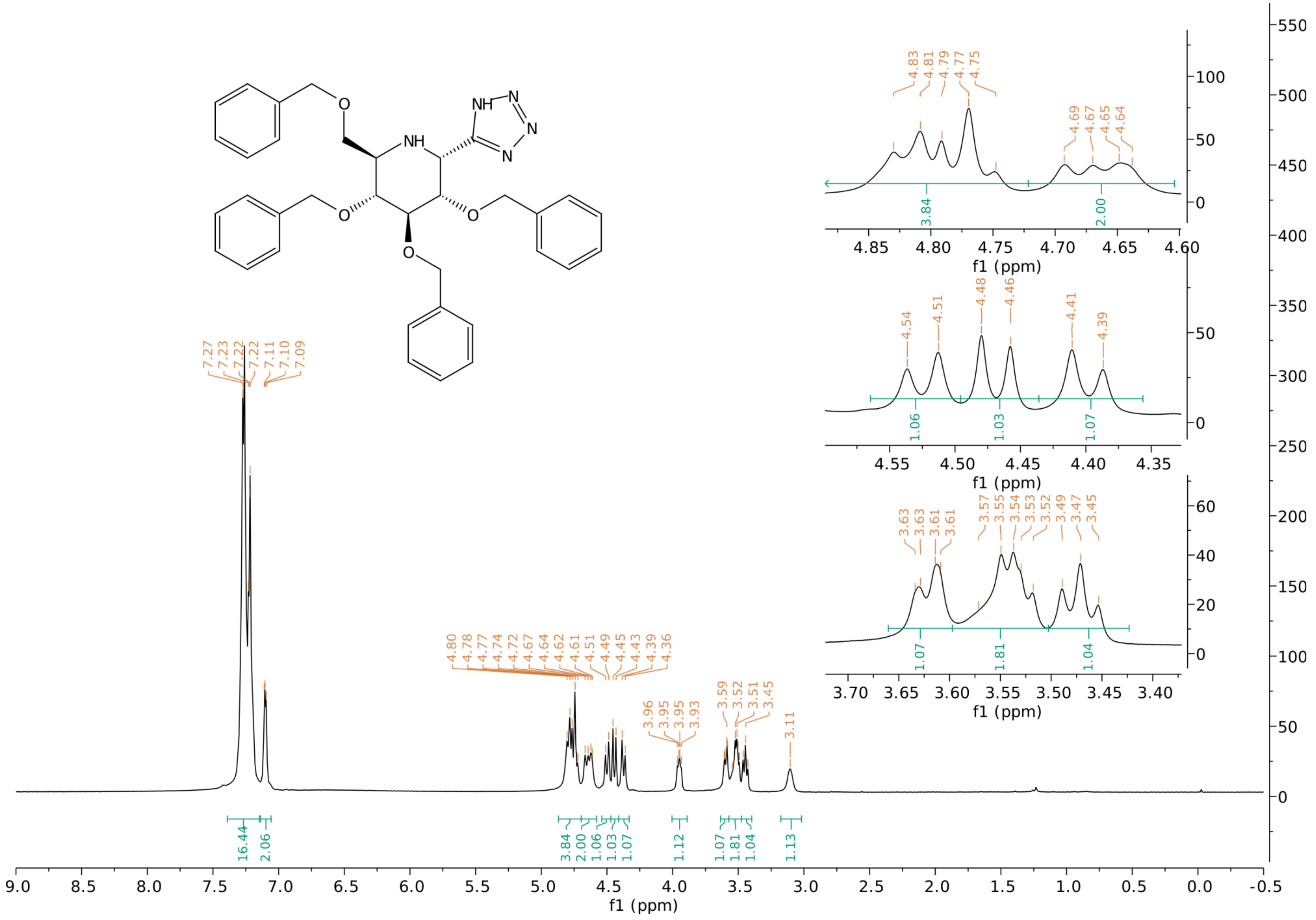

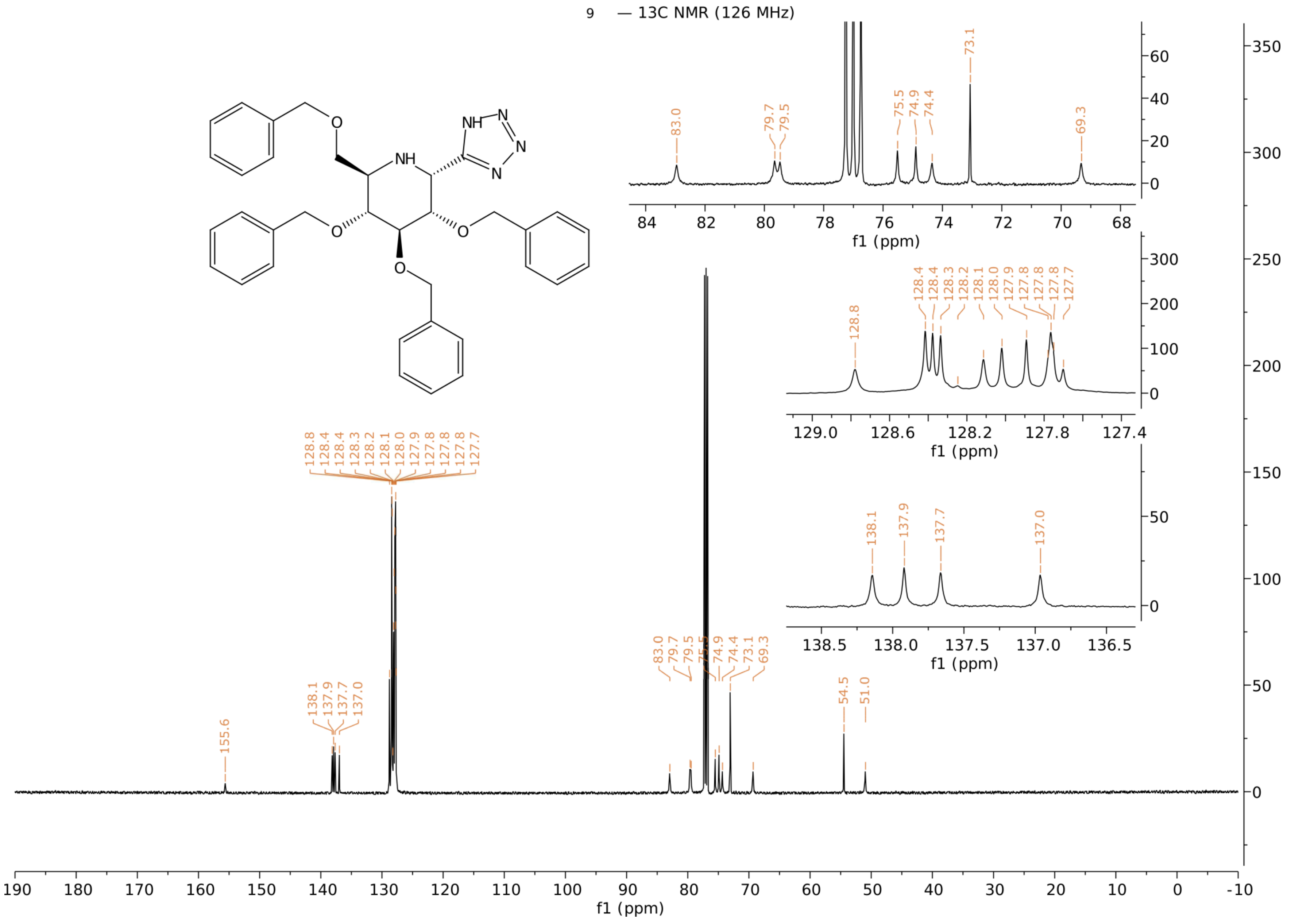

9
— 13C NMR (126 MHz)
155.6
138.1
137.9
137.7
137.0
128.8
128.4
128.4
128.3
128.2
128.1
128.0
127.9
127.8
127.8
127.8
127.7
83.0
79.7
79.5
75.5
74.9
74.4
73.1
69.3
54.5
51.0
f1 (ppm)

5 Computational data

INT-1-A

No. of imaginary frequencies = 0

Total energy = -1221.110627

Zr	0.862482	0.046202	0.022516
C	2.583919	1.903839	0.484495
C	1.621470	1.915929	1.539769
C	1.931623	2.299435	0.702776
H	3.623985	1.623999	0.578842
C	0.391038	2.374474	1.000918
H	1.811008	1.672891	2.576977
C	0.568450	2.580438	0.384228
H	2.370998	2.351202	1.689495
H	0.543978	2.452788	1.537490
H	0.195658	2.874241	1.090366
C	2.238446	2.047904	0.683332
C	1.090444	2.522086	0.008376
C	3.028340	1.311146	0.227472
H	2.440609	2.184665	1.735816
C	1.187202	2.100877	1.357941
H	0.285325	3.099885	0.424899
C	2.371562	1.333052	1.495146
H	3.968277	0.824955	0.005147
H	0.458068	2.285582	2.135361
H	2.731525	0.874143	2.405971
Cl	0.368070	0.098738	2.451081
N	2.671263	1.140836	0.789191
O	0.986748	0.168607	0.588613
C	3.124541	0.669205	1.568711
H	3.885108	1.456974	1.628441
H	2.634019	0.598577	2.545275
C	3.776033	0.664523	1.178548
H	4.563061	0.928199	1.894269
H	3.017577	1.456110	1.224855
C	4.337416	0.582546	0.248750
H	4.747047	1.550069	0.561601
H	5.156750	0.147357	0.278717
C	3.244095	0.142808	1.226296
H	3.658139	0.000139	2.229700
H	2.474344	0.928218	1.297727
C	2.084064	1.092613	0.526848
H	1.695697	2.094291	0.760787

H	1.960158	1.428627	1.457689

TS-1-A

No. of imaginary frequencies = 1 (-108.168)

Total energy = -1221.074579

Zr	0.941712	0.017547	0.120112
C	2.417408	2.122020	0.415284
C	1.880074	2.266232	0.899263
C	1.351952	2.171633	1.330677
H	3.457306	1.949454	0.655178
C	0.483081	2.486769	0.774970
H	2.445121	2.274617	1.822381
C	0.147809	2.388795	0.591042
H	1.416085	2.036575	2.401789
H	0.204666	2.612038	1.599991
H	0.841261	2.457777	1.022763
C	2.281246	2.017631	0.973605
C	1.777492	2.477812	0.277165
C	3.250326	1.024841	0.718951
H	1.944685	2.344110	1.947469
C	2.441690	1.770063	1.304369
H	1.005788	3.223278	0.413240
C	3.331629	0.846149	0.691908
H	3.795253	0.462765	1.465151
H	2.258682	1.867514	2.365547
H	3.985807	0.156730	1.209600
Cl	0.625619	0.650862	1.816800
N	2.808643	1.135752	1.115556
O	0.227560	0.351160	1.515510
C	3.247708	1.235833	0.738759
H	4.051441	1.629400	1.380379
H	2.490758	2.025882	0.692309
C	3.764954	0.848579	0.653546
H	4.436383	1.625555	1.029679
H	2.913954	0.771462	1.337218
C	4.480983	0.507473	0.588013
H	4.849663	0.801256	1.574830
H	5.350661	0.445142	0.079397
C	3.523492	1.590324	0.090371
H	4.053520	2.510397	0.173432
H	2.758460	1.815476	0.840392
C	2.630733	0.097023	1.470378
H	2.028495	0.265349	2.351993

H	2.170465	1.790921	1.562384

INT-2-A

No. of imaginary frequencies = 0

Total energy = -1221.106694

Zr	1.058567	0.017639	0.065847
C	1.984236	2.313115	0.544170
C	1.522364	2.374637	0.803606
C	0.865434	2.115661	1.380538
H	3.015710	2.374125	0.862805
C	0.105019	2.272533	0.782105
H	2.135994	2.519248	1.682952
C	0.299578	2.088345	0.557003
H	0.881033	1.980718	2.453285
H	0.537800	2.264220	1.650815
H	1.307566	1.917951	0.905435
C	2.669713	1.817100	0.862632
C	2.309289	2.240479	0.444891
C	3.419093	0.621514	0.747449
H	2.376731	2.296283	1.785707
C	2.853651	1.318083	1.371430
H	1.692452	3.094137	0.688714
C	3.527813	0.306875	0.639397
H	3.831186	0.047670	1.566753
H	2.716051	1.344122	2.443380
H	4.051600	0.541363	1.059365
Cl	0.361015	0.987252	1.743086
N	2.669742	0.056998	1.388137
O	0.017023	0.603510	1.626459
C	4.242172	0.994596	0.241014
H	5.231586	1.228558	0.180143
H	3.995953	1.835528	0.902138
C	4.267507	0.337617	1.000057
H	5.141984	0.389134	1.656648
H	3.374168	0.412993	1.631721
C	4.259048	1.484266	0.017040
H	4.291508	2.458015	0.483082
H	5.152471	1.418001	0.652949
C	2.996895	1.398507	0.881176
H	3.068741	2.055440	1.755153
H	2.119676	1.729811	0.313105
C	3.239544	0.968049	0.889492
H	2.954387	1.938303	1.309469

H	0.889554	0.253482	1.803824

INT-1-B

No. of imaginary frequencies = 0

Total energy = -1221.10603

Zr	0.953677	0.009751	0.040892
C	2.482332	1.087275	1.678081
C	1.823204	2.175717	1.048854
C	3.363852	0.497629	0.721031
H	2.377536	0.792353	2.713912
C	2.239760	2.221170	0.298855
H	1.061195	2.797802	1.496661
C	3.203619	1.188318	0.498686
H	4.019003	0.344874	0.895091
H	1.874549	2.897429	1.059080
H	3.692224	0.956373	1.434733
C	0.577582	2.508894	0.566277
C	0.466690	2.125870	0.306263
C	1.808357	2.417917	0.139420
H	0.466604	2.767224	1.609159
C	0.114395	1.804259	1.564548
H	1.504241	1.999674	0.030200
C	1.516012	2.003114	1.466580
H	2.789130	2.642859	0.258344
H	0.418079	1.462770	2.442489
H	2.235750	1.852044	2.259173
Cl	0.878505	0.013057	2.523870
N	2.530761	0.054799	0.945534
O	0.731299	0.980921	0.237445
C	2.837831	0.654288	1.400527
H	2.501218	0.341910	1.709557
H	2.531883	1.367480	2.173468
C	4.361172	0.662403	1.199179
H	4.870244	0.354458	2.119338
H	4.690418	1.689339	0.986629
C	4.758859	0.245215	0.024740
H	5.833961	0.169521	0.174887
H	4.542825	1.290953	0.277332
C	3.966433	0.130685	1.232170
H	4.191736	0.558501	2.052622
H	4.277756	1.141875	1.560461
C	2.120621	1.002672	0.092550
H	2.396208	2.041830	0.187402

H	1.978406	0.239179	1.781230

TS-1-B

No. of imaginary frequencies = 1 (-386.7343)

Total energy = -1221.010227

Zr	1.044381	0.070195	0.090005
C	2.068187	1.688861	1.421622
C	0.738693	2.118224	1.146342
C	2.806283	1.744485	0.212307
H	2.450054	1.390509	2.387541
C	0.671508	2.466170	0.228866
H	0.069259	2.190109	1.860794
C	1.938460	2.222911	0.808778
H	3.840252	1.455847	0.078716
H	0.210283	2.799416	0.757508
H	2.195673	2.337241	1.852354
C	0.546574	2.535085	0.299828
C	0.451798	1.826616	1.017083
C	1.804173	2.292817	0.920274
H	0.382909	3.148978	0.575795
C	0.195231	1.098778	2.047904
H	1.494731	1.747567	0.711931
C	1.592997	1.399138	1.996417
H	2.757124	2.687238	0.595697
H	0.287121	0.454348	2.771069
H	2.350514	1.036728	2.677493
Cl	2.469868	0.838489	1.919693
N	2.745238	0.789069	1.032825
O	0.774843	0.097773	1.162264
C	2.510668	1.170026	0.453755
H	2.065014	0.570921	1.255358
H	2.120563	2.194008	0.519603
C	4.051762	1.180293	0.594015
H	4.334004	1.608233	1.562770
H	4.465764	1.850521	0.174226
C	4.654692	0.223507	0.408057
H	5.750729	0.164477	0.448550
H	4.326470	0.871254	1.231764
C	4.180998	0.842712	0.928847
H	4.514622	1.885088	0.995957
H	4.698893	0.296521	1.751441
C	2.228843	0.505407	0.893824
H	2.442075	1.240720	1.705785

H	1.244637	0.808246	1.469096

INT-2-B

No. of imaginary frequencies = 0

Total energy = -1221.009687

Zr	1.047397	0.073710	0.079296
C	2.064530	1.678207	1.440723
C	0.751631	2.130877	1.127174
C	2.834975	1.707769	0.250041
H	2.415812	1.383080	2.419173
C	0.726585	2.465407	0.253104
H	0.072939	2.226204	1.819563
C	2.003437	2.193282	0.797365
H	3.866840	1.399553	0.146671
H	0.134685	2.809332	0.808104
H	2.289377	2.292355	1.834983
C	0.513667	2.527251	0.296962
C	0.507585	1.806886	0.969545
C	1.747724	2.293861	0.966282
H	0.380538	3.143360	0.582324
C	0.104815	1.082631	2.024740
H	1.536289	1.713707	0.613145
C	1.500576	1.394440	2.030831
H	2.709293	2.699709	0.683334
H	0.400077	0.432395	2.727052
H	2.232682	1.036998	2.741725
Cl	2.475977	0.880604	1.888693
N	2.770828	0.804426	1.061316
O	0.746447	0.123467	1.239108
C	2.475958	1.197638	0.367308
H	2.005104	0.629024	1.176272
H	2.071594	2.218017	0.372797
C	4.006675	1.230461	0.575858
H	4.243223	1.703944	1.535766
H	4.452493	1.866384	0.203840
C	4.612554	0.178505	0.476399
H	5.705847	0.123654	0.566553
H	4.241835	0.793830	1.307403
C	4.199238	0.838225	0.860493
H	4.542177	1.879892	0.881600
H	4.761232	0.313233	1.668886
C	2.250847	0.473847	0.965286
H	2.457169	1.193362	1.795336

H	1.060529	0.756704	1.610368

INT-3

No. of imaginary frequencies = 0

Total energy = -250.661194

N	0.658652	1.359350	0.054364
C	1.464563	0.034345	0.102710
H	1.856358	0.069693	1.125794
H	2.343894	0.184323	0.536337
C	0.676313	1.216567	0.304853
H	1.211381	2.125269	0.009733
H	0.577340	1.245415	1.397762
C	0.718663	1.155476	0.326346
H	1.315251	2.036668	0.064664
H	0.621782	1.147448	1.420272
C	1.440248	0.120130	0.128555
H	2.351511	0.278278	0.460127
H	1.772555	0.016567	1.171570
C	0.607216	1.283458	0.054954
H	1.147606	2.234534	0.121486

TS-3

No. of imaginary frequencies = 1 (-127.6484)

Total energy = -824.174398

N	1.358231	0.092438	0.020950
C	3.592771	1.168797	0.097266
H	3.889659	1.483052	1.109728
H	3.921573	1.981721	0.561833
C	4.230909	0.174740	0.274407
H	5.271086	0.203047	0.060712
H	4.242032	0.287434	1.364973
C	3.413121	1.310276	0.347424
H	3.836119	2.287932	0.098534
H	3.421627	1.223684	1.441091
C	1.973328	1.253547	0.155573
H	1.332657	1.950431	0.388675
H	1.912864	1.521509	1.215990
C	2.096067	1.140586	0.086431
H	1.574116	2.089548	0.192052
Si	0.608231	0.142496	0.021822
C	0.903235	0.780644	1.620371
H	0.662707	1.845425	1.540606

H	1.977571	0.680281	1.803455
H	0.339643	0.352140	2.456672
C	0.954797	1.976519	0.051055
H	0.528142	2.484057	0.922870
H	2.049319	2.022113	0.108728
H	0.634433	2.491878	0.860874
C	0.913548	0.728627	1.605819
H	1.966928	0.535622	1.829564
H	0.780187	1.812759	1.536320
H	0.279160	0.344780	2.412408
N	3.437698	0.189643	0.050020
N	4.599428	0.120112	0.015316
N	5.731978	0.409209	0.075293

INT-4

No. of imaginary frequencies = 0

Total energy = -659.784677

N	0.262727	0.080794	0.023668
C	2.408659	1.317561	0.058409
H	2.681144	1.685616	1.060623
H	2.663555	2.143929	0.617489
C	3.147411	0.019793	0.288646
H	4.181528	0.076673	0.057995
H	3.181428	0.104985	1.376733
C	2.416556	1.166938	0.345650
H	2.908604	2.112190	0.101356
H	2.419382	1.075131	1.438375
C	0.977126	1.228143	0.155872
H	0.392540	1.966618	0.397428
H	0.933283	1.500022	1.215986
C	0.924928	1.184887	0.068570
H	0.339995	2.097363	0.174664
Si	1.631766	0.003862	0.003191
C	2.046618	1.000119	1.522724
H	1.651638	2.019038	1.472080
H	3.135568	1.079054	1.614391
H	1.674377	0.527522	2.437196
C	2.224718	1.773652	0.089406
H	1.869899	2.298852	0.981915
H	3.319438	1.759009	0.143956
H	1.962565	2.359608	0.797300
C	2.041976	0.838026	1.613962
H	3.131307	0.879641	1.726639

H	1.674216	1.867122	1.664859
H	1.645016	0.285343	2.471196

TS-4

No. of imaginary frequencies = 1 (-290.9038)

Total energy = -1098.809155

N	2.670223	0.096093	0.065723
C	3.368167	2.013629	0.956328
H	3.184435	2.603069	1.857127
H	4.412814	1.680468	0.992607
C	3.109135	2.836257	0.325981
H	2.327582	3.576494	0.129244
H	4.015040	3.394543	0.576050
C	2.682090	1.933659	1.506638
H	3.052901	2.329757	2.455873
H	1.589395	1.903717	1.587812
C	3.184803	0.498676	1.325762
H	2.864120	0.127711	2.161847
H	4.281882	0.473693	1.306928
C	2.544580	0.745128	0.994985
H	2.495784	0.248950	1.963132
Si	2.093426	1.818687	0.085469
C	3.522810	2.850209	0.718167
H	3.836080	2.543156	1.721129
H	3.232925	3.904898	0.775538
H	4.390798	2.777145	0.055483
C	1.608928	2.247443	1.676848
H	2.449869	2.173592	2.374053
H	1.258854	3.284914	1.705971
H	0.790216	1.623281	2.050144
C	0.611358	1.894575	1.239882
H	0.261908	2.929170	1.327875
H	0.857245	1.550686	2.249793
H	0.225474	1.293911	0.870684
C	0.792771	1.333330	1.072627
N	0.320172	1.039586	0.866791
C	1.636372	0.710500	0.622866
C	2.179628	0.934168	0.649901
C	2.412510	0.143499	1.651954
C	3.500146	0.589349	0.904499
H	1.567559	1.377265	1.427940
C	3.724355	0.198564	1.394595
H	1.978601	0.016329	2.632839
C	4.284008	0.019906	0.117383

H	3.912082	0.766759	1.889264
H	4.351893	0.635784	2.162536
O	5.566405	0.348390	0.017670
C	6.229113	0.152706	1.272690
H	7.244343	0.518751	1.126859
H	5.742919	0.727767	2.068619
H	6.257111	0.909037	1.541423

INT-5

No. of imaginary frequencies = 0

Total energy = -1098.814285

N	2.553350	0.264493	0.163642
C	2.390463	2.506079	0.977665
H	1.561887	3.066866	1.425230
H	3.257271	2.649489	1.627191
C	2.684546	3.004333	0.448495
H	2.540820	4.087223	0.495141
H	3.732488	2.808458	0.698012
C	1.784049	2.280453	1.450830
H	1.893449	2.693397	2.457363
H	0.731159	2.428845	1.175152
C	2.133097	0.781002	1.488029
H	1.281406	0.208608	1.888606
H	2.972581	0.625556	2.171902
C	2.050740	0.980725	0.974912
H	2.432868	0.530061	1.895629
Si	3.071631	1.446488	0.013957
C	4.127819	1.826912	1.514688
H	3.554261	1.803219	2.446320
H	4.553456	2.831624	1.420232
H	4.959990	1.121696	1.606627
C	4.037443	1.605576	1.590715
H	4.846761	0.870671	1.647851
H	4.494203	2.600310	1.637064
H	3.416251	1.505972	2.487424
C	1.536058	2.547532	0.029042
H	1.811803	3.607307	0.043798
H	0.907963	2.384644	0.854342
H	0.931641	2.359436	0.924398
C	0.557235	0.770358	1.027307
N	0.548015	0.523415	0.769663
C	1.856732	0.198033	0.487242
C	2.841207	1.195335	0.525444

C	2.176421	1.134774	0.160075
C	4.156799	0.866880	0.233110
H	2.572734	2.214204	0.782541
C	3.486282	1.454742	0.128628
H	1.398774	1.889913	0.136911
C	4.490774	0.462136	0.096063
H	4.913354	1.639815	0.263944
H	3.773583	2.468022	0.384302
O	5.724807	0.887388	0.392685
C	6.817788	0.041192	0.382417
H	7.697423	0.542172	0.649937
H	6.664856	0.835213	1.121367
H	6.953940	0.475513	0.613893

TS-5

No. of imaginary frequencies = 1 (-79.4602)

Total energy = -1263.213899

N	2.708886	0.385474	0.323278
C	2.640076	1.243076	2.226403
H	3.422152	0.737031	2.805301
H	1.928793	1.674689	2.940032
C	3.265681	2.309026	1.324283
H	3.809304	3.034627	1.938814
H	2.482807	2.844542	0.775200
C	4.199687	1.628640	0.317329
H	4.636576	2.370949	0.358159
H	5.022540	1.126922	0.844802
C	3.418336	0.612907	0.516691
H	4.101547	0.055197	1.166187
H	2.715992	1.161526	1.154559
C	1.937630	0.138043	1.413541
H	1.689104	0.683272	2.101071
C	0.539652	0.549900	0.983019
N	0.564487	0.485412	0.622341
N	1.241012	2.964350	1.339336
C	1.930600	0.270738	0.515333
C	2.644893	0.711568	0.601005
C	2.566582	0.408818	1.573554
C	4.012579	0.463771	0.664908
H	2.083557	1.205299	1.398248
C	3.924940	0.647396	1.500924
H	1.991162	0.739526	2.432084
C	4.660176	0.211579	0.380586

H	4.560661	0.801676	1.535174
H	4.448876	1.168234	2.294232
C	6.798463	0.088563	0.682360
H	7.808842	0.417654	0.438837
H	6.789698	1.001090	0.805011
H	6.475982	0.562677	1.617048
O	5.983273	0.494824	0.415057
N	0.479036	2.176599	1.785553
N	0.277545	1.373414	2.230608
Si	2.293798	1.924747	0.479301
C	1.042813	2.825157	0.616923
C	1.577072	1.586962	2.180442
C	3.874111	2.943765	0.601339
H	1.416625	2.996255	1.632678
H	0.082423	2.302767	0.681755
H	0.839552	3.809398	0.180829
H	0.838751	0.773792	2.163469
H	2.363295	1.287043	2.881848
H	1.100562	2.486247	2.587773
H	4.657486	2.401661	1.141328
H	4.263620	3.193351	0.390914
H	3.691401	3.879306	1.142075

INT-6

No. of imaginary frequencies = 0

Total energy = -1263.270157

N	1.961257	0.868176	0.429019
C	1.705319	0.863558	2.216482
H	1.479046	0.147585	3.016541
H	1.164010	1.788535	2.448021
C	3.219047	1.105615	2.186849
H	3.557193	1.437947	3.175170
H	3.458668	1.905586	1.477803
C	3.938984	0.181857	1.771165
H	5.021607	0.019308	1.709558
H	3.764229	0.960145	2.525857
C	3.420651	0.676042	0.417037
H	3.885252	1.637698	0.181213
H	3.732205	0.032689	0.364124
C	1.148655	0.256791	0.908181
H	0.163212	0.145386	1.147949
C	0.887900	1.297173	0.187310
N	0.224861	1.625538	0.729517

N	2.085731	1.866191	0.702546
C	1.469000	1.157475	0.274654
C	2.355994	0.615152	1.213464
C	1.901044	1.269512	1.061130
C	3.609678	0.135191	0.833606
H	2.042604	0.555477	2.250666
C	3.154293	0.811639	1.444416
H	1.249424	1.735633	1.793527
C	4.014694	0.228257	0.504098
H	4.258525	0.296960	1.585591
H	3.492552	0.896495	2.471696
C	6.130589	0.796286	0.079172
H	7.008867	1.067508	0.666926
H	6.429697	0.097227	0.713277
H	5.712577	1.701024	0.382316
O	5.218023	0.201094	0.988251
N	1.941656	2.655306	1.655491
N	1.943174	3.393274	2.517219
Si	1.207406	2.076283	0.612126
C	0.491602	2.500363	0.090912
C	1.009530	1.456127	2.388611
C	2.292027	3.621677	0.613285
H	0.426773	2.763450	1.152007
H	1.213724	1.685864	0.016593
H	0.898549	3.366911	0.442950
H	0.385664	0.556940	2.419246
H	1.980800	1.201554	2.827363
H	0.541882	2.213198	3.028657
H	3.231189	3.480632	1.157713
H	2.533882	3.939409	0.406257
H	1.754930	4.443017	1.101053

TS-6

No. of imaginary frequencies = 1 (-256.4598)

Total energy = -1263.250449

N	2.082501	0.765156	0.410877
C	1.916418	1.080222	2.086134
H	1.774525	0.412884	2.945722
H	1.361350	2.002268	2.296481
C	3.413594	1.363817	1.918091
H	3.818339	1.764520	2.854740
H	3.562633	2.123231	1.144267
C	4.141977	0.074940	1.520036

H	5.207911	0.268875	1.351773
H	4.064286	0.659536	2.332866
C	3.527394	0.521310	0.249788
H	4.008007	1.478694	0.028866
H	3.726995	0.150669	0.596044
C	1.283691	0.367579	0.870776
H	0.323010	0.031634	1.203475
C	0.923439	1.339946	0.270134
N	0.241811	1.547455	0.753377
N	2.039296	1.966215	0.887316
C	1.473837	1.090490	0.283232
C	2.358050	0.485156	1.184690
C	1.880755	1.252018	1.055553
C	3.594078	0.002095	0.762011
H	2.057648	0.386797	2.222440
C	3.112893	0.776622	1.480600
H	1.224808	1.762909	1.753238
C	3.976153	0.136752	0.579187
H	4.246525	0.479655	1.482526
H	3.434372	0.892677	2.510053
C	6.075201	0.952302	0.236719
H	6.934709	1.218106	0.853647
H	6.405392	0.292397	0.576360
H	5.645212	1.865543	0.195526
O	5.157968	0.299972	1.102423
N	1.555690	2.630879	1.885646
N	0.553516	2.893389	2.402421
Si	1.295564	2.056797	0.499145
C	0.350317	2.460729	0.331294
C	0.988562	1.563904	2.299274
C	2.410781	3.579062	0.453033
H	0.217593	2.649758	1.401811
H	1.096072	1.669588	0.213153
H	0.767801	3.369302	0.117750
H	0.345034	0.680319	2.358415
H	1.929602	1.320905	2.804960
H	0.505696	2.372160	2.860601
H	3.313239	3.461893	1.061256
H	2.718795	3.814563	0.570915
H	1.864780	4.444157	0.846112

INT-7

No. of imaginary frequencies = 0

Total energy = -1263.293985

N	2.118930	0.766701	0.362750
C	2.218800	1.101189	2.002970
H	2.186230	0.419499	2.861770
H	1.714640	2.029309	2.298690
C	3.679250	1.366728	1.618480
H	4.222530	1.765908	2.482640
H	3.717670	2.123418	0.827660
C	4.330070	0.070858	1.118280
H	5.356420	0.260098	0.782550
H	4.378430	0.659992	1.936480
C	3.518290	0.528052	0.035410
H	3.956020	1.484892	0.334710
H	3.574700	0.146298	0.902710
C	1.422690	0.422929	0.866620
H	0.475320	0.084679	1.291630
C	1.076580	1.393529	0.248210
N	0.179500	1.640139	0.713370
N	0.080920	2.458049	1.802420
N	1.177251	2.690839	1.965140
N	1.923810	2.055379	1.022510
C	1.443740	1.159659	0.271070
C	2.301420	0.547889	1.182760
C	1.817530	1.288309	1.071640
C	3.528210	0.039570	0.757780
H	2.004910	0.471209	2.222420
C	3.028940	0.768249	1.502930
H	1.160690	1.798409	1.768370
C	3.891920	0.137050	0.592850
H	4.180650	0.433890	1.480280
H	3.338330	0.848369	2.538900
C	5.973120	0.988260	0.252260
H	6.815170	1.284180	0.878750
H	6.329110	0.314790	0.537700
H	5.531550	1.882150	0.206490
O	5.052080	0.336990	1.118980
Si	1.203130	2.059911	0.406890
C	0.380360	2.344981	0.581780
H	0.163080	2.475691	1.647190
H	1.107570	1.534981	0.477900
H	0.865150	3.261921	0.227630
C	2.249629	3.629701	0.373760
H	1.644509	4.484171	0.697450

H	3.115289	3.574982	1.041280
H	2.613599	3.838852	0.637310
C	0.764530	1.647271	2.200800
H	0.200020	2.458121	2.675610
H	0.159900	0.737001	2.262930
H	1.669150	1.476991	2.794580

INT-8

No. of imaginary frequencies = 0

Total energy = -854.609153

N	2.344906	0.775454	1.061963
C	1.787277	0.867611	1.345301
H	0.719970	1.009527	1.153713
H	1.883162	0.314115	2.285457
C	2.477063	2.235508	1.440141
H	1.993197	2.847819	2.208746
H	3.520641	2.096541	1.755378
C	2.450905	2.946824	0.080266
H	2.994678	3.896772	0.128445
H	1.414186	3.172668	0.198567
C	3.069473	2.052196	0.996504
H	3.005959	2.528617	1.980297
H	4.142597	1.908281	0.767763
C	2.409484	0.031697	0.210311
H	3.454712	0.184661	0.491845
H	2.744854	0.194365	1.795439
C	1.785261	1.324667	0.049893
N	0.473948	1.662041	0.097611
N	0.405541	3.021788	0.189321
N	1.615432	3.461954	0.102046
N	2.496479	2.440689	0.042052
C	0.735181	0.900354	0.137754
C	1.848756	1.378238	0.551078
C	0.813886	0.283659	0.880517
C	3.051906	0.674343	0.509756
H	1.773868	2.303411	1.110550
C	2.006095	0.992560	0.907167
H	0.069702	0.647644	1.395307
C	3.134138	0.519830	0.218740
H	3.906557	1.062064	1.049581
H	2.093483	1.916403	1.468543
C	5.432882	0.859484	0.341792

H	6.191718	1.611511	0.122194
H	5.288593	0.798792	1.428328
H	5.771838	0.115647	0.031014
O	4.251433	1.291157	0.320277

Benzyl isocyanide

No. of imaginary frequencies = 0

Total energy = -439.03858

C	0.733872	1.158835	0.000074
C	0.652348	1.010897	0.000139
C	1.215554	0.272401	0.000125
C	0.378741	1.400558	0.000014
C	0.998515	1.251987	0.000103
C	1.562787	0.034034	0.000082
H	1.179452	2.147524	0.000128
H	1.275995	1.895917	0.000157
H	0.837284	2.383183	0.000064
H	1.649878	2.118929	0.000256
N	2.938731	0.185625	0.000048
C	4.113129	0.312269	0.000008
C	3.455248	0.563298	0.000289
H	4.454877	0.127321	0.000395
H	3.333258	1.187660	0.894113
H	3.332822	1.187149	0.895035
O	2.550584	0.533644	0.000309

$Cp_2Zr(OH)Cl$

No. of imaginary frequencies = 0

Total energy = -970.431956

Zr	0.005933	0.095094	0.229621
C	1.703633	0.748660	1.485564
C	1.601067	1.759486	0.484567
C	2.312212	0.386103	0.894842
H	1.384306	0.834977	2.515630
C	2.143658	1.240047	0.717673
H	1.196479	2.752724	0.624392
C	2.568234	0.090021	0.469133
H	2.495160	1.332942	1.381760
H	2.173742	1.743804	1.673779
H	2.978475	0.771333	1.201397
C	2.093002	0.034639	1.261280
C	2.564866	0.278949	0.061667

C	1.541278	1.264457	1.296064
H	2.123112	0.740705	2.079248
C	2.314860	0.877909	0.833847
H	3.014993	1.199158	0.407370
C	1.650569	1.825265	0.011274
H	1.098298	1.746343	2.156756
H	2.545542	1.010277	1.882331
H	1.327950	2.812957	0.311955
Cl	0.090544	2.486362	0.378871
O	0.108827	0.237475	2.220219
H	0.672339	0.441921	2.747500

$TMSN_3$

No. of imaginary frequencies = 0

Total energy = -573.536386

Si	0.663505	0.000109	0.020109
C	1.999183	0.002318	1.293089
H	1.920871	0.882848	1.931929
H	1.917474	0.887309	1.931748
H	2.994893	0.004178	0.836627
C	0.718297	1.544763	1.093397
H	0.670082	2.452012	0.482976
H	0.120603	1.567660	1.797502
H	1.643786	1.580032	1.679287
C	0.717288	1.549392	1.087067
H	0.120311	1.573321	1.792696
H	0.665990	2.454102	0.473143
H	1.643813	1.588912	1.671035
N	0.863419	0.002298	0.904471
N	1.970500	0.001011	0.379029
N	3.039463	0.000032	0.026056

N_3^-

No. of imaginary frequencies = 0

Total energy = -164.379062

N	1.169683	0.205645	0.000000
N	0.000000	0.000621	0.000000
N	1.169683	0.205024	0.000000

H_2O

No. of imaginary frequencies = 0

Total energy = -76.448012

O	0.000000	0.000000	0.119179
H	0.000000	0.759328	0.476717
H	0.000000	0.759328	0.476717

Me_3SiOH

No. of imaginary frequencies = 0

Total energy = -485.14991

Si	0.005225	0.000000	0.038191
C	1.765508	0.000005	0.610009
H	2.309233	0.884665	0.263483
H	2.309260	0.884608	0.263405
H	1.784374	0.000043	1.705431
C	0.918670	1.538012	0.543009
H	0.988149	1.560671	1.636806
H	1.941606	1.574560	0.150906
H	0.404342	2.449723	0.221422
C	0.918663	1.538015	0.543011
H	0.404322	2.449724	0.221442
H	1.941590	1.574575	0.150887
H	0.988166	1.560666	1.636807
O	0.160471	0.000004	1.709862
H	0.660666	0.000016	2.213185

6 References

[S1] Szcześniak, P.; Stecko, S.; Staszewska-Krajewska, O.; Furman, B. Tetrahedron 2014, 70, 1880–1888.

[S2] Sheldrick, G. M. SHELXL-2014. Program for the Refinement of Crystal Structures from Diffraction Data. 2014; University of Göttingen, Germany.

[S3] Frisch, M. J.; Trucks, G. W.; Schlegel, H. B.; Scuseria, G. E.; Robb, M. A.; Cheeseman, J. R.; Scalmani, G.; Barone, V.; Mennucci, B.; Petersson, G. A.; Nakatsuji, H.; Caricato, M.; Li, X.; Hratchian, H. P.; Izmaylov, A. F.; Bloino, J.; Zheng, G.; Sonnenberg, J. L.; Hada, M.; Ehara, M.; Toyota, K.; Fukuda, R.; Hasegawa, J.; Ishida, M.; Nakajima, T.; Honda, Y.; Kitao, O.; Nakai, H.; Vreven, T.; Montgomery, J. A., Jr.;

Peralta, J. E.; Ogliaro, F.; Bearpark, M.; Heyd, J. J.; Brothers, E.; Kudin, K. N.; Staroverov, V. N.; Kobayashi, R.; Normand, J.; Raghavachari, K.; Rendell, A.; Burant, J. C.; Iyengar, S. S.; Tomasi, J.; Cossi, M.; Rega, N.; Millam, J. M.; Klene, M.; Knox, J. E.; Cross, J. B.; Bakken, V.; Adamo, C.; Jaramillo, J.; Gomperts, R.; Stratmann, R. E.; Yazyev, O.; Austin, A. J.; Cammi, R.; Pomelli, C.; Ochterski, J. W.; Martin, R. L.; Morokuma, K.; Zakrzewski, V. G.; Voth, G. A.; Salvador, P.; Dannenberg, J. J.; Dapprich, S.; Daniels, A. D.; Farkas, Ö.; Foresman, J. B.; Ortiz, J. V.; Cioslowski, J.; Fox, D. J. Gaussian 09 Revision E.01. 2016; Gaussian Inc. Wallingford CT.

[S4] Frisch, M. J.; Trucks, G. W.; Schlegel, H. B.; Scuseria, G. E.; Robb, M. A.; Cheeseman, J. R.; Scalmani, G.; Barone, V.; Petersson, G. A.; Nakatsuji, H.; Li, X.; Caricato, M.; Marenich, A. V.; Bloino, J.; Janesko, B. G.; Gomperts, R.; Mennucci, B.; Hratchian, H. P.; Ortiz, J. V.; Izmaylov, A. F.; Sonnenberg, J. L.; Williams-Young, D.; Ding, F.; Lipparini, F.; Egidi, F.; Goings, J.; Peng, B.; Petrone, A.; Henderson, T.; Ranasinghe, D.; Zakrzewski, V. G.; Gao, J.; Rega, N.; Zheng, G.; Liang, W.; Hada, M.; Ehara, M.; Toyota, K.; Fukuda, R.; Hasegawa, J.; Ishida, M.; Nakajima, T.; Honda, Y.; Kitao, O.; Nakai, H.; Vreven, T.; Throssell, K.; Montgomery, J. A., Jr.; Peralta, J. E.; Ogliaro, F.; Bearpark, M. J.; Heyd, J. J.; Brothers, E. N.; Kudin, K. N.; Staroverov, V. N.; Keith, T. A.; Kobayashi, R.; Normand, J.; Raghavachari, K.; Rendell, A. P.; Burant, J. C.; Iyengar, S. S.; Tomasi, J.; Cossi, M.; Millam, J. M.; Klene, M.; Adamo, C.; Cammi, R.; Ochterski, J. W.; Martin, R. L.; Morokuma, K.; Farkas, Ö.; Foresman, J. B.; Fox, D. J. Gaussian 16 Revision C.01. 2016; Gaussian Inc. Wallingford CT.
